# Distinct genetic liability profiles define clinically relevant patient strata across common diseases

**DOI:** 10.1038/s41467-024-49338-2

**Published:** 2024-07-01

**Authors:** Lucia Trastulla, Georgii Dolgalev, Sylvain Moser, Laura T. Jiménez-Barrón, Till F. M. Andlauer, Moritz von Scheidt, Schizophrenia Working Group of the Psychiatric Genomics Consortium, Douglas M. Ruderfer, Stephan Ripke, Andrew McQuillin, Eli A. Stahl, Enrico Domenici, Rolf Adolfsson, Ingrid Agartz, Esben Agerbo, Margot Albus, Madeline Alexander, Farooq Amin, Silviu A. Bacanu, Martin Begemann, Richard A. Belliveau, Judit Bene, Sarah E. Bergen, Elizabeth Bevilacqua, Tim B. Bigdeli, Donald W. Black, Douglas H. R. Blackwood, Anders D. Borglum, Elvira Bramon, Richard Bruggeman, Nancy G. Buccola, Randy L. Buckner, Brendan Bulik-Sullivan, Joseph D. Buxbaum, William Byerley, Wiepke Cahn, Guiqing Cai, Dominique Campion, Rita M. Cantor, Vaughan J. Carr, Noa Carrera, Stanley V. Catts, Kimberley D. Chambert, Raymond C. K. Chan, Eric Y. H. Chen, Ronald Y. L. Chen, Wei Cheng, Eric F. C. Cheung, Siow Ann Chong, Sven Cichon, C. Robert Cloninger, David Cohen, Nadine Cohen, David A. Collier, Paul Cormican, Nicholas Craddock, James J. Crowley, Mark J. Daly, Ariel Darvasi, Michael Davidson, Kenneth L. Davis, Franziska Degenhardt, Jurgen Del Favero, Ditte Demontis, Dimitris Dikeos, Timothy Dinan, Srdjan Djurovic, Gary Donohoe, Elodie Drapeau, Jubao Duan, Frank Dudbridge, Hannelore Ehrenreich, Peter Eichhammer, Johan Eriksson, Valentina Escott-Price, Tonu Esko, Laurent Essioux, Kai-How Farh, Martilias S. Farrell, Josef Frank, Lude Franke, Robert Freedman, Nelson B. Freimer, Joseph I. Friedman, Menachem Fromer, Pablo V. Gejman, Giulio Genovese, Lyudmila Georgieva, Ina Giegling, Michael Gill, Paola Giusti-Rodriguez, Stephanie Godard, Jacqueline I. Goldstein, Srihari Gopal, Jacob Gratten, Hugh Gurling, Lieuwe de Haan, Christian Hammer, Marian L. Hamshere, Mark Hansen, Thomas Hansen, Vahram Haroutunian, Annette M. Hartmann, Frans A. Henskens, Stefan Herms, Joel N. Hirschhorn, Per Hoffmann, Andrea Hofman, Mads V. Hollegaard, David M. Hougaard, Hailiang Huang, Christina M. Hultman, Masashi Ikeda, Nakao Iwata, Assen V. Jablensky, Inge Joa, Erik G. Jonsson, Antonio Julia, Anna K. Kahler, René S. Kahn, Luba Kalaydjieva, Sena Karachanak-Yankova, Juha Karjalainen, David Kavanagh, Matthew C. Keller, James L. Kennedy, Andrey Khrunin, Yunjung Kim, George Kirov, Janis Klovins, Jo Knight, James A. Knowles, Bettina Konte, Vaidutis Kucinskas, Zita Ausrele Kucinskiene, Hana Kuzelova-Ptackova, Claudine Laurent, Marion Leboyer, Phil H. Lee, Jimmy Lee Chee Keong, Sophie E. Legge, Todd Lencz, Bernard Lerer, Douglas F. Levinson, Miaoxin Li, Qingqin S. Li, Tao Li, Kung-Yee Liang, Jeffrey Lieberman, Svetlana Limborska, Jianjun Liu, Jouko Lonnqvist, Carmel M. Loughland, Jan Lubinski, Milan Macek, Patrik K. E. Magnusson, Brion S. Maher, Wolfgang Maier, Anil K. Malhotra, Jacques Mallet, Sara Marsal, Manuel Mattheisen, Morten Mattingsdal, Robert W. McCarley, Steven A. McCarroll, Colm McDonald, Andrew M. McIntosh, Sandra Meier, Carin J. Meijer, Bela Melegh, Ingrid Melle, Raquelle I. Mesholam-Gately, Andres Metspalu, Patricia T. Michie, Lili Milani, Vihra Milanova, Younes Mokrab, Jennifer L. Moran, Derek W. Morris, Ole Mors, Preben B. Mortensen, Bryan J. Mowry, Kieran C. Murphy, Robin M. Murray, Inez Myin-Germeys, Benjamin M. Neale, Mari Nelis, Igor Nenadic, Deborah A. Nertney, Gerald Nestadt, Kristin K. Nicodemus, Liene Nikitina-Zake, Laura Nisenbaum, Annelie Nordin, Markus M. Nothen, Eadbhard O’Callaghan, Colm O’Dushlaine, F. Anthony O’Neill, Sang-Yun Oh, Ann Olincy, Line Olsen, Jim Van Os, Michael J. Owen, Aarno Palotie, Christos Pantelis, George N. Papadimitriou, Elena Parkhomenko, Carlos Pato, Michele T. Pato, Tiina Paunio, Diana O. Perkins, Tune H. Pers, Tracey L. Petryshen, Olli Pietilainen, Jonathan Pimm, Andrew J. Pocklington, Danielle Posthuma, John Powell, Alkes Price, Ann E. Pulver, Shaun M. Purcell, Digby Quested, Henrik B. Rasmussen, Abraham Reichenberg, Mark A. Reimers, Alexander L. Richards, Brien P. Riley, Joshua L. Roffman, Panos Roussos, Dan Rujescu, Veikko Salomaa, Alan R. Sanders, Ulrich Schall, Sibylle G. Schwab, Edward M. Scolnick, Rodney J. Scott, Larry J. Seidman, Pak C. Sham, Jianxin Shi, Engilbert Sigurdsson, Jeremy M. Silverman, Kang Sim, Petr Slominsky, Jordan W. Smoller, Hon-Cheong So, Erik Soderman, Chris C. A. Spencer, David St Clair, Hreinn Stefansson, Kari Stefansson, Stacy Steinberg, Elisabeth Stogmann, Richard E. Straub, Eric Strengman, Jana Strohmaier, T. Scott Stroup, Mythily Subramaniam, Jaana Suvisaari, Dragan M. Svrakic, Jin P. Szatkiewicz, Srinivas Thirumalai, Draga Toncheva, Sarah Tosato, Jens Treutlein, Peter M. Visscher, John Waddington, Dermot Walsh, James T. R. Walters, Dai Wang, Qiang Wang, Bradley T. Webb, Daniel R. Weinberger, Mark Weiser, Thomas Werge, Dieter B. Wildenauer, Nigel M. Williams, Stephanie Williams, Stephanie H. Witt, Aaron R. Wolen, Emily H. M. Wong, Brandon K. Wormley, Simon Xi, Clement C. Zai, Xuebin Zheng, Fritz Zimprich, Aiden Corvin, Ayman H. Fanous, Marcella Rietschel, Peter A. Holmans, Ole A. Andreassen, S. Hong Lee, Patrick F. Sullivan, Roel A. Ophoff, Naomi R. Wray, Pamela Sklar, Kenneth S. Kendler, Michael C. O’Donovan, Monika Budde, Urs Heilbronner, Sergi Papiol, Alexander Teumer, Georg Homuth, Henry Völzke, Marcus Dörr, Peter Falkai, Thomas G. Schulze, Julien Gagneur, Francesco Iorio, Bertram Müller-Myhsok, Heribert Schunkert, Michael J. Ziller

**Affiliations:** 1 Max Planck Institute of Psychiatry, Munich, Germany; 2 Technische Universität München Medical Graduate Center Experimental Medicine, Munich, Germany; 3 Human Technopole, Milan, Italy; 4 Department of Psychiatry, University of Münster, Münster, Germany; 5 International Max Planck Research School for Translational Psychiatry (IMPRS-TP), Munich, Germany; 6 Department of Neurology, Klinikum rechts der Isar, School of Medicine, Technical University of Munich, Munich, Germany; 7 Klinik für Herz-und Kreislauferkrankungen, Deutsches Herzzentrum München, Technical University Munich, Munich, Germany; 8 German Center for Cardiovascular Research (DZHK), Partner Site Munich Heart Alliance, Munich, Germany; 9 Institute of Psychiatric Phenomics and Genomics (IPPG), LMU University Hospital, LMU Munich, Munich, 80336 Germany; 10 German Center for Cardiovascular Research (DZHK), Partner Site Greifswald, Greifswald, Germany; 11 Institute of Community Medicine, University Medicine Greifswald, Greifswald, Germany; 12 Department of Psychiatry and Psychotherapy, University Medicine Greifswald, Greifswald, Germany; 13 Interfaculty Institute for Genetics and Functional Genomics, University Medicine Greifswald, Greifswald, Germany; 14 Department of Internal Medicine B, University Medicine Greifswald, Greifswald, Germany; 15 Department of Psychiatry and Psychotherapy, University Hospital, LMU Munich, Munich, 80336 Germany; 16 Department of Psychiatry and Behavioral Sciences, SUNY Upstate Medical University, Syracuse, NY USA; 17 Department of Psychiatry and Behavioral Sciences, Johns Hopkins University School of Medicine, Baltimore, MD USA; 18 School of Computation, Information and Technology, Technical University of Munich, Garching, Germany; 19 Institute of Human Genetics, School of Medicine and Health, Technical University of Munich, Munich, Germany; 20 Computational Health Center, Helmholtz Center Munich, Neuherberg, Germany; 21 Institute of Translational Medicine, University of Liverpool, Liverpool, UK; 22 Center for Soft Nanoscience, University of Münster, Münster, Germany; 23 Departments of Medicine, Psychiatry, Biomedical Informatics, Vanderbilt University Medical Center, Nashville, TN USA; 24 Analytic and Translational Genetics Unit, Massachusetts General Hospital, Boston, Massachusetts USA; 25 Department of Psychiatry and Psychotherapy, Charité – Universitätsmedizin, Berlin, Germany; 26 Stanley Center for Psychiatric Research, Broad Institute of MIT and Harvard, Cambridge, Massachusetts, USA; 27 Molecular Psychiatry Laboratory, Division of Psychiatry, University College London, London, UK; 28 Department of Genetics and Genomic Sciences, Icahn School of Medicine at Mount Sinai, New York, NY USA; 29 Centre for Integrative Biology, University of Trento, Trento, Italy; 30 Department of Clinical Sciences, Psychiatry, Umea University, Umea, Sweden; 31 Department of Clinical Neuroscience, Psychiatry Section, Karolinska Institutet, Stockholm, Sweden; 32 Department of Psychiatry, Diakonhjemmet Hospital, Oslo, Norway; 33 NORMENT, KG Jebsen Centre for Psychosis Research, Institute of Clinical Medicine, University of Oslo, Oslo, Norway; 34 Centre for Integrative Register-based Research, CIRRAU, Aarhus University, Aarhus, Denmark; 35 National Centre for Register-based Research, Aarhus University, Aarhus, Denmark; 36 The Lundbeck Foundation Initiative for Integrative Psychiatric Research, iPSYCH, Aarhus, Denmark; 37 State Mental Hospital, Haar, Germany; 38 Department of Psychiatry and Behavioral Sciences, Stanford University, Stanford, California USA; 39 Department of Psychiatry and Behavioral Sciences, Atlanta Veterans Affairs Medical Center, Atlanta, Georgia USA; 40 Department of Psychiatry and Behavioral Sciences, Emory University, Atlanta, Georgia USA; 41 Virginia Institute for Psychiatric and Behavioral Genetics, Department of Psychiatry, Virginia Commonwealth University, Richmond, Virginia USA; 42 Clinical Neuroscience, Max Planck Institute of Experimental Medicine, Gottingen, Germany; 43 Department of Medical Genetics, University of Pecs, Pecs, Hungary; 44 Szentagothai Research Center, University of Pecs, Pecs, Hungary; 45 Department of Medical Epidemiology and Biostatistics, Karolinska Institutet, Stockholm, Sweden; 46 Department of Psychiatry, University of Iowa Carver College of Medicine, Iowa City, Iowa USA; 47 Division of Psychiatry, University of Edinburgh, Edinburgh, UK; 48 Centre for Integrative Sequencing, iSEQ, Aarhus University, Aarhus C, Denmark; 49 Department of Biomedicine, Aarhus University, Aarhus C, Denmark; 50 Institute of Cognitive Neuroscience, University College London, London, UK; 51 Institute of Psychiatry at King’s College London, London, UK; 52 Mental Health Sciences Unit, University College London, London, UK; 53 University Medical Center Groningen, Department of Psychiatry, University of Groningen, Groningen, RB The Netherlands; 54 School of Nursing, Louisiana State University Health Sciences Center, New Orleans, Louisiana USA; 55 Athinoula A. Martinos Center, Massachusetts General Hospital, Boston, Massachusetts USA; 56 Center for Brain Science, Harvard University, Cambridge, Massachusetts USA; 57 Department of Psychiatry, Massachusetts General Hospital, Boston, Massachusetts USA; 58 Department of Human Genetics, Icahn School of Medicine at Mount Sinai, New York, New York, USA; 59 Department of Neuroscience, Icahn School of Medicine at Mount Sinai, New York, New York, USA; 60 Department of Psychiatry, Icahn School of Medicine at Mount Sinai, New York, New York, USA; 61 Friedman Brain Institute, Icahn School of Medicine at Mount Sinai, New York, New York, USA; 62 Department of Psychiatry, University of California at San Francisco, San Francisco, California, USA; 63 Psychiatry, University of California San Francisco, San Francisco, California USA; 64 University Medical Center Utrecht, Department of Psychiatry, Rudolf Magnus Institute of Neuroscience, Utrecht, The Netherlands; 65 Centre Hospitalier du Rouvray and INSERM U1079 Faculty of Medicine, Rouen, France; 66 Department of Human Genetics, David Geffen School of Medicine, University of California, Los Angeles, California, USA; 67 Schizophrenia Research Institute, Sydney, Australia; 68 School of Psychiatry, University of New South Wales, Sydney, Australia; 69 MRC Centre for Neuropsychiatric Genetics and Genomics, Institute of Psychological Medicine and Clinical Neurosciences, School of Medicine, Cardiff University, Cardiff, UK; 70 Royal Brisbane and Women’s Hospital, University of Queensland, Brisbane, Australia; 71 Institute of Psychology, Chinese Academy of Science, Beijing, China; 72 Department of Psychiatry, Li Ka Shing Faculty of Medicine, The University of Hong Kong, Hong Kong, China; 73 State Key Laboratory for Brain and Cognitive Sciences, Li Ka Shing Faculty of Medicine, The University of Hong Kong, Hong Kong, China; 74 Department of Computer Science, University of North Carolina, Chapel Hill, North Carolina USA; 75 Castle Peak Hospital, Hong Kong, China; 76 Institute of Mental Health, Singapore, Singapore; 77 Department of Genomics, Life and Brain Center, Bonn, Germany; 78 Division of Medical Genetics, Department of Biomedicine, University of Basel, Basel, Switzerland; 79 Institute of Human Genetics, University of Bonn, Bonn, Germany; 80 Institute of Neuroscience and Medicine (INM-1), Research Center Juelich, Juelich, Germany; 81 Department of Psychiatry, Washington University, St. Louis, Missouri USA; 82 Department of Child and Adolescent Psychiatry, Assistance Publique Hospitaux de Paris, Pierre and Marie Curie Faculty of Medicine and Institute for Intelligent Systems and Robotics, Paris, France; 83 Blue Note Biosciences, Princeton, New Jersey USA; 84 Eli Lilly and Company Limited, Erl Wood Manor, Sunninghill Road, Windlesham, Surrey, UK; 85 Social, Genetic and Developmental Psychiatry Centre, Institute of Psychiatry, King’s College London, London, UK; 86 Neuropsychiatric Genetics Research Group, Department of Psychiatry, Trinity, College Dublin Ireland; 87 National Centre for Mental Health, Cardiff University, Cardiff, UK; 88 Department of Genetics, University of North Carolina, Chapel Hill, North Carolina USA; 89 Medical and Population Genetics Program, Broad Institute of MIT and Harvard, Cambridge, Massachusetts USA; 90 Department of Genetics, The Hebrew University of Jerusalem, Jerusalem, Israel; 91 Sheba Medical Center, Tel Hashomer, Israel; 92 Life&Brain Center, Department of Genomics, University of Bonn, Bonn, Germany; 93 Applied Molecular Genomics Unit, VIB Department of Molecular Genetics, University of Antwerp, Antwerp, Belgium; 94 Department of Biomedicine, Aarhus University, Aarhus, Denmark; 95 First Department of Psychiatry, University of Athens Medical School, Athens, Greece; 96 Department of Psychiatry, University College Cork, Co. Cork, Ireland; 97 Department of Medical Genetics, Oslo University Hospital, Oslo, Norway; 98 Cognitive Genetics and Therapy Group, School of Psychology and Discipline of Biochemistry, National University of Ireland Galway, Co, Galway, Ireland; 99 Department of Psychiatry and Behavioral Neuroscience, University of Chicago, Chicago, Illinois USA; 100 Department of Psychiatry and Behavioral Sciences, NorthShore University HealthSystem, Evanston, Illinois USA; 101 Department of Non-Communicable Disease Epidemiology, London School of Hygiene and Tropical Medicine, London, UK; 102 Department of Psychiatry, University of Regensburg, 93053 Regensburg, Germany; 103 Department of General Practice, Helsinki University Central Hospital, University of Helsinki, Helsinki, Finland; 104 Folkhalsan Research Center, Helsinki, Finland; 105 Biomedicum Helsinki 1, Haartmaninkatu 8, Helsinki, Finland; 106 National Institute for Health and Welfare, Helsinki, Finland; 107 Department of Genetics, Harvard Medical School, Boston, Massachusetts, USA; 108 Division of Endocrinology and Center for Basic and Translational Obesity Research, Boston Children’s Hospital, Boston, Massachusetts, USA; 109 Estonian Genome Center, University of Tartu, Tartu, Estonia; 110 Translational Technologies and Bioinformatics, Pharma Research and Early Development, F.Hoffman-La Roche, Basel, Switzerland; 111 Department of Genetic Epidemiology in Psychiatry, Central Institute of Mental Health, Medical Faculty Mannheim, University of Heidelberg, Heidelberg, Mannheim, Germany; 112 Department of Genetics, University of Groningen, University Medical Centre Groningen, Groningen, The Netherlands; 113 Department of Psychiatry, University of Colorado Denver, Aurora, Colorado, USA; 114 Center for Neurobehavioral Genetics, Semel Institute for Neuroscience and Human Behavior, University of California, Los Angeles, California, USA; 115 Center for Neurobehavioral Genetics, University of California Los Angeles, Los Angeles, California, USA; 116 Division of Psychiatric Genomics, Department of Psychiatry, Icahn School of Medicine at Mount Sinai, New York, New York, USA; 117 Psychiatric and Neurodevelopmental Genetics Unit, Massachusetts General Hospital, Boston, Massachusetts USA; 118 Department of Psychiatry, University of Halle, Halle, Germany; 119 Department of Psychiatry, University of Munich, Munich, Germany; 120 Neuropsychiatric Genetics Research Group, Dept of Psychiatry and Trinity Translational Medicine Institute, Trinity College Dublin, Dublin, Ireland; 121 Departments of Psychiatry and Human and Molecular Genetics, INSERM, Institut de Myologie, Hopital de la Pitie-Salpetriere, Paris, France; 122 Neuroscience Therapeutic Area, Janssen Research and Development, Raritan, New Jersey USA; 123 Queensland Brain Institute, The University of Queensland, Brisbane, Australia; 124 Academic Medical Centre University of Amsterdam, Department of Psychiatry, Amsterdam, The Netherlands; 125 Illumina, La Jolla, California, California, USA; 126 Institute of Biological Psychiatry, Mental Health Centre Sct. Hans, Mental Health Services Copenhagen, Munich, Denmark; 127 J.J. Peters VA Medical Center, Bronx, New York, New York, USA; 128 Priority Research Centre for Health Behaviour, University of Newcastle, Newcastle, Australia; 129 School of Electrical Engineering and Computer Science, University of Newcastle, Newcastle, Australia; 130 Department of Biomedicine, University of Basel, Basel, Switzerland; 131 Institute of Medical Genetics and Pathology, University Hospital Basel, Basel, Switzerland; 132 Section of Neonatal Screening and Hormones, Department of Clinical Biochemistry, Immunology and Genetics, Statens Serum Institut, Copenhagen, Denmark; 133 Department for Congenital Disorders, Statens Serum Institut, Copenhagen, Denmark; 134 Department of Psychiatry, Fujita Health University School of Medicine, Toyoake, Aichi Japan; 135 School of Psychiatry and Clinical Neurosciences, The University of Western Australia, Perth, Australia; 136 Centre for Clinical Research in Neuropsychiatry, School of Psychiatry and Clinical Neurosciences, The University of Western Australia, Medical Research Foundation Building, Perth, Australia; 137 The Perkins Institute for Medical Research, The University of Western Australia, Perth, Australia; 138 Regional Centre for Clinical Research in Psychosis, Department of Psychiatry, Stavanger University Hospital, Stavanger, Norway; 139 Rheumatology Research Group, Vall d’Hebron Research Institute, Barcelona, Spain; 140 Centre for Medical Research, The University of Western Australia, Perth, Western Australia Australia; 141 Western Australian Institute for Medical Research, The University of Western Australia, Perth, Western Australia Australia; 142 Department of Medical Genetics, Medical University, Sofia, Bulgaria; 143 Department of Psychology, University of Colorado Boulder, Boulder, Colorado, USA; 144 Campbell Family Mental Health Research Institute, Centre for Addiction and Mental Health, Toronto, Ontario, Canada; 145 Department of Psychiatry, University of Toronto, Toronto, Ontario Canada; 146 Institute of Medical Science, University of Toronto, Toronto, Ontario Canada; 147 Institute of Molecular Genetics, Russian Academy of Sciences, Moscow, Russia; 148 Latvian Biomedical Research and Study Centre, Riga, Latvia; 149 Cell Biology, SUNY Downstate Medical Center College of Medicine, Brooklyn, NY USA; 150 Department of Psychiatry and Zilkha Neurogenetics Institute, Keck School of Medicine at University of Southern California, Los Angeles, California, USA; 151 Institute for Genomic Health, SUNY Downstate Medical Center College of Medicine, Brooklyn, NY USA; 152 Faculty of Medicine, Vilnius University, Vilnius, Lithuania; 153 Department of Biology and Medical Genetics, 2nd Faculty of Medicine and University Hospital Motol, Prague, Czech Republic; 154 Department of Child and Adolescent Psychiatry, Pierre and Marie Curie Faculty of Medicine, Paris, France; 155 Department of Psychiatry and Addiction Medicine, Assistance Publique - Hôpitaux de Paris, Paris, France; 156 Faculté de Médecine, Université Paris Est, Créteil, France; 157 INSERM, Paris, France; 158 Duke-NUSA Graduate Medical School, Singapore, Singapore; 159 Hofstra Northwell School of Medicine, Hempstead, New York USA; 160 The Feinstein Institute for Medical Research, Manhasset, New York, USA; 161 The Hofstra NS-LIJ School of Medicine, Hempstead, New York, USA; 162 Department of Psychiatry, Hadassah-Hebrew University Medical Center, Jerusalem, Israel; 163 Centre for Genomic Sciences, The University of Hong Kong, Hong Kong, China; 164 Mental Health Centre and Psychiatric Laboratory, West China Hospital, Sichuan University, Chengdu, Sichuan China; 165 Department of Biostatistics, Johns Hopkins University Bloomberg School of Public Health, Baltimore, Maryland USA; 166 Department of Psychiatry, Columbia University, New York, New York USA; 167 Human Genetics, Genome Institute of Singapore, A*STAR, Singapore, Singapore; 168 Saw Swee Hock School of Public Health, National University of Singapore, Singapore, Singapore; 169 Department of Mental Health and Substance Abuse Services, National Institute for Health and Welfare, Helsinki, Finland; 170 Priority Centre for Translational Neuroscience and Mental Health, University of Newcastle, Newcastle, Australia; 171 Department of Genetics and Pathology, International Hereditary Cancer Center, Pomeranian Medical University in Szczecin, Szczecin, Poland; 172 Department of Mental Health, Bloomberg School of Public Health, Johns Hopkins University, Baltimore, Maryland USA; 173 Department of Psychiatry and Psychotherapy, University of Bonn, Bonn, Germany; 174 The Zucker Hillside Hospital, Glen Oaks, New York, USA; 175 Centre National de la Recherche Scientifique, Laboratoire de Genetique Moleculaire de la Neurotransmission et des Processus Neurodegeneratifs, Hopital de la Pitie Salpetriere, 75013 Paris, France; 176 Department of Clinical Neuroscience, Centre for Psychiatry Research, Karolinska Institutet, Stockholm, Sweden; 177 Department of Genomics Mathematics, University of Bonn, D-53127 Bonn, Germany; 178 iSEQ, Centre for Integrative Sequencing, Aarhus University, Aarhus, Denmark; 179 Stockholm Health Care Services, Stockholm County Council, Stockholm, Sweden; 180 Research Unit, Sorlandet Hospital, Kristiansand, Norway; 181 Department of Psychiatry, Harvard Medical School, Boston, Massachusetts, USA; 182 VA Boston Health Care System, Brockton, Massachusetts, USA; 183 Department of Psychiatry, National University of Ireland Galway, Co, Galway, Ireland; 184 Centre for Cognitive Ageing and Cognitive Epidemiology, University of Edinburgh, Edinburgh, UK; 185 Division of Mental Health and Addiction, Oslo University Hospital, Oslo, Norway; 186 Massachusetts Mental Health Center Public Psychiatry Division of the Beth Israel Deaconess Medical Center, Boston, Massachusetts, USA; 187 Institute of Molecular and Cell Biology, University of Tartu, Tartu, Estonia; 188 School of Psychology, University of Newcastle, Newcastle, Australia; 189 First Psychiatric Clinic, Medical University, Sofia, Bulgaria; 190 Discipline of Biochemistry, Neuroimaging and Cognitive Genomics (NICOG) Centre, National University of Ireland, Galway, Galway Ireland; 191 Queensland Centre for Mental Health Research, University of Queensland, Brisbane, Australia; 192 Department of Psychiatry, Royal College of Surgeons in Ireland, Dublin, Ireland; 193 King’s College London, London, UK; 194 Maastricht University Medical Centre, South Limburg Mental Health Research and Teaching Network, EURON, Maastricht, The Netherlands; 195 Department of Psychiatry and Psychotherapy, Jena University Hospital, Jena, Germany; 196 Department of Psychiatry, Trinity College Dublin, Dublin, Ireland; 197 Eli Lilly and Company, Lilly Corporate Center, Indianapolis, Indiana, USA; 198 DETECT Early Intervention Service for Psychosis, Blackrock, Co, Dublin, Ireland; 199 Centre for Public Health, Institute of Clinical Sciences, Queen’s University Belfast, Belfast, UK; 200 Lawrence Berkeley National Laboratory, University of California at Berkeley, Berkeley, California, USA; 201 Institute for Molecular Medicine Finland, FIMM, University of Helsinki, Helsinki, Finland; 202 Melbourne Neuropsychiatry Centre, University of Melbourne & Melbourne Health, Melbourne, Australia; 203 College of Medicine Institute for Genomic Health, SUNY Downstate Medical Center College of Medicine, Brooklyn, NY USA; 204 Department of Psychiatry, University of Helsinki, Helsinki, Finland; 205 Public Health Genomics Unit, National Institute for Health and Welfare, Helsinki, Finland; 206 Department of Psychiatry, University of North Carolina at Chapel Hill, Chapel Hill, North Carolina USA; 207 Center for Biological Sequence Analysis, Department of Systems Biology, Technical University of Denmark, Munich, Denmark; 208 Center for Human Genetic Research and Department of Psychiatry, Massachusetts General Hospital, Boston, Massachusetts, USA; 209 Department of Child and Adolescent Psychiatry, Erasmus University Medical Centre, Rotterdam, The Netherlands; 210 Department of Complex Trait Genetics, Neuroscience Campus Amsterdam, VU University Medical Center Amsterdam, Amsterdam, The Netherlands; 211 Department of Functional Genomics, Center for Neurogenomics and Cognitive Research, Neuroscience Campus Amsterdam, VU University, Amsterdam, The Netherlands; 212 Department of Epidemiology, Harvard School of Public Health, Boston, Massachusetts, USA; 213 Psychiatry, Brigham and Women’s Hospital, Boston, MA USA; 214 Department of Psychiatry, University of Oxford, Oxford, UK; 215 Virginia Institute for Psychiatric and Behavioral Genetics, Virginia Commonwealth University, Richmond, Virginia USA; 216 Virginia Institute for Psychiatric and Behavioral Genetics, Departments of Psychiatry and Human and Molecular Genetics, Virginia Commonwealth University, Richmond, Virginia USA; 217 Institute for Multiscale Biology, Icahn School of Medicine at Mount Sinai, New York, New York, USA; 218 Priority Centre for Translational Neuroscience and Mental Health, University of Newcastle, Newcastle Australia, Schizophrenia Research Institute, Sydney, Australia; 219 Faculty of Science, Medicine & Health, Univeristy of Wollogong, Wollogong, Australia; 220 Hunter New England Health Service, Newcastle, Australia; 221 School of Biomedical Sciences and Pharmacy, University of Newcastle, Callaghan, Australia; 222 Division of Cancer Epidemiology and Genetics, National Cancer Institute, Bethesda, Maryland USA; 223 Faculty of Medicine, Department of Psychiatry, School of Health Sciences, University of Iceland, Reykjavik, Iceland; 224 Research and Development, Bronx Veterans Affairs Medical Center, New York, New York, USA; 225 Wellcome Trust Centre for Human Genetics, Oxford, UK; 226 University of Aberdeen, Institute of Medical Sciences, Aberdeen, UK; 227 deCODE Genetics / Amgen, Reykjavik, Iceland; 228 Faculty of Medicine, University of Iceland, Reykjavik, Iceland; 229 Department of Clinical Neurology, Medical University of Vienna, Wien, Austria; 230 Lieber Institute for Brain Development, Baltimore, Maryland USA; 231 Department of Medical Genetics, University Medical Centre Utrecht, Universiteitsweg, Utrecht, The Netherlands; 232 Department of Mental Health and Substance Abuse Services; National Institute for Health and Welfare, Helsinki, Finland; 233 Berkshire Healthcare NHS Foundation Trust, Bracknell, UK; 234 Section of Psychiatry, University of Verona, Verona, Italy; 235 Molecular and Cellular Therapeutics, Royal College of Surgeons in Ireland, Dublin, Ireland; 236 Health Research Board, Dublin, Ireland; 237 Departments of Psychiatry, Neurology, Neuroscience and Institute of Genetic Medicine, Johns Hopkins School of Medicine, Baltimore, Maryland USA; 238 Department of Clinical Medicine, University of Copenhagen, Copenhagen, Denmark; 239 Computational Sciences Center of Emphasis, Pfizer Global Research and Development, Cambridge, MA USA; 240 Department of Psychiatry, Georgetown University School of Medicine, Washington DC, USA; 241 Department of Psychiatry, Keck School of Medicine of the University of Southern California, Los Angeles, California, USA; 242 Department of Psychiatry, Virginia Commonwealth University School of Medicine, Richmond, Virginia USA; 243 Mental Health Service Line, Washington VA Medical Center, Washington DC, USA; 244 Medical Research Council Centre for Neuropsychiatric Genetics and Genomics, Division of Psychological Medicine and Clinical Neurosciences, Cardiff University, Cardiff, UK; 245 Centre for Population Health Research, School of Health Sciences and Sansom Institute of Health Research, University of South Australia, Adelaide, Australia; 246 UMC Utrecht Hersencentrum Rudolf Magnus, Utrecht, The Netherlands; 247 Institute for Molecular Bioscience, The University of Queensland, Brisbane, Australia

**Keywords:** Genetics research, Genome-wide association studies, Medical genomics, Functional clustering

## Abstract

Stratified medicine holds great promise to tailor treatment to the needs of individual patients. While genetics holds great potential to aid patient stratification, it remains a major challenge to operationalize complex genetic risk factor profiles to deconstruct clinical heterogeneity. Contemporary approaches to this problem rely on polygenic risk scores (PRS), which provide only limited clinical utility and lack a clear biological foundation. To overcome these limitations, we develop the CASTom-iGEx approach to stratify individuals based on the aggregated impact of their genetic risk factor profiles on tissue specific gene expression levels. The paradigmatic application of this approach to coronary artery disease or schizophrenia patient cohorts identified diverse strata or biotypes. These biotypes are characterized by distinct endophenotype profiles as well as clinical parameters and are fundamentally distinct from PRS based groupings. In stark contrast to the latter, the CASTom-iGEx strategy discovers biologically meaningful and clinically actionable patient subgroups, where complex genetic liabilities are not randomly distributed across individuals but rather converge onto distinct disease relevant biological processes. These results support the notion of different patient biotypes characterized by partially distinct pathomechanisms. Thus, the universally applicable approach presented here has the potential to constitute an important component of future personalized medicine paradigms.

## Abstract (web summary)

Stratified medicine promises to tailor treatment for individual patients, however it remains a major challenge to leverage genetic risk data to aid patient stratification. Here the authors introduce an approach to stratify individuals based on the aggregated impact of their genetic risk factor profiles on tissue-specific gene expression levels, and highlight its ability to identify biologically meaningful and clinically actionable patient subgroups, supporting the notion of different patient ‘biotypes’ characterized by partially distinct disease mechanisms.

## Introduction

Complex diseases affect millions of people each year and are responsible for ~70% of global deaths^1^. They originate from the complex interplay of genetic and environmental factors with varying contributions of the former. Understanding their molecular basis remains one of the major challenges of contemporary medical research^2,3^. Genome-wide association studies (GWAS) have exploited their frequently high heritability and identified hundreds of disease susceptibility loci across a wide spectrum of diseases^4,5^. However, it remains challenging to translate these associations into insights on molecular pathomechanisms. These challenges are rooted in the highly polygenic nature of these diseases, where individual associated genetic variants carry only small effect sizes^6^ and are mostly located in the non-coding space of the genome with unknown function^7,8^. Most importantly, a high level of heterogeneity in symptoms, disease course, and treatment response is severely impeding effective care for large numbers of affected individuals. This widespread heterogeneity on the clinical level coincides with a high level of genetic heterogeneity, where each patient harbors almost a private combination of disease-relevant genetic factors^9^.

These observations raise the question whether or not clinical heterogeneity at least partially originates from differences in the underlying genetic susceptibility^9^, giving rise to distinct underlying patient classes or biotypes, that are at present considered as homogeneous group on the clinical level^10^.

However, addressing this question is currently precluded by a critical gap between our insights into the *overall* disease association of individual genetic variants and the aggregated impact of these variants on biological processes and clinically relevant parameters *in individual patients*. This gap constitutes one of the major obstacles on the road towards the implementation of personalized medicine and the operationalization of genetic information in clinical decision making^11^.

A key step towards translating genetic associations into molecular biological consequences has been the development of transcriptome-wide association studies (TWAS). This approach combines genotype-based prediction of individual and tissue specific gene expression levels based on common variants with disease association testing^12,13^, enabling improved biological interpretability. In parallel, distinct types of polygenic score (PGS) and polygenic risk score (PRS) concepts were developed to resolve genetic heterogeneity and identify individuals at higher risk for a particular diagnosis or trait expression^14^. This stratification approach provides increased detection power to discover associations between different types of PGS and intermediate phenotypes or clinically relevant endpoints^15–17^ such as disease severity^18^. Conversely, patient stratification on the clinical and endophenotype level found ample evidence for distinct clinical subgroups, such as e.g. in heart failure^19^, type 1 diabetes^20^ or MDD and suggested distinct PRS levels of these phenotypically defined groups^10^.

However, alternative stratification approaches based on genetic correlations did not detect the presence of specific subgroups in most analyzed traits such as SCZ, MDD or diabetes^21,22^.

In summary, current approaches to genotype-based patient stratification rely on univariate genetic scores for a priori defined traits or specific hypothesis driven genes^23^/pathways^16,24^, resulting in a dichotomous classification of patients. These strategies are supervised in nature and require detailed a priori insights on potential disease mechanisms, precluding an unbiased discovery of subgroups and potential group-specific genetic liabilities. Moreover, traditional PGS approaches are agnostic of the underlying biological mechanisms, rendering the biological interpretation of resulting patient strata challenging.

To overcome these limitations, we sought to operationalize personal genetic profiles to stratify patients into biologically meaningful distinct subgroups in an unbiased and unsupervised manner and answer the question: How does heterogeneity in genetic risk factor distribution contribute to heterogeneity in clinical parameters and severity across patient populations?

To address this question, we develop here the CASTom-iGEx framework (Supplementary Fig. 1) to stratify patients into distinct subgroups based on tissue specific imputed gene expression and pathway activity profiles. When then applied this multivariate stratification strategy to different complex diseases (coronary artery disease or schizophrenia), identifying distinct patient subgroups that cannot be discovered by traditional PRS based analysis. We show that these groups are clinically meaningful and differ with respect to intermediate phenotypes, and clinical outcome parameters. Most importantly, we leverage the concept of a pathway level association studies to show that these groups differ with respect to the distribution of genetic disease liability across specific biological processes that are closely linked to their differences in intermediate phenotypes.

## Results

We predicted tissue specific gene expression profiles from individual level genotype data based on biologically meaningful sets of common variants using a modified elastic-net based method (PriLer, Methods, Supplementary Fig. 2). We trained this method on reference datasets from GTEx v6p^25^ and the CommonMind Consortium^26^, for a total of 34 tissues (Supplementary Table 1). While PriLer showed prediction performance comparable to the most popular existing approaches (FUSION, prediXcan, EpiXcan^27^, Supplementary Fig. 3a, b), it selected SNPs with a higher likelihood of being biologically meaningful based on overlap with various functional genomic annotations (Supplementary Fig. 3c, Supplementary Text). We, therefore, employed PriLer in subsequent analyses. However, due to the modular setup of CASTom-iGEx, all other analysis steps can also be performed with any other gene expression imputation method (see Discussion).

We next set out to test the hypothesis whether imputed gene expression profiles can be operationalized to resolve genetic and clinical heterogeneity across patients affected by complex diseases. To that end, we initially focus the paradigmatic application of this approach on coronary artery disease (CAD), a highly polygenic and clinically well-characterized disease caused by the buildup of plaques in the artery walls supplying blood to the heart.

### Unsupervised patient subgroup identification

We first applied GTEx trained PriLer models to predicted tissue-specific gene expression profiles for 11 tissues on 340,939 individuals from the UK biobank (UKBB) as well as 9 independent CARDIoGRAM^28^ cohorts to assess reproducibility (*n* = 26,681). To enable CAD patient subgroup discovery, we next implemented an unsupervised clustering strategy of predicted patient-level gene expression profiles. To that end, we first transformed the patient-level imputed gene expression values to T-scores for each gene and tissue. The latter quantify the deviation of gene expression in each patient relative to a reference population of healthy individuals. This transformation ensures a similar distribution of expression values across samples for each gene (Supplementary Fig. 4a, b), with the gene variance being not dependent on the PriLer model performances (Supplementary Fig. 4c) and reducing the correlation among samples (Supplementary Fig. 4d, Supplementary Text).

In order obtain patient subgroup definitions that are related to the disease phenotype, we weighted the contribution of each gene in the clustering according to its relevance for the overall CAD phenotype. We therefore performed tissue specific transcriptome-wide association analyzes (TWAS) based on the individual-level gene T-score profiles, giving rise to standard phenotype-association statistics quantified as Z-statistic (Supplementary Fig. 5a, Supplementary Data 1). Subsequently, the individual level gene T-scores were weighted by the CAD gene Z-statistics to derive a weighted individual-level gene expression value, incorporating disease association strength. These scores were then used in an unsupervised clustering based on Leiden clustering for community detection^29^, partitioning CAD patients into distinct subgroups, using empirically optimized hyperparameters (Methods, Supplementary Fig. 5b–h).

Clustering was performed for each tissue separately, correcting for ancestry contribution as well as other covariates, almost eliminating the impact of these confounders while maintaining a robust clustering structure (Supplementary Fig. 5i, j, Methods). Importantly, the strategy to weigh T-scores with the CAD phenotype association Z-statistics proved crucial to achieve well-defined clusters (Supplementary Fig. 6a, b) and allows genes/pathways that are more relevant for CAD to have a higher impact in the final clustering configuration (Supplementary Fig. 6c, d, see also Supplementary Text).

**Fig. 1:**
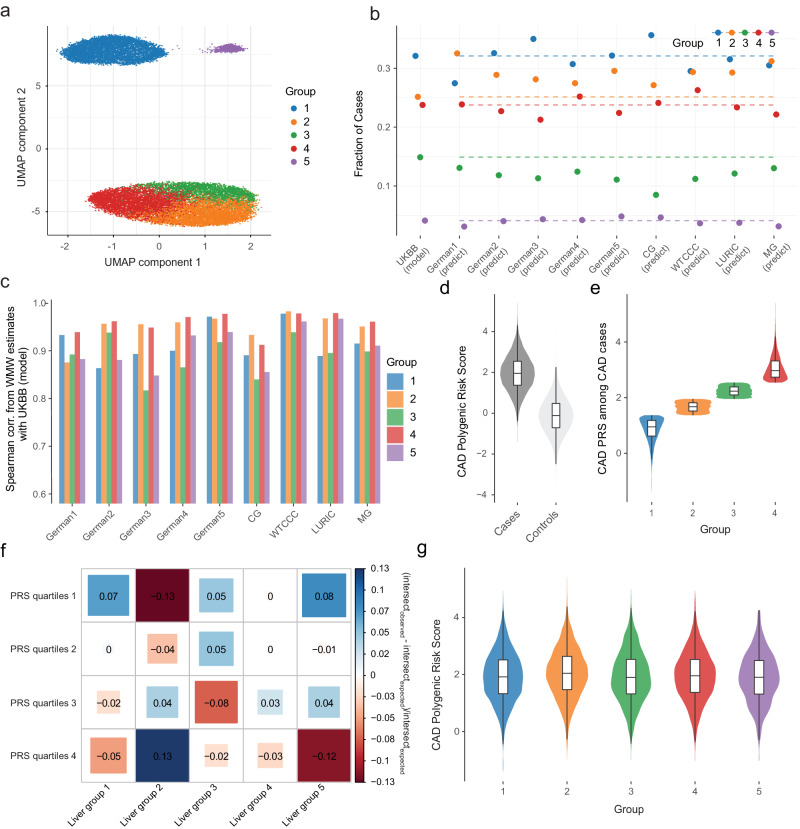
Stratification of CAD patients from imputed gene expression. **a** First 2 components of uniform manifold approximation and projection (UMAP) from gene T-scores in liver for CAD patients. Genes are clumped at 0.9 correlation, separately standardized and PCs corrected, and multiplied by Z-statistic CAD associations. Each dot represents a patient colored by the cluster membership. **b** Prediction of clustering structure on 9 external CARDIoGRAM cohorts. Y-axis shows the fraction of cases assigned to each cluster in UKBB dataset and each external cohort for which the clustering structure was projected. The dashed lines indicate the fraction value for UKBB model clustering. **c** For each group, Spearman correlation of WMW estimates in UKBB and each external cohort only from genes that are significantly associated with that group across all tissues. **d** Distribution of CAD polygenic risk score (PRS) for all UKBB individuals based on CAD GWAS summary statistics from UKBB CAD GWAS. Cases: 19,023, controls: 321,916. The quartiles represent the 25th, 50th (median), and 75th percentiles. Upper whiskers extend to the maximum data value within 1.5 times the interquartile range (IQR) above the 75th percentile, while lower whiskers reach the minimum data value within 1.5 times the IQR below the 25th percentile. Violin plots encompass both the maximum and minimum values. **e** Distribution of CAD PRS for CAD affected individuals split into 4 groups based on PRS quantiles from lowest (1) to highest (4) PRS values. N. of samples in each group is respectively gr1 4756, gr2 4756, gr3 4755 and gr4 4756. Boxplots and violin plots show the same statistics as (**d**).**f** Enrichment between PRS quartiles and liver partitions. Each value indicates the fraction of (observed - expected)/expected individuals in the intersection between the groups as computed from the chi-squared statistic. Color and shape reflect the extent of enrichment. **g** Distribution of CAD PRS across CAD-affected individuals for groups defined by CASTom-iGEx clustering. N. of samples in each group is respectively gr1 6105, gr2 4783, gr3 2831, gr4 4520, gr5 784. Boxplots and violin plots show the same statistics as (**d**).

Overall, unsupervised clustering analysis of *n* = 19,026 CAD patients from the UKBB identified between 3 and 10 groups of CAD patients (Supplementary Fig. 6e) that largely overlap for clustering results from different tissues (Supplementary Fig. 6f). In light of these similarities among clustering results and the relevance of liver in CAD pathophysiology, we focused on patient stratification based on expression profiles in the liver (Fig. 1a). Careful evaluation of this patient group structure revealed that the latter was not driven by single genes, but rather by a combination of CAD associated genes from multiple independent loci (Supplementary Fig. 6g, Supplementary Data 2). Moreover, analysis of well-known confounding factors showed that group structure was not driven by age, sex or ancestry contributions (see the detailed analysis of the latter factors in Supplementary Text and Supplementary Fig. 7a–d, Supplementary Fig. 8).

To evaluate the generalizability and reproducibility of this patient stratification approach, we projected the imputed gene level score profiles from 9 independent CARDIoGRAM cohorts (*n* = 13,279 CAD patients) onto the clustering structure discovered on the UKBB dataset (see Methods). Subsequently, we determined the fraction of CARDIoGRAM CAD cases assigned to each cluster. This analysis revealed a virtually identical distribution of CAD cases across the clusters compared to the original UKBB dataset (Fig. 1b). Spearman correlation analysis of the cluster-specific genes expression estimates (Methods) for individual CARDIoGRAM cohorts and the UKBB dataset showed excellent concordance (cor. > 0.8, Fig. 1c), with WTCCC being the most consistent cohort (cor. > 0.9) and was not driven by a single locus (Supplementary Fig. 7e).

Jointly, these results establish the CASTom-iGEx stratification scheme as a reproducible and unbiased approach to derive genetically defined patient groups in an unsupervised manner.

### Comparison of CASTom-iGEx and PRS based stratification

To assess the added value of this stratification strategy, we compared the CASTom-iGEx derived patient subgroups to the current state-of-the-art stratification strategy based on PRS profiles. To this end, we partitioned all CAD cases into 4 equally sized groups based on their CAD PRS quartiles derived from a GWAS that we conducted on the UKBB dataset (Fig. 1d, e, Methods). The resulting PRS grouping of CAD patients was highly distinct from the CASTom-iGEx based clustering, with minimal overlap (NMI = 0.0013, Fig. 1f) and PRS being equally distributed across the CASTom-iGEx clusters (Fig. 1g).

Next, we evaluated whether the clustering structures were able to resolve clinical heterogeneity across the CAD patient population and tested 249 disease relevant endophenotypes and clinical parameters for subgroup specific association with respect to all other patients.

**Fig. 2:**
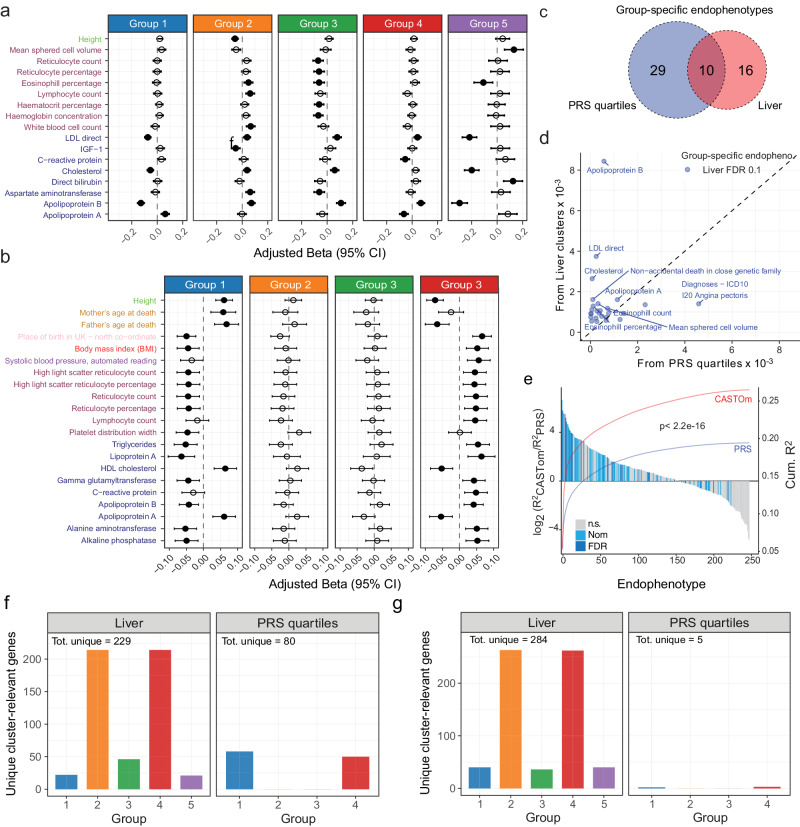
CASTom-iGEx based stratification outperforms PRS grouping. **a** CAD relevant continuous endophenotypes from the UKBB with significant (FDR ≤ 0.1) patient group specific differences compared to all remaining CAD patients based on CASTom-iGEx groups depicting regression coefficient (β_GLM_) with 95% Confidence Interval (CI). Full dot indicates that β_GLM_ is significant (0.1 threshold) after BH correction. Similar results for binary and ordinal categorical phenotypes are shown in Supplementary Fig.  9a. N. of samples in each group is are gr1 = 6105, gr2 = 4783, gr3 = 2831, gr4 = 4520, gr5 = 784. For each endophenotype tested, the number of samples per group varied and was lower than the entire CAD case population of 19,023 due to missing values, ranging from 16314 to 18919 total cases. **b** Similar to a. for PRS quantile-based CAD patient grouping (FDR ≤ 0.1). N. of samples in each group is are gr1 = 4756, gr2 = 4756, gr3 = 4755 and gr4 = 4756. Forest plot measures are defined as in (**a**). **c** Overlap of unique significantly CAD patient group associated endophenotypes for PRS quantile (blue) and CASTom-iGEx (red) based grouping. **d** For group-specific endophenotypes in liver clustering (FDR ≤ 0.1), comparison between the variance explained (R^2^) by liver partition (y-axis) and PRS quartiles partition as computed from the difference of R^2^ in the full linear model (pheno ~ group + cov) and the covariates only model (pheno ~ cov). **e** For all CAD related endophenotypes (*n* = 249, x-axis) log_2_ ratio of variance explained (R^2^) between the CASTom liver patient strata and PRS quartile patient strata (y-axis left). Each bar represents one endophenotype, color coding indicates significance of endophenotype-patient stratum association (n.s. – not significant, nom – nominally significant p-value ≤ 0.001, FDR – FDR ≤ 0.1). Lines show cumulated variance explained (y-axis left) across all endophenotypes for CASTom liver-based grouping (red) and PRS quartiles (blue). P-value indicates difference in cumulated variance based on Wilcoxon-test. **f** Number of unique genes across tissues cluster-relevant (FDR ≤ 0.01) divided per group, in CASTom-iGEx liver (left) and PRS quartiles (right) partitions. The total number across all groups of cluster-relevant genes is shown on top. **g** Same as f. but for cluster-relevant pathways (FDR ≤ 0.01).

The CASTom-iGEx based clustering resulted in 42 significant cluster specific endophenotype associations (26 unique endophenotypes), all with high disease relevance (FDR ≤ 0.1, Fig. 2a, Supplementary Fig. 9a, Supplementary Data 3). Similarly, the PRS based patient stratification did also result in the identification of 64 CAD relevant endophenotypic differences (39 unique) (Fig. 2b, Supplementary Fig. 9b), with 10 (18.1%) associations being detected in both approaches (Fig. 2c).

These results reproduced the known association of hypertension, diabetes and increased triglycerides with a high PRS^30^. For the CASTom-iGEx based clustering, differences in endophenotype profiles were more diverse, with distinct configurations of high/low endophenotype values (Fig. 2a) compared to the PRS clustering. For the latter, consistently high or low endophenotype profiles were limited to high or low PRS groups (Fig. 2b). Moreover, CASTom-iGEx derived patient clusters explained consistently more variance across the vast majority of CAD-related endophenotypes compared to the PRS based grouping (Fig. 2d,e 62% of all and 81% of significantly group associated endophenotypes). For several key CAD-related endophenotypes such as APOB, LDL-cholesterol or total cholesterol the variance explained by the CASTom-iGEx based clustering increased between 2-5 fold (Fig. 2e).

### Distinct biological basis of CASTom-iGEx but not PRS derived patient strata

Next, we tested the hypothesis that PRS and CASTom-iGEx defined patient groups were characterized by distinct genetic liabilities across disease relevant biological processes.

To this end, we determined the differences in imputed gene expression profiles between all groups within each clustering scheme, after confirming the well-calibrated nature of the gene association statistic (see Methods and Supplementary Text, Supplementary Fig. 10). This analysis identified a total of 229 and 80 unique genes with differential activity patterns across the CASTom-iGEx and PRS based clustering respectively. For the former, each group exhibited between 21 and 214 differentially active genes in each group, while the PRS groups showed ~50 differentially active genes between the low and the high PRS strata (Fig. 2f).

To enable the discovery of biological processes perturbed by the group specific genetic liabilities, we devised a strategy to aggregate the individual weak genetic effects of common variants beyond the gene to the pathway level. This methodology relies on the aggregation of gene-level scores into continuous pathway activity scores at the individual level, using a predefined set of pathways from GO biological processes^31^, Reactome^32^ and WikiPathways^33^ (see Methods). Similar to the imputed gene expression levels, these pathway activity scores can be used for pathway level association studies (PALAS) for case/control comparisons or the discovery of group-specific pathway associations (see Methods, Supplementary Data 4).

Prior to proceeding with the group-specific evaluation, we first confirmed the well-calibrated nature of this approach on permuted data from CAD (Supplementary Fig. 11a-d), external replication cohorts (Supplementary Fig. 9e) and group specific pathway association testing (Supplementary Fig. 11f-g). Importantly, the PALAS methodology detected substantially more CAD associated pathways compared to more traditional pathway enrichment strategies such as hypergeometric testing of TWAS significant genes or MAGMA^34^ that each rely on summary statistics (Supplementary Fig. 12a,b). This increase in detection power partially resulted from aggregation over weak association effects (Supplementary Fig. 12c,d) and was not driven by genetic correlation due to LD structure (Supplementary Fig. 12e-i, Supplementary Text).

Application of this approach to discover differences in biological process activity levels across CASTom-iGEx and PRS derived patient strata identified a total of 284 and 5 unique associated pathways respectively (Fig. 2g). While all CASTom-iGEx based patient groups showed at least 36 pathway level associations, only PRS-based groups low and high-risk showed 2 or 3 associations respectively (Fig. 2g).

Jointly, these results show that a PRS-based stratification detects clinically relevant subgroups that exhibit differences in many disease-relevant clinical parameters and endophenotypes between the low and the high-risk group. However, the latter grouping lacks a common biological basis. Instead, reduced/elevated genetic liability is mostly randomly distributed across genes and pathways in the respective PRS groups. Similarly, clustering of randomly selected individuals from the UKBB showed minimal overlap in detected endophenotypic differences between clusters (Supplementary Fig. 13a-e, Supplementary Text) or minimal overlap of the overall group structure if no information on CAD relevance of genes used (Supplementary Fig. 13f-i).

In contrast, patient strata derived through the CASTom-iGEx approach exhibit a non-random distribution of genetic liability across genes in each patient. In particular, the CASTom-iGEx analysis shows that aggregation of genetic liability across specific biological processes is distinct in different patients. These genetically defined patient strata exhibit a divergence in their disease relevant clinical and physiological parameters, suggesting potentially group specific pathomechanisms constituting distinct patient biotypes.

### CASTom-iGEx derived patient strata resolve genetic heterogeneity across CAD associated biological processes

**Fig. 3:**
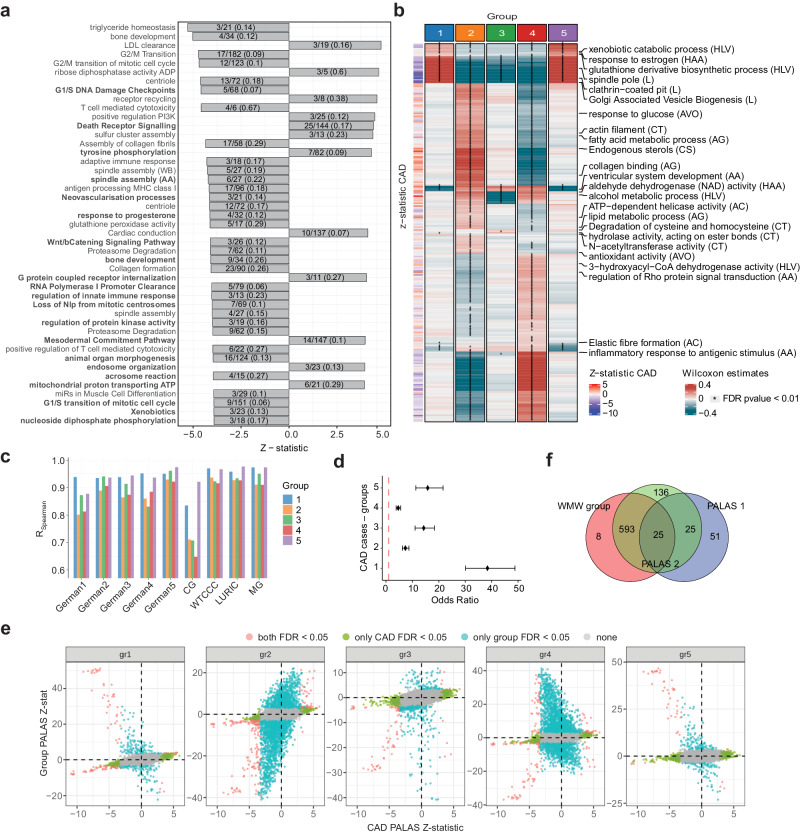
Differences in genetic liabilities across distinct biological process across CAD patient groups. **a** CAD associated pathways with higher significance than any of the included genes. Bars indicate PALAS Z-statistic (x-axis) with text signifying gene pathway coverage. The pathway name in bold reflects pathways without any significant gene (FDR > 0.05). **b** Pathways significantly (FDR ≤ 0.01) differentially active across CAD patient groups based on Wilcoxon-Mann-Whithney (WMW) analysis (test two-sided). Rows indicate the names of selected pathways and respective tissue is shown in parentheses. The left-side annotations show the corresponding CAD Z-statistics from PALAS 1. **c** Spearman correlation of WMW estimates of pathway scores between all significant group-specific pathways in UKBB (y-axis) and the corresponding pathways in each external cohort (CARDIoGRAM) (x-axis) for each group (color coding) across all tissues. **d** Odds ratio (median-unbiased estimation) with 95% CI of PALAS cluster pathways among PALAS CAD pathways (FDR ≤ 0.05). PALAS cluster pathways are detected from PALAS comparing non-affected individuals with CAD cases in each group from Liver. In each group, the number of pathways both in negative classes (PALAS cluster FDR > 0.05 and PALAS CAD FDR > 0.05) and both in positive classes (PALAS cluster FDR ≤ 0.05 and PALAS CAD FDR ≤ 0.05) are respectively gr1: negative 36140, positive 116; gr2: negative 33272, positive 231; gr3: negative 35962, positive 81; gr4: negative 33405, positive 166; gr5: negative 36165, positive: 49. **e** Comparison z-statistic for general CAD PALAS (PALAS 1, x-axis) and patient group specific PALAS (PALAS 2, y-axis) for each CASTom-iGEx defined group. Red dots indicate significant (FDR ≤ 0.05) associations in both PALAS, green significance only in PALAS 1 and turquoise significance only in PALAS 2. **f** Overlap of pathways significantly (FDR ≤ 0.05) associated with CAD (blue, PALAS 1), significantly associated with at least one CASTom-iGEx based patient group compared to all controls (green, PALAS 2), and those showing group specific activities when compared to all other CAD cases only (red, WMW group) out of 7978 tested pathways retained after pathway similarity pruning (JS < 0.2, see Methods).

In order to determine the shared and group specific pathway activity profiles that discriminate patient groups from healthy controls, we performed three pathway-related analysis. First, we determined all genes and pathways associated with the entire population of CAD patients compared to *n* = 321,831 controls and identified 567 significant pathways (FDR ≤ 0.05, PALAS 1, Fig. 3a, Supplementary Fig. 12a, Supplementary Data 4). We then performed the same PALAS analysis for each patient group separately relative to the entire unaffected control population, sacrificing detection power but decreasing heterogeneity (PALAS 2, identifying 4058 unique pathways associated with at least one group FDR ≤ 0.05). Finally, we tested for cluster-specific pathways discriminating patient groups from each other, testing each group versus all other CAD cases (WMW group analysis), identifying 626 unique pathways (Wilcoxon-Mann-Whitney test, FDR ≤ 0.01) (Fig. 3b, Supplementary Data 5). Replication of the group specific results in a secondary analysis using genotype data from 9 independent CARDIoGRAM cohorts (*n* = 13,279 cases) confirmed a high level of replicability of patient group specific pathways scores (Fig. 3c, Supplementary Fig. 14a). Moreover, validation of group-specific imputed gene expression and pathway scores on non-imputed data using an independent population-based cohort with genotyping as well as transcriptome data from whole blood (SHIP-TREND^35^, *n* = 976) was also in good agreement with the predictions on the UKBB (Supplementary Fig. 14b-g, Supplementary Text).

Subsequently, we compared the results of all three pathway-related analyzes. As expected, pathways associated with each individual group were highly enriched in the pathway set associated with the union of all CAD patients compared to healthy controls (PALAS 1, median unbiased estimator P < 1e-37, Fig. 3d). Next, we sought to discriminate between those pathways associated with CAD across the union of all clusters (PALAS 1) and those only associated with individual CAD patient groups (PALAS 2, WMW group). To this end, we first filtered (Methods) and decomposed the general set of CAD associated pathways (PALAS 1, *n* = 467) according to the sign concordance of their association statistic. 264 (57%) of pathways showed the same sign across all groups and PALAS 1, although not always reaching significance in each subgroup PALAS (Supplementary Fig. 9c). These pathways are indicative of shared pathomechanisms across patient groups, and included e.g. apolipoprotein binding, death receptor signaling and cyclin−dependent protein serine/threonine kinase inhibitor activity (Supplementary Fig. 9c). The remaining 197 CAD pathways (43%) exhibit a discordant sign of association in at least one group, indicating cluster-specific mechanisms and include pathways such as Golgi Associated Vesicle Biogenesis, antigen processing and presentation and actin filament (Supplementary Fig. 9d).

Considering all the available pathways, the majority of general CAD hits (PALAS 1) showed evidence of association with most individual patient groups (Fig. 3e). In contrast, only 6.3% of pathways identified in the group-specific analyzes and evaluated in all three pathway analyzes (PALAS 2 and WMW group) were also detected in the general CAD PALAS 1(Fig. 3f), with most group-specific associations showing only weak general CAD signal (Fig. 3e). These genes and pathways suggest the presence of patient group specific genetic liabilities in additional biological processes.

These results underscore the presence of distinct genetic liabilities towards different biological processes in different groups. Moreover, they highlight the capacity of CASTom-iGEx to deconstructs genetic heterogeneity across CAD associated biological processes.

### CASTom-iGEx patient stratum specific de-regulated pathways directly modulate patient group associated endophenotypes

These previous analyzes establish (1) the existence of distinct CAD patient subgroups, which are characterized by (2) partially distinct genetic liabilities across biological processes as well as (3) group-specific differences in disease-related endophenotypes.

However, it is unclear, whether these differences in endophenotypes and clinical parameters are *linked* to the group-specific differences in genetic liability profiles across biological processes. To test this hypothesis, we evaluated whether or not pathways with significant group-specific activity profiles (Fig. 3c, d) contribute the modulation of the respective group specific endophenotype profiles.

**Fig. 4:**
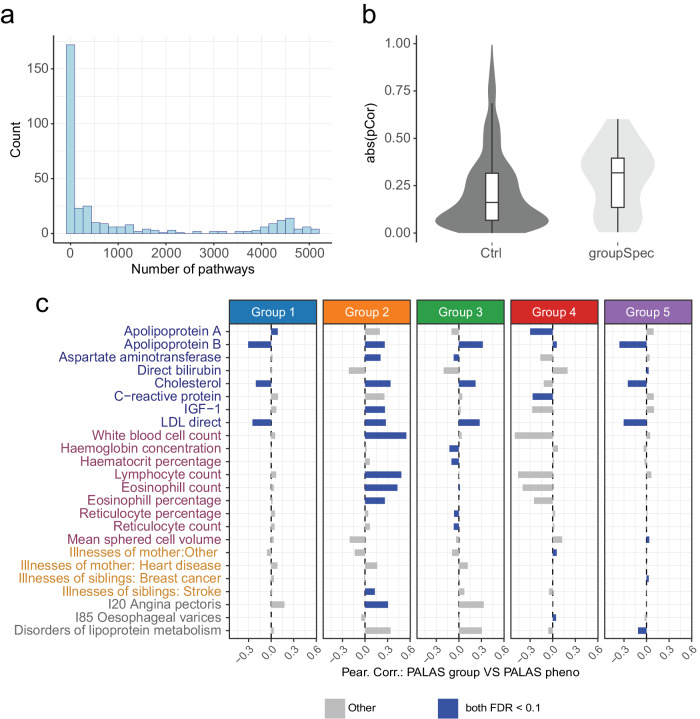
Patient group-specific genetic liabilities are linked to the genetic basis of group-specific disease relevant endophenotypes. **a** Frequency of pathway number (x-axis) significantly (FDR ≤ 0.1) associated with UKBB endophenotypes (*n* = 341). **b** Distribution of absolute Pearson correlation (y-axis) of significant (FDR ≤ 0.1) pathway-endophenotype and pathway-patient group association PALAS z-statistic for control endophenotypes (*n* = 317) and CAD patient group associated endophenotypes (*n* = 24). The quartiles illustrated in box plots represent the 25th, 50th (median), and 75th percentiles. The interquartile range (IQR) denotes the difference between the 75th and 25th percentiles. Upper whiskers extend to the maximum data value within 1.5 times the IQR above the 75th percentile, while lower whiskers reach the minimum data value within 1.5 times the IQR below the 25th percentile. Violin plots encompass both the maximum and minimum values. **c** Forest plot showing Pearson correlation (x-axis) between pathway z-statistic for CAD patient group specific PALAS and z-statistic for pathways associated with UKBB endophenotypes (y-axis) for each CASTom-iGEx defined group. Only endophenotypes significantly associated with at least one group (FDR ≤ 0.1) are considered. Blue bar indicates that the association is significant in both measured group-specific endophenotype and correlation from group PALAS and endophenotype PALAS z-statistics (both FDR ≤ 0.1).

Therefore, we determined the bona fide genetic basis of CAD relevant endophenotypes and linked them to specific biological processes. We performed individual PALAS for all patient group associated endophenotypes (*n* = 26) as well as a large set of control endophenotypes (*n* = 317) across the entire UKBB population, irrespective of diagnosis status. This analysis identified between 0 and 5,123 significant (FDR ≤ 0.1) pathway-endophenotype associations (Fig. 4a). Comparison of pathway-endophenotype (Supplementary Data 6) and pathway-patient group association statistic (Z-statistic) revealed a strong correlation for group associated endophenotypes and an overall weak correlation for not group associated endophenotypes (Fig. 4b). Importantly, almost all significantly CAD group associated endophenotypes showed high group specific correlation between the respective endophenotype and patient stratum specific pathway scores (Fig. 4c).

Jointly, these results show that differences in disease-relevant endophenotypes across CAD patient groups are linked to genetically driven differences in the biological processes underlying these endophenotypes.

### Identification of clinically relevant subgroups in CAD with distinct genetic liability in disease related biological processes

**Fig. 5:**
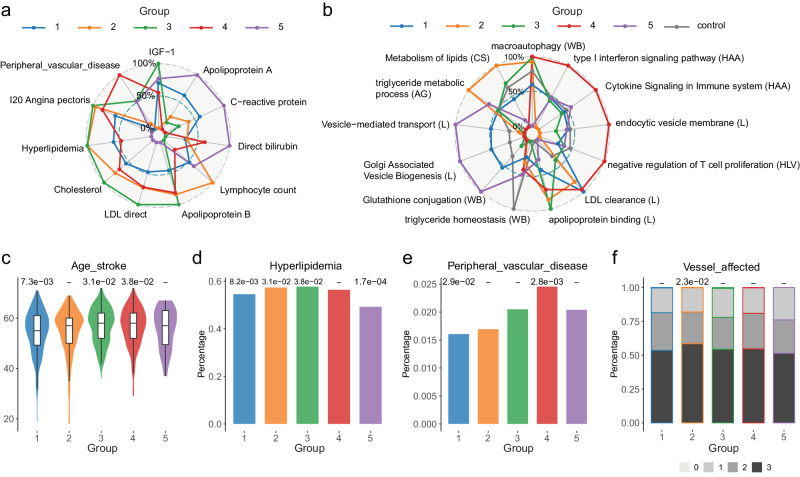
Distinct CAD patient groups exhibit differences in clinical outcome parameters. **a** Mean value of selected group-specific endophenotypes in each group rescaled to 0-100 range. **b** Mean pathway score value of selected group-specific pathways compared to healthy controls. The values are rescaled to 0-100 range and include the average scores for controls as reference. **c** Distribution of age of stroke for patients in UKBB. In c-e nominal p-values from group-wise GLM is shown at the top of the bar/violin plot. Boxplot elements include median as central line, 1^st^ and 3^rd^ quartiles as box limits, 1.5 interquartile ranges from 1st and 3rd quartiles as corresponding whiskers. N. of samples in each violin/boxplots are respectively:gr1 = 294, gr2 = 242, gr3 = 142, gr4 = 235, gr5 = 35. **d** Percentage of patients in UKBB clustering with comorbidity hyperlipidemia. **e** Percentage of patients in UKBB clustering with peripheral vascular disease. In (h-k). **f** CAD severity indicators across projected clusters in GerMIFSV cohort. Y-axis indicates the percentage of patients with a certain number of vessels affected (gray shades). X-axis indicates the projected group.

Subsequently, we jointly evaluated the group-specific cluster endophenotype and pathway associations (Figs. 2a, 5a, b) to obtain insights into any potential group-specific pathomechanisms. In this context, we only considered group specific pathways (PALAS 2, FDR ≤ 0.1) that were also significantly associated with the respective endophenotype (FDR ≤ 0.1).

CAD group 1 is characterized by significantly lower LDL-direct, cholesterol and apolipoprotein B (APOB) levels compared to all other groups (Fig. 5a) as well as elevated apolipoprotein A (APOA). This endophenotypic profile is accompanied by a significant increase in predicted pathway activity of LDL clearance and vesicle-mediated transport pathways. Conversely, group 1 exhibits a significant decrease in apolipoprotein binding, triglyceride homeostasis and macroautophagy related pathways, known to modulate e.g. apolipoprotein levels^36^ (Fig. 5b).

In contrast, CAD group 2 shows a reduction in Golgi Associated Vesicle Biogenesis, vesicle-mediated transport, ABC transporter related genes and endocytosis (Fig. 5b). This pathway activity reduction is accompanied by a significant increase of circulating LDL-cholesterol, total cholesterol and APBOB levels on the patient endophenotype level (Figs. 2a, 5a). The latter is consistent with the notion that vesicles filled with LDL-cholesterol particles are taken up by the cells via receptor-mediated endocytosis mechanisms^37^. Accordingly, circulating LDL levels exhibit a strong genetic association with endocytosis-related pathways. Similarly, group 2 shows a significant increase in fatty acid and general lipid metabolic processes that is also significantly associated with circulating LDL levels, consistent with overall higher LDL- and total cholesterol levels in patients of group 2 (Figs. 2a, 5a). Lastly, patients in group 2 exhibit an increase in immune cell populations, concomitant with a predicted increase in genes related to T cell proliferation as well as a decrease in IGF1 levels and cytokine signaling (Fig. 5b).

To evaluate the potential clinical relevance of these observations, we next assessed whether these differences in liabilities across genes, biological processes and endophenotypes were associated with differences in clinical parameters such as disease severity and/or trajectory (Supplementary Data 7). To this end we leveraged additional clinical phenotypes collected on 2383 CAD patients (GerMIFSV in CARDIoGRAM), evaluated between patient groups following their projection onto the UKBB clustering as well as 33 clinical parameters collected in UKBB.

This analysis revealed that patients of group 1 show a significantly lower age of stroke (Fig. 5c) as well as a lower incidence rate of hyperlipidemia and peripheral vascular disease (Fig. 5d, e). In contrast, patients in group 2 in GerMIFSV have a significantly higher number of vessels affected by CAD, indicative of a more severe disease course (Fig. 5f) and consistent with significantly lower IGF-1 levels^38^ compared to all other groups (Fig. 5a). Moreover, group 2 patients show a mid-range age of stroke as well as a higher incidence of hyperlipidemia (Fig. 5f) and angina pectoris (Fig. 2b). These observations are consistent with the overall higher levels of key CAD related endophenotypes (LDL, APOB, immune cell population) and elevated genetic liability towards the perturbation of associate endophenotype relevant pathways including lipid metabolism, and endocytosis related pathways. Thus, group 2 constitutes the clinically most severely affected patient group.

Similarly, patients in group 3 exhibit significantly increased LDL-cholesterol, total cholesterol and APOB levels but no significant change in APOA. In contrast, group 3 patients show significantly decreased white blood cell counts. On the pathway level, these endophenotype profiles were linked to significantly altered genetic liability towards lower endocytosis and lower Golgi vesicle biogenesis. Similar to group 2, group 3 patients also show an increased frequency of hyperlipidemia and age of stroke.

In contrast, group 5 shows the lowest levels CAD related endophenotype values, (Figs. 2a, 5a) as well as the lowest frequency of clinically relevant outcome parameters and other diseases, including significantly reduced frequency of hyperlipidemia (Fig. 5d–f). Group 5 also shows the highest levels of APOA and direct bilirubin levels (Figs. 2a, 5a), recently implied as a biomarker for long term outcome and disease severity in CAD^39^. These observations suggest that group 5 represents the healthiest group of CAD patients.

Simultaneously, group 5 exhibits the lowest genetic liabilities across CAD associated biological processes as well as significantly increased predicted endocytosis and Golgi associated vesicle biogenesis (Fig. 5b). Although not significant, we also observed a trend of increased CRP levels in group 5 (Fig. 2a) connected to endocytosis and glutathione biosynthesis liabilities. Interestingly, glucosamine consumption reduced CRP levels in group 5 individuals, compared to all the other groups, where no decrease or even an opposite trend was observed (Supplementary Fig. 15). This analysis suggests a possible cost-effective therapeutic strategy to decrease inflammatory activity for patients with precise genetic liabilities.

Finally, patients assigned to group 4 exhibit decreased levels of LDL-cholesterol, total cholesterol and APOA concomitant with decreased liabilities of biological processes of pathways linked to these endophenotypes. In contrast, patients in group 4 show increased genetic liability towards many immune-related pathways such including interferon signaling as well as response to insulin (Fig. 5b), all of which were negatively associated with markers of inflammatory processes such as C-reactive protein (CRP) (Supplementary Data 5). Consistent with this finding, group 4 patients show decreased CRP levels compared to all other groups (Fig. 2a) and an increased frequency of peripheral vascular diseases and a slightly higher age of stroke (Fig. 5c,e). These observations suggest an increased relevance of inflammation related processes in CAD specifically in this subgroup of patients that is linked to distinct clinical characteristics.

In summary, these analyzes show the existence of CAD patient strata in with distinct genetic liabilities across biological processes that are directly linked to differences in disease-relevant endophenotypes and clinical parameters.

### Deconstructing heterogeneity among SCZ patients

Going beyond well characterized CAD, we decided to evaluate the capacity of CASTom-iGEx to obtain insights into the biological basis of clinical heterogeneity in SCZ as a more enigmatic illness. While the existence of clinical subtypes of SCZ patients is well known^40^, it is at present unclear, whether or not this phenotypic heterogeneity might result from a distinct genetic basis and potentially distinct biological mechanisms. To address these questions, we applied the CASTom-iGEx pipeline to 36 European cohorts from Psychiatric Genomic Consortium (PGC) wave 2^41^ for a total of 24,764 cases and 30,655 controls, leveraging 9 GTEx tissues and DLPC (dorsolateral prefrontal cortex) gene expression data from the CommonMind consortium as PriLer gene expression model training data^26^ (Supplementary Data 8 & 9).

**Fig. 6:**
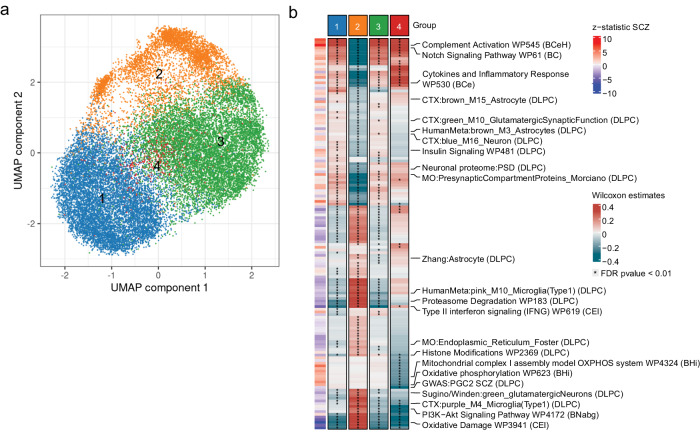
CASTOM-iGEx based identification of distinct patient subgroups in SCZ. **a** Uniform manifold approximation and projection (UMAP) first 2 components of gene T-scores in DLPC standardized across *n* = 24,764 SCZ patients, corrected for PCs, and multiplied by Z-statistic SCZ associations. Each dot represents a patient in the transformed UMAP space colored by the cluster membership. **b** Wilcoxon-Mann-Whitney (WMW) estimates (test two-sided) for 296 group-specific pathways (FDR ≤ 0.05, Reactome and GO) including at least one gene in the MHC locus and considering only the most significant tissue per-pathways when repeated. The clustering is performed on SCZ patients in DLPC imputed gene expression, The row annotation on the left indicates the corresponding SCZ PALAS Z-statistics. The acronym in parenthesis in the pathway names refers to the tissue considered (DLPC = Dorsolateral Prefrontal Cortex in CMC, CEI = Cells EBV-transformed lymphocytes, BFBC = Brain Frontal Cortex BA9, BCeH = Brain Cerebellar Hemisphere, BCbg = Brain Caudate basal ganglia, BC = Brain Cortex, BCe = Brain Cerebellum, BHi = Brain Hippocampus, BHy = Brain Hypothalamus).

Following a similar strategy as applied for CAD (Methods), we identified 4 groups of SCZ patients based on clustering of 5,682 gene T-scores from DLPC (Supplementary Fig. 16, Fig. 6a) on 35 PGC cohorts. Detailed analysis of potential confounders revealed minimal impact of ancestry and cohort membership on clustering structure as well as on detected gene associations (Supplementary Fig. 17a–d, Supplementary Fig. 18, Supplementary Text).

In total, we identified 755 cluster-specific genes (FDR ≤ 0.01) out of 26,836 tested across the 10 tissues distributed across 124 independent loci (Supplementary Fig. 17a, Supplementary Data 10). The reproducibility of the observed clustering structure and identified group specific genes in the left-out PGC cohort was high based on the distribution of patients across groups and spearman correlation ( > 0.95 for 3 groups and >0.65 for group 4) of groupwise gene-expression profiles and (Supplementary Fig. 17e, f).

Similarly, we identified 296 ( + 145 WikiPathway / CMC gene-set) unique pathways out of 6,120 ( + 2,865 WikiPathway / CMC gene-set) with differential liability profiles (Fig. 6b, Supplementary Fig. 17g, Supplementary Fig. 19, Supplementary Data 11). Given the absence of large deeply phenotyped cohorts for SCZ, we turned to a different strategy for the identification of groupwise differences in endophenotypes and interpretation of pathway level liability profiles based on endophenotype approximation via endophenotype risk-scores. Prior to application of SCZ, we carefully benchmarked this approach in CAD (see Methods, Supplementary Fig. 20).

**Fig. 7:**
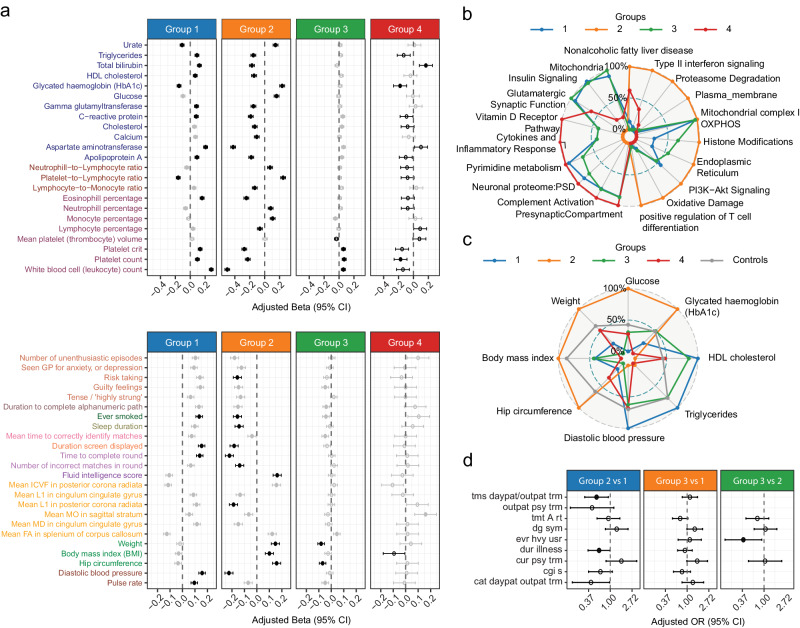
CASTom-iGEx defined SCZ patient groups differ with respect to cognitive parameters, risk for metabolic syndrome and disease severity. **a** Forest plot for selected significantly different (FDR ≤ 0.05) endophenotype risk-scores across SCZ patient groups. X-axis shows the regression coefficient (dot) with 95% CI for the grouping variable (β_GLM_). The bars represent CI computed as [β_GLM_ −1.96 * SE, β_GLM_ + 1.96 * SE]. Full dot indicates that β_GLM_ is significant after BH correction. Black dot indicates that the group-specific endophenotype association met the reliability threshold (CRM > 610, Methods). The top panel shows results for blood biochemistry, lower panels indicates other clinical and cognitive parameters. Endophenotypes are imputed here, the number of samples across them stays constant with gr1 = 9029, gr2 = 4418, gr3 = 8860 and gr4 = 520. **b** Rescaled mean values of selected SCZ patient group-specific pathways (Reactome and GO, WikiPathways and CMC Gene Set). **c** Group-specific spider plot related to Metabolic Syndrome phenotypes. Rescaled mean values of group-specific endophenotype-RS related to metabolic syndrome across all cohorts. Gray chart refers to all control combined in PGC cohorts. **d** Forest plot testing measured clinical differences across SCZ patients from the PsyCourse Study after individual patient projection onto PGC patient-based clusters. Forest plot as in a. with GLM testing for each pair of groups (label on top) and dots representing obtained odds ratio as exp(β_GLM_) being the endophenotypes binary / ordinal categorical. The bars represent CI computed as [exp(β_GLM_ −1.96 * SE), exp(β_GLM_ + 1.96 * SE)]. Full dot indicates significance at nominal level (p $\le 0.05$). tr. out/in – treatment outpatient/inpatient. Group sizes are gr1 = 75, gr2 = 237, gr3 = 244.

These analyzes resulted in 68 endophenotypes (out of 1000, see Methods) that differ reliably in at least one SCZ patient group (Fig. 7a, Supplementary Data 12). Jointly, these results support the notion of fundamental differences in endophenotype profiles across SCZ patient strata that are linked to distinct liabilities across multiple biological processes.

Group 2 showed decreased estimated white blood cell counts and increased neutrophil-to-lymphocyte ratio (NLR), as well as lower estimated CRP levels, suggesting a lower inflammatory state. In line with these findings, group 2 showed decreased liability towards immune-related pathways, cytokines and inflammatory response and complement activation (Figs. 6b, 7b). Moreover, group 2 exhibited a decreased liability towards the development of depression (Fig. 7a, bottom) and an overall better estimated cognitive performance based on various indicators (Fig. 7a, bottom, Supplementary Fig. 21a, b). This was accompanied by a lower predicted expression of presynaptic genes, genes related to synaptic density, and mitochondria as well as an increase in genes related to oxidative damage (Figs. 6b, 7b). Interestingly, group 2 also showed an increase in fractional anisotropy in the corpus callosum based on MRI^42^ with an opposite effect in group 1 (Fig. 7a). Previously, the latter was reported to be decreased in SCZ patients compared to controls. In summary, we conclude that group 2 represents a population of SCZ patients with a less severe disease status.

However, group 2 was characterized by a significantly higher predisposition to metabolic syndrome (MetS) with higher levels of 3 out of 5 risk factors used to define MetS, including (1) higher glucose and HbA1c, (2) lower HDL cholesterol as well as (3) increased weight, BMI and hip circumference (Fig. 7c). It is well known that overall SCZ patients have an increased risk for MetS^43^, but unclear whether this comorbidity would result from a distinct genetic risk factor profile. In line with this observation, group 2 showed genetic liability towards reduced insulin signaling including lower expression of genes modulating the positive regulation of insulin secretion upon glucose stimulation and increased liability towards higher activity of oxidative damage related genes (Fig. 7b, Supplementary Data 11). In line with these findings, group 2 also showed an increased liability towards non-alcoholic fatty liver disease, known to be associated with MetS^44^ (Fig. 7b). These results supplement previous clinical observation on the existence of a MetS subgroup with a genetic and biological basis.

Conversely, group 1 captures a patient group of severe SCZ, with increased inflammatory and substantially reduced cognitive performance parameters (Fig. 7a, Supplementary Fig. 21a, b). These differences on the endophenotype level are reflected in a reduced expression of proteasome degradation, interferon II signaling, plasma membrane, and endoplasmatic reticulum (ER) related genes (Fig. 7b). In contrast, genes related to cytokine and inflammatory response, complement activation as well as related to the presynaptic compartment and postsynaptic density were upregulated (Figs. 6b, 7b).

Jointly, these observations suggest the existence of at least two SCZ patient populations with distinct endophenotypes and consistent biological liability profiles as well as other two groups that represent an intermediate configuration of pathways and endophenotype liabilities.

In order to validate these observations, we turned to a smaller but clinically phenotyped longitudinal cohort of SCZ patients (PsyCourse)^45^. Following the prediction of gene expression levels and projection of *n* = 556 SCZ patients onto the PGC-SCZ patient-derived clustering structure, we reproduced again 3 groups of patients out with similar proportions, excluding the smallest group 4, which did have very few projections (Supplementary Fig. 21c, d). Comparison of differences in *n* = 19 clinical phenotypes revealed a significantly longer duration of illness in group 1 compared to group 2 (p-value = 0.02) and more frequent clinical treatment as in or outpatient (p-value = 0.04) indicative of an increased disease severity in group 1 (Fig. 7d). In addition, group 1 showed a trend reduction in one of the cognitive performance indicators digital symbol test and trail making test compared to group 3 (dg_sym p-value = 0.055 and tmt_A_rt, p-value = 0.078) (Fig. 7d).

In conclusion, CASTom-iGEx patient stratification methodology detected distinct patient groups exhibiting different genetic liabilities that translate into divergent clinical parameters across different complex diseases.

## Discussion

Here, we investigated how heterogeneity in polygenic risk factor distribution can contribute to heterogeneity in clinical parameters, severity, and potential treatment response across patients suffering from complex diseases.

We start to resolve this central problem on the road to stratification medicine by developing a multilayered machine learning approach that relies on the stepwise aggregation of the genetic signals onto biologically relevant entities (genes and pathways) on a per-individuum level. We introduce the concept pathway level association studies and highlight the added value of this strategy in terms of identifying biologically directly interpretable, tissue specific associations and increased detection power.

We show that aggregation of genetic liability through tissue specific gene expression enables the identification of distinct patient subgroups. This approach enables the unsupervised stratification of patients that exhibit distinct genetic liabilities across biological process into subgroups with diverse endophenotypic and clinical profiles. Importantly, this level of biologically and clinically relevant multivariate stratification was not achieved by traditional PRS analysis, highlighting the added value of the CASTom-iGEx approach.

Our results show that the effects of common disease associated genetic variants converge onto distinct cell type specific genes and molecular pathways within subgroups of patients, supporting the notion of distinct patient biotypes. Most importantly, we extensively evaluate well known confounders in genetic stratification analyzes and show that our discovered patient grouping is not compromised by the former.

We show the general feasibility of unbiased patient stratification by applying the CASTom-iGEx pipeline to two fundamentally distinct complex diseases. Moreover, we demonstrate the added value of the biologically informed genotype-based patient stratification using imputed gene expression profiles through detailed clinical and endophenotypic characterization of the discovered patient strata.

This capacity of the CASTom-iGEx pipeline is not dependent on the specific gene expression imputation tool. While we utilize the imputation method PriLer, similar results can be obtained with other imputation methods that can be combined with CASTom-iGEx in a modular plug-and-play fashion. For example, replacing PriLer with gene expression predictions using previously published EpiXcan in conjunction with the CASTom-iGEx pipeline on the UKBB yields highly similar results for CAD with respect to patient grouping, group-specific pathways and endophenotypes (Supplementary Fig. 22, Supplementary Text).

Using the standard CASTom-iGEx workflow, we identify 5 groups of CAD patients with fundamentally distinct risk and disease-relevant endophenotype profiles. This includes a healthier population, a population with reduced levels of blood-circulating LDL, and a decreased frequency of hyperlipidemia concomitant with higher predicted activities of vesicle mediated transport. Finally, we identify a patient group that exhibits a stronger role of inflammatory processes, adding a genetic foundation to the role of inflammation in CAD^46^. Similarly, stratification analysis of schizophrenic individuals revealed substantial heterogeneity in risk factor distribution related to pathomechanisms that have long been implicated to play a key role in SCZ. These include genes and pathways related to neurotransmission, synapse biology, immune system activation and oxidative damage. These analyzes also uncovered the existence of a SCZ patient group with substantially increased genetic loadings for better cognitive performance and lower liability for inflammatory processes, while at the same time showing a higher genetic risk profile for metabolic syndrome.

These results showcase the general utility of the CASTom-iGEx approach in the deconstruction of phenotypic and clinical heterogeneity across patient populations and eventually facilitate precision medicine approaches. While the current results represent an important next step along this road, several key challenges remain.

First, the CASTom-iGEx strategy was only applied in the context of individuals with European ancestry. Application of European ancestry trained models to individuals with Indian ancestries showed overall poor performance and replication of results (Supplementary Fig. 23), consistent with previous observations^47,48^ and requires adaption to a trans-ancestry setting. However, the latter likely requires not only tailoring of statistical models but also generation of new cohorts: While most GWAS hits replicate across populations, there exists substantial variability in effect sizes^47^ and direction of effects for subthreshold associations, concomitant with limited transferability of bona-fide PGS across populations^48^.

As consequence, the generalizability of gene risk score (GRS) based models such as CASTom-iGEx to a trans-ancestry setting through adapted statistical methods^49^ requires the careful calibration using ancestry specific and trans-ancestry GRS models. Moreover, more ethnically diverse cohorts of matching genotype and gene expression data of disease relevant tissues of sufficient size are needed^50^.

Second, the approach presented here constitutes only one step forward towards the biological and translational operationalization of common variants, as it can only be truly effective when combined with other tools and data modalities. Environmental and lifestyle factors dramatically influence disease risk and disease course. Thus, it will be one of the critical next steps to integrate genetic-based insights such as those provided by CASTom-iGEx with deep patient phenotyping information in the context of an unsupervised multi-modal patient clustering framework. In particular, integrating the present approach with multi-omic, imaging, clinical, and exposome-derived data modalities using, e.g., network fusion methods, represents promising avenues to increase the predictive power of patient stratification, specifically towards the prediction of treatment response.

## Methods

All research activities described in this manuscript comply with all relevant ethical regulations. No new data was collected and re-analysis of existing UKBB data was approved by the UKBB.

### Prior Learned elastic-net regression to model gene expression

We developed a methodology called PriLer (*Prior Learned elastic-net regression*) that estimates gene expression from cis‐acting SNPs, combining elastic-net regression with biological annotation of individual genetic variants defined as prior. This includes for example annotation information such as cell type specific chromatin state or GWAS association signal. Since the relevance of each considered biological annotations is a priori unknown, we implemented an iterative learning procedure to obtain optimized weights for each prior in a nested cross-validation fashion (Supplementary Fig. 1 Module 1, Supplementary Fig. 24).

Namely, let $N$ be the total number of genes expressed in a tissue across $M$ individuals, $P$ the total amount of SNPs and indels across all genome and $K$ the number of prior features included. For $n=1,\ldots,N$, we indicate with ${Y}_{n}$ the $M$-length vector of expression of gene $n$ and with ${X}_{n}$ the genotype matrix $M\times {P}_{n}$ of cis-effects for gene $n$ where ${P}_{n}$ is the number of cis-variants distant from the corresponding transcription starting site (TSS) not more than 200 kb. We used 200 kb instead of the usual 1 Mb windows because it has been shown from 3D architecture of the genome that >90% variants/non-coding regulatory elements modulating gene expression in cis are located within 200 kb of the respective TSS^1,2^. Prior information is modeled as a $P\times K$ binary matrix $A$ where 1 indicates that variant $p$ intersects prior feature $k$ (e.g. is in an open chromatin region of cell type $k$).

In elastic-net regression without prior information, gene expression is modeled as a function of cis-variants effects, where the regression coefficients for each gene $n$ are found by solving

1 $${\min }_{{{{{{{\boldsymbol{\beta }}}}}}}_{{{{{{\boldsymbol{n}}}}}}}}\left[\frac{1}{M}\parallel {{{{{{\boldsymbol{Y}}}}}}}_{{{{{{\boldsymbol{n}}}}}}}-{X}_{n}{{{{{{\boldsymbol{\beta }}}}}}}_{{{{{{\boldsymbol{n}}}}}}}{\parallel }_{2}^{2}+{\sum }_{p=1,\ldots,{P}_{n}}L\left({\beta }_{n,p},{\lambda }_{n},{\alpha }_{n}\right)\right]$$

with $L$ being the elastic-net penalty function specific for variant $p$:

2 $$L\left({\beta }_{n,p},{\lambda }_{n},{\alpha }_{n}\right)={\lambda }_{n}\left(\frac{1-{\alpha }_{n}}{2}{\beta }_{n,p}^{2}+{\alpha }_{n}{{{{{\rm{|}}}}}}{\beta }_{n,p}{{{{{\rm{|}}}}}}\right)$$

The problem is solved separately for each gene using glmnet R package^51^ with ${\lambda }_{n}$ and ${\alpha }_{n}$ hyperparameters controlling shrinkage of regression coefficients and ridge/lasso contribution and are optimally found via nested 5-fold cross validation.

In PriLer instead, we hypothesize that variants carrying biological prior information are more likely to be putative regulatory variants (reg-SNPs) i.e. regulating at least one gene. To that end, the penalty referring to a variant $p$ is multiplied by a prior coefficient ${v}_{p}$ obtained as a nonlinear combination through the sigmoid function of prior information in matrix $A$:

3 $${v}_{p}=1-\frac{1}{1+exp \left(-{\sum }_{k=1,\ldots,K}{{\gamma }_{k}A}_{{pk}}\right)}$$

where ${\gamma }_{k}$ represents the prior weight associated to prior feature class $k$ (vector form $\gamma$) and is automatically learned by PriLer through an iterative procedure. Thus, PriLer aims at solving the following problem with respect to ${\beta }_{n}$ for all the genes and the $\gamma$ prior weights vector:

4 $$\mathop{\min }_{{{{{{{\boldsymbol{\gamma }}}}}}{{{{{\boldsymbol{,}}}}}}{{{{{\boldsymbol{\beta }}}}}}}_{{{{{{\boldsymbol{n}}}}}}},n=1,\ldots,N}\left\{\mathop{\sum }_{n=1,\ldots,N}\left[\frac{1}{M}\parallel {{{{{{\boldsymbol{Y}}}}}}}_{{{{{{\boldsymbol{n}}}}}}}-{X}_{n}{{{{{{\boldsymbol{\beta }}}}}}}_{{{{{{\boldsymbol{n}}}}}}}{\parallel }_{2}^{2}+\mathop{\sum }_{p=1,\ldots,P}{v}_{p}L\left({\beta }_{n,p},{\lambda }_{n},{\alpha }_{n}\right)\right]+E\parallel {{{{{\boldsymbol{\gamma }}}}}}{\parallel }_{2}^{2}\right\}$$

Note that since we consider all the genes together, we now iterate through $P$ variants although regression coefficients for variants not in cis-regions of a certain gene $n$ are set to 0. The last term of the objective function represents a regularization term for prior weights and the number of hyperparameters is $2N+1$ i.e. gene-specific${\lambda }_{n},{\alpha }_{n}$ pairs and $E$. We used the sigmoid function to model ${v}_{p}$ to introduce a smooth non-linear change representing variant relevance, ensure the non-negativity of the penalty term as well as differentiability in all the domain of $\gamma$, and introduce a saturation effect such that the penalty term will smoothly and boundedly decrease to zero.

The problem is solved in a 2-step iterative procedure. Initially, prior weights are set to 0 for all the $K$ features. The first step minimizes PriLer function with respect to ${\beta }_{n}$ separately for each gene keeping ${{{{{\boldsymbol{\gamma }}}}}}$ as fixed (hence ${v}_{p}$) via cyclical coordinate descendent algorithm as implemented in glmnet R package; the second step minimizes the PriLer function with respect to ${\gamma }_{k}$ for $k=1,\ldots,K$ keeping ${{{{{{\boldsymbol{\beta }}}}}}}_{{{{{{\boldsymbol{n}}}}}}}$ fixed through globally-convergent method-of-moving-asymptotes implemented in nloptr R package^52^. The algorithm stops until convergence is reached in term of the maximum number of iterations (default = 20) or minimal decrease (default = 0.001) of the objective function from previous step.

In general, the lower the prior coefficient ${v}_{p}$, the less will the corresponding regression coefficient for variant $p$ shrink to zero for all the genes. Hence, the more relevance the variant will have in the gene expression prediction. On the other hand, the weights for the prior features ${\gamma }_{k}$ are dependent on putative reg-SNPs across all the genes that have prior information not zero: the more there are reg-SNPs intersecting a certain prior feature, the higher the correspondent prior weight will be. It is also worth noting that, for prior features intersecting a considerable higher number of variants, the corresponding prior weight will be higher since by chance that prior feature intersects more reg-SNPs. However, in the iterative procedure, if that prior feature is not actually relevant for that tissue-regression model, the corresponding weight remains stable and does not increase (see “Evaluation of prior weights selection in PriLer through random prior simulation” section).

Since PriLer uses the combined information across all genes to derive prior weights, we do not want to introduce noise in that estimation due to genes that are poorly explained by cis-effects. Hence, we estimate prior weights using only heritable genes for which a non-null proportion of variation in gene expression is determined by genetic effects. The list of heritable genes for GTEx and CMC are downloaded from http://gusevlab.org/projects/fusion/ database of TWAS method^12^ (reference functional data), where heritability is estimated for each gene from cis-SNPs via REML algorithm implemented in GCTA^53^. Heritable genes are defined as those having heritability p-value <0.01 estimated in GTEx v7 (https://gusevlab.org/projects/fusion/weights/GTEX7.txt) and CMC. A gene expression prediction model is built for all the genes that have cis-variants in the predefined window. In case of not heritable genes, we use prior coefficients ${v}_{p}$ estimated from heritable genes only.

To find an optimal hyperparameter configuration and evaluate gene expression prediction models, we implemented PriLer in a nested 5-fold cross-validation (CV) setting dividing the procedure in 4 steps (Supplementary Fig. 1). The first step involves heritable genes only and estimates gene expression using elastic-net regression (enet) without prior information. The inner CV finds the optimal ${\alpha }_{n},{\lambda }_{n}$ combination for each gene $n$ separately that minimizes the mean squared error (MSE) on test folders, the outer CV instead builds enet models based on the optimal hyperparameters and evaluates each gene-model via average ${R}^{2}$ on the test folders (${R}_{{cv}}^{2}$).

The second step uses ${\alpha }_{n},{\lambda }_{n}$ combination found in step 1 and builds PriLer models in the outer CV across all heritable genes for different values of hyperparameter $E$, which controls $\gamma$ module. The optimal $E$ parameter is chosen as the one minimizing MSE on the test folds and for that hyperparameters combination ${\alpha }_{n},{\lambda }_{n}$ and $E$ we evaluate PriLer performance based on ${R}_{{cv}}^{2}$. The third step creates a final model for each gene applied to all $M$ samples that will be further used in the external prediction to genotype-only data. Hence, from a single CV, optimal ${\alpha }_{n},{\lambda }_{n}$ combination for enet is found and used in PriLer together with optimal $E$ parameter found in step 2. Finally, the fourth step is used to build PriLer (and enet) models for not heritable genes: step from 1 to 3 are repeated but prior weights ${\gamma }_{k}$ and consequentially prior coefficients ${v}_{p}$ are kept fixed as obtained in step 2 and step 3 (for evaluation and final model creation).

In summary, we obtain ${R}_{{cv}}^{2}$ that estimates PriLer and enet performance, gene expression prediction models together with the corresponding ${R}^{2}$ computed across all samples and for all the genes having cis-variants in 200 kb window.

The algorithm we implemented is inspired by the Lirnet algorithm^54^, however, PriLer is adapted to large reference panels of matched genotype and gene expression data, uses a simplified formula for computing the prior coefficients, and optimizes α and λ penalty parameters instead of using the same penalty across all genes, thus allowing for differences in gene sparsity.

We introduce in PriLer the possibility to model also effects from cofounders to gene expression and variant-gene interaction in a linear manner. In this case, the first term of the objective function representing the prediction squared error becomes:

5 $$\parallel {{{{{{\boldsymbol{Y}}}}}}}_{{{{{{\boldsymbol{n}}}}}}}-{X}_{n}{{{{{{\boldsymbol{\beta }}}}}}}_{{{{{{\boldsymbol{n}}}}}}}{-Z{{{{{{\boldsymbol{\mu }}}}}}}_{{{{{{\boldsymbol{n}}}}}}}\parallel }_{2}^{2}$$

With $Z$ the $M\times C$ confounder matrix unique to all the genes and ${\mu }_{n}$ the corresponding regression coefficient specific to gene-model$n$. The penalty factor term however does not change, being applied only to genotype data. This is practically achieved via the *penalty.factor* option of glmnet set to zero in correspondence of the confounders position so that they are included in all the models for gene expression.

Finally, in order to evaluate PriLer performance as well as enet, we used ${R}^{2}$ in the sense of fraction of deviance explained by the model as implemented in glmnet (dev.ratio). In our model, we explicitly account for linear confounder effects as well as their interaction with cis-variants due to the probable not orthogonal effect, especially, between variants and genetically derived ancestry components. However, we are mostly interested in the variance that can be explained by genotype only. Consider $\widehat{Y}$ as the predicted gene expression vector estimated by the model for a certain gene

6 $$\widehat{{{{{{\boldsymbol{Y}}}}}}}{{{{{\rm{:=}}}}}}X\widehat{{{{{{\boldsymbol{\beta }}}}}}}+Z\widehat{{{{{{\boldsymbol{\mu }}}}}}}$$

and $\bar{Y}$ the mean original gene expression, let $\parallel \cdot {\parallel }_{2}$ be the Euclidean norm operator and $\langle \cdot,\cdot \rangle$ be the scalar product operator among 2 vectors, then ${R}^{2}$ can be formulated as

7 $$1-\frac{\parallel {{{{{\boldsymbol{Y}}}}}}-{\widehat{{{{{{\boldsymbol{Y}}}}}}}\parallel }_{2}^{2}}{\parallel {{{{{\boldsymbol{Y}}}}}}-{\bar{Y}\parallel }_{2}^{2}}=\frac{\parallel {\widehat{{{{{{\boldsymbol{Y}}}}}}}}-{\bar{Y}\parallel }_{2}^{2}+2{{\langle }}{{{{{\boldsymbol{Y}}}}}}-{\widehat{{{{{{\boldsymbol{Y}}}}}}}},{\widehat{{{{{{\boldsymbol{Y}}}}}}}}-\bar{Y}{{\rangle }}}{{\sigma }_{{{{{{\boldsymbol{Y}}}}}}}^{2}}$$

For this reason, we split ${R}^{2}$ in three components: ${R}_{g}^{2}+{R}_{c}^{2}+{R}_{g,c}^{2}$ (see Appendix A) with

8 $${R}_{g}^{2}=\frac{\parallel \widehat{{{{{{\boldsymbol{W}}}}}}}-\bar{W}{\parallel }_{2}^{2}+2 < {{{{{\boldsymbol{W}}}}}}-\widehat{{{{{{\boldsymbol{W}}}}}}},\widehat{{{{{{\boldsymbol{W}}}}}}}-\bar{W} > }{{\sigma }_{{{{{{\boldsymbol{Y}}}}}}}^{2}}$$

9 $${R}_{c}^{2}=\frac{\parallel \widehat{{{{{{\boldsymbol{V}}}}}}}-\bar{\widehat{V}}{\parallel }_{2}^{2}}{{\sigma }_{{{{{{\boldsymbol{Y}}}}}}}^{2}}$$

10 $${R}_{g,c}^{2}=\frac{2 < {{{{{\boldsymbol{W}}}}}}-\bar{W},\widehat{{{{{{\boldsymbol{V}}}}}}}-\bar{\widehat{V}} > }{{\sigma }_{{{{{{\boldsymbol{Y}}}}}}}^{2}}$$

where $\widehat{{{{{{\boldsymbol{W}}}}}}}:=X\widehat{{{{{{\boldsymbol{\beta }}}}}}}$ is the predicted genotype effect, ${{{{{\boldsymbol{W}}}}}}:={{{{{\boldsymbol{Y}}}}}}{{{{{\boldsymbol{-}}}}}}Z\widehat{{{{{{\boldsymbol{\mu }}}}}}}$ is the gene expression vector corrected for the confounder effect hence carrying supposedly only the genotype effect and $\bar{W}$ the corresponding mean, $\widehat{{{{{{\boldsymbol{V}}}}}}}:=Z\widehat{{{{{{\boldsymbol{\mu }}}}}}}$ is the predicted confounder contribution and $\bar{\hat{V}}$ the corresponding mean. Hence, ${R}_{g}^{2}$ represents the part of the variance in gene expression that is due to the genetic component, ${R}_{c}^{2}$ is the contribution of confounders and ${R}_{g,c}^{2}$ represents the joint effect between two. For simplicity, throughout the text we will refer to ${R}_{g}^{2}$ as ${R}^{2}$ and average ${R}_{g}^{2}$ in cross validation as ${R}_{{cv}}^{2}$.

### Reference panels for training gene expression models

Gene expression prediction models are built based on matched data composed of gene expression and genotype individual dosages, also referred to as reference panels. We used GTEx v6p^25^ that includes donors across 44 non-diseased post-mortem tissues and cell lines and CommonMind Consortium (CMC) Release1^26^ composed of RNA-Seq data extracted from post-mortem dorsolateral prefrontal cortex (DLPC) for patients with schizoaffective disorders and controls.

For genotype preprocessing, REF and ALT alleles were aligned to human reference genome hg19 and variants were filtered out based on imputation quality score (INFO) < 0.8, minor allele frequency (MAF) < 0.05 and deviation from Hardy-Weinberg Equilibrium (HWE) P < 5e-5 as well as removal of multiallelic position. Since GWAS data is optionally used as prior information in PriLer, genotype data was matched with CAD and SCZ GWAS summary statistic obtained from^10^ and^11^ in case of GTEx and only SCZ in case of CMC such that only variants with the same position and REF/ALT annotations are kept. Genotype probabilities were then converted to 0-2 dosages where 0 refers to REF/REF configuration and the final number of variants was 6,486,416 and 6,491,178 for GTEx and CMC respectively across 22 autosomal chromosomes.

For RNA-sequencing data, we followed the respective guidelines used to process data for eQTL analysis by the 2 consortia. In case of CMC, we used ‘SVA corrected excluded ancestry’ gene expression processed data that includes residuals from weighted regression through voom-based log transformed CPM (read counts per million total reads) and correspondent observation weights corrected for chosen confounders (see^26^ for details). In case of GTEx instead, we excluded poor quality samples (sample attributes SMAFRZE column equals to ‘EXCLUDE’), considered only the ones matching genotype data and excluded tissues with less than 70 resulting samples. We then followed the GTEx guidelines for eQTL analysis^8^ i.e. for each tissue, genes such that RPKM > 0.1 in at least 10 individuals and number of reads $\ge$ 6 in at least 10 individuals were retained, RPKM expression values were quantile normalized to the average empirical distribution observed across samples and expression values were inverse quantile normalized to a standard normal distribution for each gene across samples. We additionally excluded from the analysis tissues sex-specific and tissues not matching any prior features (see below), resulting in a total of 33 tissues. Finally, genes were annotated using Ensembl on GRCh37 via biomaRt (Bioconductor), in order to define transcription starting site (TSS).

For covariates included in the PriLer model, we followed again the guidelines for eQTL analysis in the respective consortia. In particular, for CMC we used 5 ancestry components provided and computed via GemTools based on a set of high-quality autosomal SNPs from pre-imputed data. For GTEx instead, we included as covariates individual sex, genotype array platform, PEER components calculated from normalized expression matrices for each tissue separately with the number of PEER factors determined as a function of the tissue sample size (N): 15 factors for *n* < 150, 30 factors for 150 $\le$*n* < 250 and 35 factors for N $\ge$ 250 and finally the first 3 principal components (PCs) from genotype data computed using EIGENSTRAT as implemented in Ricopili (see (*5*) for details). We included in our analysis only samples with European ancestry: CMC ethnicity ‘Caucasian’ and GTEx reported race ‘white’ for a total of 478 samples (212 controls and 266 cases) and 377 respectively.

Our methodology incorporates prior information into elastic-net regression. To that end, we used as prior features cell-type specific open chromatin regions one-hot encoded and included CAD GWAS summary statistic^55^ for tissues related to CAD and SCZ GWAS summary statistic^41^ for brain lines and immunological cell types. GWAS information is converted into binary using 0.05 and 0.01 nominal p-values threshold respectively. This disease-specific GWAS thresholds were chosen so that the number of variants having the GWAS prior information was comparable for the two diseases (see GWAS threshold to define PriLer prior section). Importantly, we show that the use of GWAS did not lead to an overfit in trait association nor a significant difference in the distribution of PriLer performances (see GWAS prior does not overfit CAD associations on CARDIoGRAM section). The resulting prior matrix is a binary format with dimension n. of variants times n. of prior features included in the tissue-specific model with 1 indicating either the variant intersects an open chromatin region for that cell type or it passes the nominal GWAS threshold. Open chromatin regions are derived from H3K27ac ChIP-seq data obtained from the Epigenome Roadmap Project as well as ENCODE and merged together (see Supplementary Data 13 for full sample list). In addition, H3K27ac and ATAC-Seq feature-based profiles are combined and included for heart-related tissues obtained from^56^ (GSE72696). For SCZ and brain related tissues, we used ATAC-Seq profiles from human post mortem prefrontal cortex neuronal cells from^57^ (GSE83345). All annotation information can be downloaded from the supplemental website at 10.6084/m9.figshare.24625350.v1. The brain related prior features from ATAC-Seq (*FPC_neuronal_ATAC_R2* and *FPC_neuronal_ATAC_R4*) were modified due to the reduced number of included putative gene regulatory elements (GREs) compared to the H3K27ac derived features (number of GREs 44,475 and 34,883 versus mean number 128,817.3) and a consequence reduction in the number of variants with those priors that would have greatly penalized the correspondent PriLer prior weight (see below for detail). Hence, for each GREs of these 2 prior features, we extended it by half median length of GREs in H3K27ac data (1192) in both directions.

With the purpose of not introducing noise in the selection of these prior features, the weights are solely estimated from heritable genes (see *“* Prior Learned elastic-net regression to model gene expression” section). The complete list of tissue-specific gene expression model, number of samples, number of genes and prior features can be found in Supplementary Table 1 and tissue specific usage for each prior in Supplementary Data 13. Tissue-specific trained models are also available here 10.6084/m9.figshare.22347574.v2.

### Comparison of Priler against existing methods: FUSION, PrediXcan and EpiXcan

We compared PriLer to TWAS^12^ (FUSION), PrediXcan^13^ and EpiXcan^27^ build on GTEx v6p (EpiXcan v7p) and CMC datasets. Summary of tissue models for PrediXcan were downloaded from https://s3.amazonaws.com/predictdb2/deprecated/download-by-tissue-HapMap/ and https://github.com/laurahuckins/CMC_DLPFC_prediXcan/blob/master/DLPFC_oldMetax.db.tar.gz, for FUSION from https://data.broadinstitute.org/alkesgroup/FUSION/WGT/GTEx.ALL.tar and https://data.broadinstitute.org/alkesgroup/FUSION/WGT/CMC.BRAIN.RNASEQ.tar.bz2 and for EpiXcan from https://bendlj01.dmz.hpc.mssm.edu/epixcan/about.php (however, since then the models were moved to https://www.synapse.org/#!Synapse:syn52745629).

To directly compare gene-wise performances between PriLer and other tools, we focused on Liver tissue only. First, we compared gene ${co}{r}_{{cv}}^{2}$ between PriLer and each of the rest of the tools (Supplementary Fig. 3a). For comparisons with PrediXcan and FUSION we used ${co}{r}_{{cv}}^{2}$ defined as squared correlation between ${{{{{{\boldsymbol{W}}}}}}}_{{{{{{\boldsymbol{test}}}}}}}$ and ${\widehat{{{{{{\boldsymbol{W}}}}}}}}_{{{{{{\boldsymbol{test}}}}}}}$ defined as adjusted gene expression and predicted expression from genetic effects respectively combing all test folds (similarly to what was computed in PrediXcan and FUSION). For comparison with EpiXcan, we used the squared correlation between ${{{{{{\boldsymbol{W}}}}}}}_{{{{{{\boldsymbol{test}}}}}}}$ and ${\widehat{{{{{{\boldsymbol{W}}}}}}}}_{{{{{{\boldsymbol{test}}}}}}}$ but averaged across test folds, in order to use the same procedure that was used in EpiXcan). We consider only genes in PriLer having any 200 kb cis-variants and being also present in FUSION, PrediXcan or EpiXcan summary statistics. In addition, we compared the number of regulatory SNPs defined as SNPs selected by each model to predict expression of at least one gene (Supplementary Fig. 3b). In order to assess the biological relevance of the regulatory SNP sets, we evaluated their enrichment in a catalog of 410 functional genomic annotations comprising DNAse hypersensitivity sites (http://hgdownload.cse.ucsc.edu/goldenpath/hg19/encodeDCC/wgEncodeRegDnaseClustered/wgEncodeRegDnaseClusteredV3.bed.gz) across 124 cell types tissues from ENCODE^58^, 338 transcription factors in 130 cell types from ENCODE^59^ and H3K27ac regions across 87 cell types and tissues (Supplementary Data 13).

(http://hgdownload.cse.ucsc.edu/goldenpath/hg19/encRegTfbsClustered/encRegTfbsClusteredWithCells.hg19.bed.gz).

For the latter analysis, we focused on EpiXcan only as it was already shown that this tool shows the highest enrichment for likely biologically relevant SNPs. To that end, we determined the number of regulatory SNPs in the liver model from PriLer and EpiXcan that did overlap and did not overlap with each of the 410 functional genomic annotations. We then performed Fisher’s exact test for each annotation separately to determine whether PriLer selected regulatory SNPs were more likely to be enriched compared to EpiXcan regulatory SNPs or vice versa. Resulting p-values were corrected for multiple testing using the BH method. Results were ordered by odds ratios of enrichment and plotted in Supplementary Fig. 3c (left y-axis). In addition, cumulative fraction of annotations significantly enriched (FDR ≤ 0.01) among PriLer selected SNPs (red line, right y-axis) or EpiXcan (black line, right, y-axis) is also depicted in Supplementary Fig. 3c.

### Genotype-only datasets preprocessing

To impute gene expression from PriLer in large-scale genotype-only datasets, the first step is to match genetic data with reference panels (GTEx and CMC). In particular, for UK Biobank (UKBB), we used imputed data from third release, aligned REF and ALT allele to hg19 and excluded samples due to non-white British ancestry and withdrawn consent. As post-imputation QC, we filtered variants based on SNP call rate <0.98, INFO < 0.8, MAF < 0.05 and HWE p-value < 1e-6 as well as multiallelic positions. We then excluded relatives up to 3rd degree based on kinship matrix such that the largest amount of samples not related would be retained, following UKBB guidelines^60^. Additional samples with no matching submitted and inferred gender and poor-quality ones being outliers for heterozygosity and missing rates are excluded. Our final set after quality control included 340,939 individuals. Genotype data was separately matched with previously processed GTEx and CMC imputed genotype excluding variants having differences in ALT frequency > 0.15 (as described in Aguet et al. ^25^ to match GTEx and 1000 Genome reference) resulting in 5,728,140 and 5,774,100 variants respectively. For CAD application, we used as replication 9 case-control European ancestry cohorts from CARDIoGRAM consortium^28^: German Myocardial Infarction Family Studies (GerMIFS) I, II, III, IV, V, the LUdwigshafen RIsk and Cardiovascular Health Study (LURIC), Cardiogenics (CG), Wellcome Trust Case Control Consortium (WTCCC), Myocardial Infarction Genetics Consortium (MIGen). Pre-imputation QC was performed on each cohort separately using the following criteria: individual call rate ≥ 0.98, SNP call rate > 0.98, minor allele frequency (MAF) > 0.01, concordant recorded and genotype-derived gender, population outliers excluded (deviate beyond mean ± 5x standard deviation) for top two dimensions from the multidimensional scaling (MDS) analysis, PI_HAT < 0.0625 (individuals more distant away than fourth-degree relatives) in the identity-by-descent (IBD) analysis, heterozygosity rate within mean ± 3 x standard deviation, and HWE p-value > 1e-6. Imputation was performed on each cohort separately using the Haplotype Reference Consortium panel on the Sanger Imputation Server (https://www.sanger.ac.uk/science/tools/sanger-imputation-service). Post-imputation QC was then performed with the following criteria; SNP call rate > 0.98, MAF > 0.05, HWE p-value > 1e-6, INFO score ≥ 0.8, multiallelic position excluded and PI_HAT < 0.0625 in IBD analysis for individuals. We then considered all the cohorts together to remove up to fourth-degree relatives (PI_HAT < 0.0625), keeping if possible individuals annotated as cases and/or with the lowest missing rate. Finally, only variants in common across all the cohorts were retained as well as with the aforementioned UKBB-GTEx matched genotype set and such that ALT frequency differences for each pair of cohort/UKBB/GTEx dataset did not exceed 0.15. This procedure yield to a total of 26,681 individuals across the 9 cohorts and 4,257,718 variants matching CARDIoGRAM cohorts, UKBB and GTEx genotyping data. GTEx tissue models adopted for CAD analysis are composed of 2 adipose tissues (subcutaneous and visceral omentum), adrenal gland, 2 artery tissues (aorta and coronary), 2 colon tissues (sigmoid and transverse), 2 heart tissues (atrial appendage and left ventricle), liver and whole blood.

For SCZ application instead, we used 36 PGC cohorts of European ancestry from Psychiatric Genomic Consortium (PGC) for SCZ wave2^41^. Following PGC guidelines, for each cohort we excluded imputed variants based on MAF < 0.01, INFO < 0.6, multiallelic positions and variants that were missing in at least 20 samples (genotype certainty <0.8). Prior to matching variants with GTEx and CMC, we filtered the reference panels such that INFO ≥ 0.6 and MAF ≥ 0.01 based on European individuals. Finally, variants with ALT frequency differences across all possible pair of dataset > 0.15 are excluded, obtaining a total of 5,912,207 and 5,934,252 SNPs and Indels when matching GTEx and CMC respectively. Individuals across all the cohorts are excluded if diagnosis is not available and samples are duplicated/related or a total of 55,419 individuals. GTEx tissue models adopted for SCZ analysis are composed of 8 brain tissues (caudate basal ganglia, cerebellar hemisphere, cerebellum, cortex, frontal cortex BA9, hippocampus, hypothalamus, and nucleus accumbens basal ganglia) and cell EBV transformed lymphocytes while CMC tissue model is based on dorsolateral prefrontal cortex.

### UKBB phenotype pre-processing and coronary artery disease diagnosis definition

UK Biobank is a large-scale biomedical database and research resource containing genetic, lifestyle and health information from half a million UK participants^60^. We used the available deep phenotyping in two different contexts: i) to define CAD and extract CAD related phenotypes in order to perform TWAS and PALAS as well as detect endophenotype differences and treatment response in CAD cases using as genotype data the matched dataset with CARDIoGRAM cohorts, ii) to perform TWAS and PALAS analysis for SCZ related phenotypes and build endophenotype risk scores (endo-RS) weights to model endo-RS in external cohorts such as PGC.

Similarly to previous CAD HARD definition^61^, CAD diagnosis was determined by either hospital episode or self-reported via questionnaire combining ICD10 and ICD9 codes for myocardial infarction and ischemic heart diseases (I21-I24 and 410-412), old myocardial infarction (I25.2), OPCS-4 codes for procedures for coronary artery bypass graft surgery (CABG) (K40-K46), percutaneous transluminal coronary angioplasty (PTCA) (K49-K50, K75) and self-reported heart attack, PTCA, CABG and triple heart bypass. In addition, we used CAD SOFT definition^61^ to define reference set composed of controls for gene T-scores computation (see “From imputed gene expression to gene T-scores” section). CAD SOFT phenotype was defined with the same requirement of CAD HARD plus individuals reporting ICD9 codes for angina pectoris and coronary atherosclerosis (413-414), ICD10 codes for angina pectoris and chronic ischemic heart disease (I20, I25), and self-reported angina.

Phenotypes we had access under application numbers 34217 and 25214 were processed for subsequent analysis using PHESANT software^62^. PHESANT automatically converts UKBB phenotypes distribution to continuous inverse-rank normalized, ordered categorical, unordered categorical or binary, depending on original data type (continuous, integer, categorical single or multiple). Based on the final category, the correct generalized linear model was applied during TWAS and PALAS: Gaussian for continuous, logistic for unordered categorical and binary or ordinal logistic regression for ordered categorical. In addition, PHESANT automatically removes phenotypes recorded for less than 500 individuals and constant ones across the samples.

Original phenotypes not converted via PHESANT are only used in hypothesis-driven CAD endophenotype analysis in which clinical phenotypes are tested (35 in total, nominal significant results are shown in Supplementary Data 7).

### SHIP-Trend cohort preprocessing

The Study of Health in Pomerania (SHIP-Trend) is a population-based cohort study in West Pomerania (northeast of Germany) and is focused on the prevalence and incidence of common population-relevant diseases and their risk factors. Baseline examinations for SHIP-Trend were carried out between 2008 and 2012, comprising 4420 participants aged 20 to 81 years. Study design and sampling methods were previously described^35^.

Regarding genotyping, data was collected from nonfasting blood samples. A subset of the SHIP-Trend samples was genotyped using the Illumina Human Omni 2.5 array, while the majority of samples were genotypes using Global Screening Array (GSA-24v1). Genotypes were determined using the GenomeStudio 2.0 Genotyping Module (GenCall algorithm). Individuals with a genotyping call rate <94%, duplicates (based on estimated IBD), and mismatches between reported and genotyped were removed. Genotypes were imputed using the HRCv1.1 reference panel and using the Eagle and minimac3 software implemented in the Michigan Imputation Server for pre-phasing and imputation, respectively. Before imputation QC steps include the removal of SNPs with a HWE p-value < 0.0001, call rate <0.95, monomorphic SNPs, variants having position mapping problem from genome build b36 to b37, duplicate IDs, or with inconsistent reference site alleles. As post-imputation QC steps, variants with MAF > 0.05, HWE p-value > 1e-6, INFO score ≥ 0.8 were retained and multi-allelic positions were excluded. Individuals more distant away than fourth-degree relatives in the identity-by-descent (IBD) analysis were kept (PI_HAT < 0.0625). The resulting variants were matched with the final set of 4,257,718 variants harmonized for CARDIoGRAM cohorts, UKBB and GTEx genotyping data (CAD-matched variants). SHIP-Trend variants were matched based on same position and REF/ALT annotation. Variants with ALT frequency differences between SHIP-Trend cohort and GTEx not exceeding 0.15 were kept. This procedure yield to 4,240,949 SNPs in the SHIP-Trend cohort also available in the CAD-matched variants set across 4119 individuals. Finally, gene expression was imputed based on previously trained models of liver and whole blood tissues using CAD-matched variants (see “From imputed gene expression to gene T-scores” section).

Regarding transcriptome analysis, RNA was prepared from whole blood under fasting conditions using the PAXgene Blood miRNA Kit (Qiagen, Hilden, Germany). 500 ng of RNA was reverse transcribed into cRNA and biotin-UTP-labeled via Illumina TotalPrep-96 RNA Amp Kit (Ambion). 3000 ng of cRNA were hybridized to the Illumina HumanHT-12 v3 Expression BeadChips, followed by washing steps as described in the Illumina protocol. Gene expression raw intensity data was generated with the expression arrays were exported from Illumina’s GenomeStudio V 2010.1 Gene Expression Module to the R environment and processed (quantile normalization and log2-transformation) with the lumi 1.12.4 package from the Bioconductor open source software as described elsewhere^63^. Quality-controlled gene expression data and genotyping data were available for 976 SHIP-TREND samples.

### PsyCourse study pre-processing

The PsyCourse Study is a longitudinal, multi-center observational study of patients suffering from severe mental disorders (mainly schizophrenia, bipolar disorder, and recurrent depression) as well as healthy control that were subjected to comprehensive neuropsychological testing^45^ and assessment of disease history. All participants were subjected to genotyping using the Infinium Global Screening Array-24 Kit, version 3.0. Prior to imputation, SNPs were filtered based on MAF$\ge$ 0.01, removal of SNPs HWE P < 0.0001, palindrom SNPs and SNPs with MAF deviating more than 10% for EUR reference populations. Subjects were Sex checked and individuals were filtered based on SNP call rate > 98%, individual call rate > 98% and excluding MDS outliers. Genotypes were imputed using the HRCv1.1 reference panel and using the Eagle and minimac3 software implemented in the Michigan Imputation Server for pre-phasing and imputation, respectively, resulting in 7,712,287 SNPs dosages. Subsequently, SNP names were changed to rsID and duplicate rsIDs were removed (multiallelic markers and SNP annotation duplicates). This procedure left 556 individuals suffering from SCZ or schizoaffective disorder. The resulting variants were matched with the final set of 5,934,252 variants harmonized for PGC2 cohorts and CMC genotyping data (SCZ-matched variants). Variants with ALT frequency differences between the PsyCourse Study and CMC not exceeding 0.15 were kept, yielding to 5,094,785 SNPs in the PsyCourse Study also available in the SCZ-matched variants set. Finally, gene expression was imputed based on previously trained models of DLPC tissue using SCZ-matched variants (see “From imputed gene expression to gene T-scores” section).

### From imputed gene expression to gene T-scores

After the gene expression prediction model is built on reference panels, the first step is to impute tissue-specific gene expression on genotype-only cohorts based on PriLer models (Supplementary Fig. 1 Module 2). Let $\widetilde{X}$ be the $L\times P$ matrix of dosages for $L$ new individuals. For each reliable gene $n$ (${R}^{2}\ge 0.01$ and ${R}_{{cv}}^{2} > 0$) in a certain tissue, we predict gene expression for $L$ individuals based on cis-effects estimated via PriLer

11 $${{\widehat{{{{{{\boldsymbol{W}}}}}}}}_{n}{{{{{\rm{:=}}}}}}\widetilde{X}\widehat{{{{{{\boldsymbol{\beta }}}}}}}}_{n}$$

In all applications with the only exception of SHIP-Trend Trend cohort and the PsyCourse Study, $P$ variants in the genotype-only datasets and reference panels are matched via the harmonization process described in “Genotype-only datasets preprocessing” section. Thus, ${\widehat{{{{{{\boldsymbol{\beta }}}}}}}}_{n}$ is a P-length vector with non-zero entries only in correspondence of the cis-variants in 200 kb window of the gene $n$ TSS. Instead, the genotype matrix of SHIP-Trend and PsyCourse are composed of a subset of the original CAD-matched variants or SCZ-matched respectively, of dimension $Q < P$. In these cases, gene expression is imputed using $Q$ regression coefficients ${\widehat{{{{{{\boldsymbol{\beta }}}}}}}}_{{{{{{\boldsymbol{n}}}}}}}^{{{{{{\boldsymbol{Q}}}}}}}$ also available in ${\widehat{{{{{{\boldsymbol{\beta }}}}}}}}_{n}$.

We do not use directly predicted gene expression to test for disease association but convert the imputed expression to gene t-scores for each individual. T-scores are generated as individual moderated t-statistic or ordinary t-statistic depending on the sample size due to computational feasibility. For each cohort in PGC and CARDIoGRAM, the samples are divided in a reference set comprising randomly selected 80% of the control individuals as well as the comparison set, composed of the remaining controls plus all the cases. A moderate t-statistic is computed using *eBayes* function from limma R package^64^ between each individual in the comparison set and all the other samples in the reference set, bootstrapping over the controls and averaging across 40 folds. The same procedure is used in SHIP-Trend cohort and the PsyCourse Study however without a priori cases-controls division. Instead, in each repetition 80% of the individuals were randomly selected as the reference set.

In UKBB, due to the large sample size ( ~ 340,000) we defined gene t-score as the ordinary t-statistic for each sample $l$ in the comparison set as $\frac{{\bar{C}}_{n}}{{sd}({{{{{{\boldsymbol{C}}}}}}}_{{{{{{\boldsymbol{n}}}}}}})/\sqrt{{L}_{{ref}}}}$ where ${{{{{{\boldsymbol{C}}}}}}}_{{{{{{\boldsymbol{n}}}}}}}:={\widehat{W}}_{n}\left(l\right)-{\widehat{{{{{{\boldsymbol{W}}}}}}}}_{{{{{{\boldsymbol{n}}}}}}}\left({ref}\right)$ is the vector of singular differences between current sample $l$ and the samples in reference set of size ${L}_{{ref}}$. For CAD analysis, we adopted bootstrapping technique over 10 folds and used as reference set 30% of individuals not annotated as CAD (SOFT) for a total of 92,784 individuals. For SCZ related phenotypes analysis in UKBB instead, we did not use a priori cases-controls division but randomly selected 10 times 20% of the individuals (68,190 in total) as reference set. Differently from the large incidence of CAD in UKBB cohort, individuals with registered schizophrenia disorders were limited to 1022 out of 340,939 considered samples (ICD10 F20-F29, ICD9 295, self-reported schizophrenia). Because they only compose the 0.29% of the total cohort, they are negligible to the actual reference set size, and we simply sampled across the entire population.

Importantly, the use of gene T-scores instead of imputed gene expression leads to a similar distribution of genes across all samples (mean around 0 and variance around 1), removing the dependence on PriLer predicted performances and the correlation among samples present in imputed gene expression (see “Gene T-scores reduce samples correlation and leads to the same distribution for each gene” section).

### Computation of individual-level pathway-scores

From the gene T-scores, we subsequently computed individual level pathway scores. In contrast to previous approaches^65–67^, we do not set a cut-off for gene level significance or perform an enrichment analysis. Instead, for each sample a representative score for the pathway activity is computed as the mean across gene T-scores that belong to a certain pathway. We used as pathway databases Reactome^32^ and Gene Ontology^31^ as default in CASTom-iGEx pipeline and additionally considered Human WikiPathways^68^ as custom gene-sets. In each tissue, gene-sets are defined based on the reliable set in that tissue (${R}^{2}\ge 0.01$ and ${R}_{{cv}}^{2} > 0$) and only pathways that are not redundant (i.e. composed by the same set of genes) are retained, giving priority to more specific gene-sets being composed of a lower number of genes. The advantage of gene T-scores in the computation of pathways instead of directly imputed gene expression relies on the new scaling space.

### Association of genes and pathways with a trait

For both gene T-scores and pathway scores, we separately tested the association of each gene/pathway with a certain trait (Supplementary Fig. 1 Module 2), using *glm* (Gaussian or logistic regression for continuous or binary trait) or *polr* (ordinal logistic regression for ordered categorical) functions in R and correcting for additional covariates. In case of CARDIoGRAM cohorts and UKBB for CAD analysis, we corrected for sex and first 10 Principal Components (PCs) estimated from pre-imputed data. In case of SCZ cohorts, we corrected for 10 PCs (from 1 to 7, 9,15 and 18) as suggested in^41^, correcting for biases due array type and to population structure, that are partially reflected in the phenotypic variability. We used additional covariates in UKBB dataset for CAD analysis when testing blood biochemistry (category 17518) and blood count (category 100081) phenotypes to correct for medication effect affecting blood levels: medication for pain relief, constipation, heartburn (Field 6154), dietary supplements (Field 6155, 6179) and medication for cholesterol, blood pressure and diabetes (Field 6153, 6177). When using UKBB for SCZ related phenotypes instead, we considered as confounders first 10 PCs, age, sex and phenotype specific covariates: for ‘Maximum digits remembered correctly’ (Field 4282) additional covariates are fields 4250, 4253, 4283 and 4285; for Symbol digit substitution (category 122) we tested fields 20158, 20230 and 20245 additionally correcting for fields 20195 and 20200; for T1 structural brain MRI (category 110) we tested all data fields and regional gray matter volumes subclass correcting for scanner coordinates (fields 25756-25759). In general, we refer as gene/pathway Z-statistics as the estimated effect for trait association divided by its standard error.

In case of multiple cohorts (CARDIoGRAM and PGC), we implemented an approach for meta-analysis similar to GWAMA^69^. Namely, a fixed-effect meta-analysis is initially performed for each gene/pathway weighted by the inverse of their variance. In the presence of heterogeneity effects between cohorts tested via Cochran’s statistic (P ≤ 0.001), we adopted a random-effects meta-analysis calculating the random-effects variance component.

Genes and pathways are finally corrected for multiple testing controlling false discovery rate (FDR) using Benjamini-Hochberg procedure for each tissue, removing pathways composed of a single gene and considering each pathway database separately.

Finally, to identify loci harboring associated genes, we defined loci based on gene TSS position, using a window of 200 kb in both directions and merging genes with overlapping window or with boundaries not distant more than 1 Mb.

### GWAS for coronary artery disease

We compare our TWAS and PALAS with two GWAS summary statistics. The first GWAS (simply referred as “GWAS”) is a recent meta-analysis of UK Biobank SOFT CAD GWAS with CARDIoGRAMplusC4D 1000 Genomes-based GWAS and the Myocardial Infarction Genetics and CARDIoGRAM Exome^61^ downloaded from www.CARDIOGRAMPLUSC4D.ORG. The second GWAS, also called “matched GWAS” is performed on UKBB data set using PLINK (v2.00a2LM) software^70^ via --glm option using the same individuals, case-control distribution, covariates as well as SNPs and indels. In both cases, GWAS p-values are adjusted with Benjamini-Hochberg (BH) procedure to be consistent with the correction adopted for TWAS and PALAS results. The first GWAS is used study the novelty of the identified loci from our TWAS. The matched GWAS instead is used to compare GWAS, TWAS and PALAS summary statistics, having kept the same sample size and variants, and to investigate the aggregation of small effects variants into biological mechanisms, i.e. genes and pathways.

### Additional pathway-detection methods

We applied other two state-of-the-art strategies to detect significant pathways in CAD.

The first is based on hyper-geometric test using significantly associated genes from TWAS. For each tissue, we considered genes reliable in a tissue as background. For each pathway detected in a tissue based on the reliably expressed genes, we computed an hypergeometric test using fisher-exact test R function (alternative = ”greater”). We considered as genes in a pathway those genes that are also reliably expressed in the considered tissue and we intersect this set with the genes FDR 0.05.

The second method is based on MAGMA^34^ using a matched GWAS from the UKBB or GWAS results from the summary statistics of a recent large GWAS^71^. MAGMA analysis was performed by first annotating all SNP locations with genes in vicinity using standard parameters and magma –annotate. Subsequently, we performed gene analysis on SNP p-value data using the European reference panel from Phase 3 of the 1000 Genomes project and GO as well as Reactome pathways for subsequent pathway level analysis leaving all parameters at their standard values. Only pathways significant below an FDR of 0.05 were retained for further analysis.

### Pathway characterization and prioritization

To further characterize the significant pathways identified, we split them into two classes based on the corresponding genes significance. Let $\Omega$ be a significant pathway with FDR($\Omega$) $\le 0.05$. Suppose $\Omega$ is defined from $\{{g}_{1},\ldots,{g}_{n}\}$ genes (called original genes) of which $\{{g}_{1},\ldots,{g}_{\widetilde{n}}\}$ ($\widetilde{n}\le n$) are those also reliable in the tissue considered (called T-score genes) and hence used to compute the corresponding pathway score. We divided pathways into two categories. The first category is composed of pathways with at least one gene more significant than the pathway association, i.e. it exists a gene ${g}_{i}\in \{{g}_{1},\ldots,{g}_{\widetilde{n}}\}$ such that p-value (${g}_{i}$) ≤ p-value ($\Omega$). The remaining significant pathways (second category) are then formed by genes all less significant than the pathway itself, i.e. for all ${g}_{i}\in \{{g}_{1},\ldots,{g}_{\widetilde{n}}\}$ it results p-value (${g}_{i}$) ${ > }$ p-value($\Omega$). These are further split in those including at least one gene significant at FDR 0.05 (green) and those having no gene passing FDR 0.05 threshold, hence considered “novel”. Pathways in the first category are perturbed by the action one or more strong effect genes with non-concordant effects, whereas pathways in the second category are disrupted by the aggregation of effects, either from putative targets identified from TWAS or from completely weak signals that would be missed using a p-value cut-off strategy, hence novel.

For group-specific pathway/endophenotype analysis, we only considered group specific pathways (PALAS 2, FDR ≤ 0.1) that were also significantly associated with the respective endophenotype (FDR ≤ 0.1) and plotted a subset of selected results from this pathway group in Fig. 5b. All pathways are listed in Supplementary Data 6.

### Patient stratification based on gene T-scores

For the purpose of stratifying patients based solely on genetically derived data (Supplementary Fig. 1 Module 3), we adopted a graph-based clustering approach similar to the PhenoGraph method^72^ developed to identify clusters in large high-dimensional data sets. This method is well established in the field of gene expression based clustering and also implemented in one of the most popular analysis toolboxes Seurat^73^. Since this method is tailored to (single-cell) gene expression data, well established in the field and highly computational efficient (a key requirement due to the high number of samples on genotype-only cohorts), we chose this general clustering approach. This method relies on the embedding of high-dimensional data points in a graph structure with edges (i.e. similarity) defined from shared overlap in their local neighborhoods. Similarly to previous improved implementations of this method^73^, we apply a modularity optimization technique to obtain well defined clusters, e.g. the recently developed Leiden clustering. Compared to the previous strategy based the Louvain clustering, Leiden clustering ensures well-connected communities^29^. The sparse similarity matrix for each pair of samples based on the number of shared nearest neighbor (SNN) is constructed starting from the scaled exponential similarity kernel^74^ (see below). This allows to capture more complex relationship between data points and to consider the local density of the data due to the customized scaling parameter ${\sigma }_{i,j}$. We therefore opted for this similarity measure which is also widely used in the field.

Prior to clustering, we apply for each tissue the following pre-processing steps to perform features filtering and normalization, and reduce ancestry contribution. First, gene T-scores are clumped at absolute Pearson correlation of 0.9, with the correlation directly estimated from the considered cases and giving priority to genes that are more significant with respect to the disease of interest. In details, genes are sorted from the most to the least significantly associated with the phenotype of interest (CAD or SCZ) based on the TWAS p-value. All genes are initially assigned to a “current set” and the first gene in this list is compared to all the others based on Pearson correlation estimated from that set of samples, the genes with an absolute Pearson correlation > 0.9 are included in the “remove set”. The “current set” is then updated removing the considered genes and the correlated ones above 0.9 threshold and the entire procedure is repeated until “current set” coincides with an empty set. Finally, the set of clumped genes is obtained discarding the genes in the “remove set” from those initially available in the tissue. The aim of this step is to remove highly redundant genes to not inflate the results. The selection of the Pearson correlation threshold for clumping is based on a grid search for all values between 0.1 and 1, using coverage/conductance and number of cluster/loci as a benchmarking criterion (see “Selection of K- Nearest Neighbor parameter and correlation threshold for clumping in clustering” section for empirical derivation).

Second, each gene is standardized removing the average and dividing for sample standard deviation computed across cases $(\frac{x-\mu }{\sigma })$. This step is performed to weigh the contribution of each gene across all patients equally at this step and have them on the same scale. Gene T-score computation from imputed gene expression harmonized the distributions (see “Gene T-scores reduce samples correlation and leads to the same distribution for each gene” section), nevertheless for the clustering we are considering a subset of the original sample space (patients only). Hence, the re-standardization allows to have the same mean-variance across all samples considered in the clustering, before the actual TWAS-rescaling step. Third, standardized gene T-scores are independently corrected for the same PCs considered in TWAS/PALAS, taking the residuals of the gene-specific linear model. This step is crucial to reduce the relevance of population structure in the final clustering (see Supplementary Fig. 5i, Supplementary Fig. 8, Supplementary Fig. 18). Fourth, the corrected gene T-scores are multiplied by the corresponding Z-statistic for trait association (CAD or SCZ) such that i) differences between patients are enhanced and ii) genes that are more relevant for a certain trait will have a higher impact in the clustering decision, despite retaining all the information (see “Benchmark of genes TWAS-scaling in clustering” section). For SCZ clustering on PGC cohorts, the different data sets are merged together via concatenation and the same steps descried before are applied across all samples, even PCs correction on the merged data set due to PCs estimation on the merged cohorts in PGC wave2. Given the data heterogeneity of the different PGC cohorts, we additionally perform outlier removal. In particular, the four steps previously described are performed and outliers are detected as a union across 10 tissues of samples that deviate beyond median ± 6x s.d. for the first 2 UMAP components (minimum distance = 0.01 and n. of neighbor = 20). These SCZ affected individuals are excluded from further analysis and the pre-processing steps are performed again on the filtered set of samples. Across the 36 PGC cohorts, 35 were used for clustering, filtering 165 outliers for a total of 22,827 cases and 1 cohort (scz_boco_eur, 1,773 cases) was used for external validation. In SCZ analysis, the set of variants of PGC cohorts was not harmonized with UKBB data set that is used to approximate missing phenotype information (see “Risk scores computation” section). Thus, to ensure a consistent imputation of the genetic variables, we computed Pearson correlation of impute gene expression and imputed pathway scores between the models built from UKBB and PGC. Genes and pathways are included in the clustering analysis if the correlation between imputation on the reference panels GTEx and CMC between the two genotype-only data sets is higher than 0.8. After pre-processing, we construct a sparse similarity matrix for each pair of samples based on the number of shared nearest neighbor (SNN). We initially computed scaled exponential similarity kernel^74^ between samples $i$ and $j$ as

12 $$K\left(i,j\right)=\exp \left(-\frac{e{d}^{2}\left({{{{{{\boldsymbol{Z}}}}}}}_{{{{{{\boldsymbol{i}}}}}}},\,{{{{{{\boldsymbol{Z}}}}}}}_{{{{{{\boldsymbol{j}}}}}}}\right)}{0.5{\sigma }_{i,j}}\right)$$

with ${ed}\left({{{{{{\boldsymbol{Z}}}}}}}_{{{{{{\boldsymbol{i}}}}}}},{{{{{{\boldsymbol{Z}}}}}}}_{{{{{{\boldsymbol{j}}}}}}}\right)$ the Euclidean distance between normalized gene-level t-scores and

13 $${\sigma }_{i,j}=\frac{{mean}\left({ed}\left({{{{{{\boldsymbol{Z}}}}}}}_{{{{{{\boldsymbol{i}}}}}}},{N}_{i}\right)\right)+{mean}\left({ed}\left({{{{{{\boldsymbol{Z}}}}}}}_{{{{{{\boldsymbol{j}}}}}}},{N}_{j}\right)\right)+{ed}\left({{{{{{\boldsymbol{Z}}}}}}}_{{{{{{\boldsymbol{i}}}}}}},\,{{{{{{\boldsymbol{Z}}}}}}}_{{{{{{\boldsymbol{j}}}}}}}\right)}{3}$$

where $${mean}$$$$\left({ed}\left({{{{{{\boldsymbol{Z}}}}}}}_{{{{{{\boldsymbol{i}}}}}}},{N}_{i}\right)\right)$$ is the averaged Euclidean distance between sample $$i$$ its k closest neighbors. Hence, this initial similarity matrix depends already on the local density of the data due to the customized scaling parameter $${\sigma }_{i,j}$$. However, to sparsify the similarity and give information only on the local interactions, we used the similarity kernel defined above to compute the percentage of shared nearest neighbor (SNN) between samples $$i$$ and $$j$$:

14 $$S\left(i,j\right)=\frac{|{v}_{i}\cap {v}_{j}|}{|{v}_{i}\cup {v}_{j}|}$$

with ${v}_{i}$ the set of k nearest neighbor based on $K$. $S$ matrix represents the weight for edges in the patient graph structure. We fixed the parameter k to define the closest neighbors as 20 (see “Selection of K- Nearest Neighbor parameter and correlation threshold for clumping in clustering” section for empirical derivation). We finally applied Leiden method^29^ implemented in *igraph* R package^75^ to detect communities that would maximize modularity based on SNN graph.

### Polygenic risk score computation in CAD cases

To compute polygenic risk score (PRS) for individuals in UKBB related to CAD phenotype, we used PRSice2 software^76^ with default parameters. We considered as base and target data sets the UKBB cohort with CAD phenotype. The GWAS results for --base input are the matched GWAS summary statistics as described in “GWAS for coronary artery disease”. Distributions among cases and controls division as well as clusters were obtained after standardization of best-fit PRS across all individuals. Of note, the use of the same data set for base (GWAS summary statistic) and target (prediction) cohort leads to overfit in the separation between cases and controls. Nevertheless, the focus of this analysis is not the variance explained of CAD by PRS but rather the similar distribution and non-stratification of the identified cluster of cases as well as the partition of cases in groups based on PRS distribution.

### Detection of genes and biological pathways associated with clustering structure

In order to test for genes and pathways associated with detected clustering structure, we considered each tissue separately and test differences of a certain gene/pathway in $g{r}_{g}$ versus the remaining patients via Wilcoxon-Mann-Whitney (WMW) test implemented in rstatix R package^77^. In each test, the WMW estimates and confidence intervals are computed corresponding to the median difference of the location parameter (Hodges-Lehmann estimator). Let $G$ be the total number of clusters detected, for each group ${gin}1,\ldots,G$ in a tissue, p-values were corrected for multiple comparison using Benjamini-Hochberg procedure to control for false discovery rate. Note that, although the clustering is tissue specific, we tested for differences in gene and molecular pathways across all tissues. Cluster-specific genes were subsequently combined across tissues in loci based on physical location (TSS window 200 kb, merged if distance <1 Mb). To identify cluster-specific pathways, we tested only pathways filtered with the following strategy. For each tissue, we considered pathways both in Reactome and GO composed of at least 3 genes and no more than 200 (both original genes and T-score genes in the pathway). These pathways are then clumped giving priority to those with the highest coverage (ratio between T-score genes and original genes) and highest number of genes used to compute the pathway (T-score genes). The resulting set of pathways have a pairwise Jaccard Index based on gene set not exceeding 0.2.

In addition, we tested pathways in WikiPathway and CommonMind gene-sets^9^ in SCZ without this initially filtering but using all the available pathways.

### Cluster-specific PALAS in CAD (PALAS 2)

In the context of CAD clustering characterization, we also performed a cluster-specific PALAS analysis, referred as PALAS 2. We tested each group of CAD cases detected on liver versus 321,831 non-affected individuals (CAD HARD definition), the same set of controls used in the CAD PALAS analysis. The total number of pathways tested is 36,949 across all tissues (11 from GTEx) and 3 databases (WikiPathways, GO and Reactome). This same set was also tested in the CAD PALAS (non-affected individuals vs CAD HARD, called PALAS 1). Among the 567 pathways associated with CAD from PALAS 1 (FDR 0.05), we reduced this list to unique pathways and consistent across tissues. Namely, we retained the pathways associated only in a tissue and for those available in more than one tissue we kept the one with strongest association to CAD if the Z-statistics were concordant in sign and excluded that pathway otherwise. This led to a final list of 461 unique pathways from PALAS 1. We then divided this set into 2 groups. The first group is composed of those pathways having the same Z-statistic sign across PALAS 1 and PALAS 2 (across all groups and even if not significant in PALAS 2), and those having at least one group Z-statistic discordant in sign with either PALAS 1 or another group from PALAS 2. In addition, we intersected these results with the cluster-specific WMW analysis (see above, referred as WMW group) testing one group versus all the other cases. The filtered list of pathways from WMW group (7,978 across all tissues) was intersected with those significant in PALAS 1 (FDR 0.05), PALAS 2 (FDR 0.05) and WMW group significant (FDR 0.01, see “Clustering simulation in CAD” section for set-up of 0.01 threshold).

### Predict cluster structure and validate gene and pathway signatures

Similarly to PhenoGraph approach, we implemented a projection method based on the percentage of SNN in order to use the detected clustering structure from one cohort to predict groups on external cohorts such as CARDIoGRAM for CAD and scz_boco_eur for SCZ. In particular, for each cohort we considered only genes used in the clustering model and repeated the gene-specific standardization, correction for PCs and Z-statistic multiplication as described in the clustering pre-processing procedure. The Z-statistic for the projection coincides with the one used in the initial clustering and is obtained from the general TWAS. Then, we computed the percentage of SNN based on the exponential similarity kernel as previously described among each pair of individuals in the combined datasets (model plus external cohort). For each sample in the external cohort, the assigned label is based on the probability that a random walk originating at external sample will first reach a labeled sample in the model clustering for each group $G$. The problem is solved via a system of linear equations based on graph Laplacian of the enlarged sample network and each new sample is then allocated to the group that it reaches first with highest probability, see^72^ for details.

We evaluated the projected clustering on external cohorts based on i) the fraction of cases assigned to a certain cluster both in model clustering and projected and ii) the correlation among cluster-relevant genes. The latter is computed for each group as the Spearman correlation of WMW estimates for model clustering and external cohort across all tissues, including only genes that are cluster-relevant (FDR < 0.05) in the model. In addition, we estimated the number of reproduced loci in the external cohort using the identified loci of cluster-relevant genes. For each group $g$, we considered each relevant locus and retained the most significant gene in that locus, we then annotated the locus as replicated if the WMW estimate for that gene has the same sign in model and external cohort.

Similar approach was used to validate discovered patient group-specific biological pathways. Using the projected group structure in external cohorts (CARDIoGRAM), we calculated Wilcoxon-Mann-Whitney estimates for pathway scores in a particular group compared to the rest of the samples for each external cohort (see “Detection of genes and biological pathways associated with clustering structure” section). The resulting estimates were compared to the estimates for common significant (FDR < 0.05) group-specific pathways in UKBB using Spearman correlation.

### Detection of endophenotype differences across patient strata

To test for differences among trait related endophenotypes across patient clusters, we applied generalized linear models to detect group-specific differences, comparing group $g(g{r}_{g})$ versus the remaining samples. More specifically, we applied this strategy for the CAD analysis, leveraging the UKBB deep phenotyping and 635 phenotypes included the following categories: alcohol, arterial stiffness, blood biochemistry, blood count, blood pressure, body size measures, diet, hand grip strength, impedance measures, physical activity, sleep, and smoking (class 1 phenotypes). We also included additional clinical information such as family history, medications, ICD10 diagnosis related to anemia, circulatory system, respiratory system, and endocrine system (class 2 phenotypes). The following phenotypes were excluded: all phenotypes having less than 100 values, binary phenotypes with less than 50 true values and categorical ordinal phenotypes with less than 10 samples in the base category both inside and outside the considered group. Continuous phenotypes were initially standardized $\left(\frac{x-\mu }{\sigma }\right).$ Depending on the nature of the phenotype (continuous, binary or categorical ordinal) and similarly to trait-gene/pathway association, for endophenotype $j$ and group $g$, we applied the following generalized linear model (GLM):

15 $${phen}{o}_{j} \sim g{r}_{g}+{co}{v}_{1}+\cdots+{co}{v}_{l}$$

with $g{r}_{g}$ a binary n. of cases-vector having 1 in correspondence individuals clustered in group $g$. In both class 1 and 2 phenotypes, the covariates included first 10 PCs, age and sex. Additionally, for class 1 we also corrected for medication usage: pain relief medication (aspirin, ibuprofen, paracetamol), vitamin supplements (A, B, C, D, E, folic acid), mineral and dietary supplements (glucosamine, calcium, zinc, iron, selenium), blood pressure medication, cholesterol lowering medication and insulin usage (part of Fields 6154, 6155, 6179, 6153, 6177). Hence, for each endophenotype $j$ and group $g$ we obtained an estimate of group $g$ impact with respect to all the other cases in the form of adjusted regression coefficient ${\beta }_{{GLM}}$ and corresponding p-value tested from normality assumption.

For CAD clustering in liver, we further split the phenotype in two groups: those more strongly informative for CAD (“relevant”: blood biochemistry, blood count, blood pressure, blood size measures, impendence measures, arterial stiffness, hand grip strength, early life factors, family history, height, and ICD10 diagnosis) composed 249 phenotypes and those less relevant to CAD (“control”: alcohol, diet, medication, medications, physical activity, sleep, smoking) composed of 386 phenotypes. The “relevant” and “control” class were separately corrected for multiple test associations using Benjamini-Hochberg procedure (separately per group) and results with FDR$\le$ 0.1 in the “relevant” group where investigated further.

In case of the hypothesis-driven analysis for CAD, we first tested with the same procedure 33 clinical variables among UKBB (Supplementary Data 7) and 2 endophenotypes registered for GerMIFSV (Gensini score and n. of vessel affected). In contrast to the general analysis, clinical variables in UKBB were not converted via PHESANT software but directly used including an additional permutation based p-value. To that end, individuals were randomly assigned to any of the 5 CAD clusters, respecting the original group in liver followed by the same GLM based endophenotype analysis, this was repeated 50 times (see “Patients clustering simulation in CAD” section). We then determined the frequency that a particular clinical variable was nominally (p-value $\le 0.05$) associated with any of the groups in any of the 50 partitions and used this frequency to determine an empirical p-value by dividing by the number of tests. We then retain only clinical variables with an empirical p-value and a GLM based p-value below 0.05.

For the SHIP Trend cohort, both 20 collected clinical variables (imt_auto_t0, ldlch, hdlch, tg_s, igf1, hba1c, crp_hs_re_z, bmi_t0, bia_magermasse, sysbp_t0, diabp_t0, hyp_t0, mi_first_t0, stroke_first_t0, plaque_t0, stenos_t0, fmd_reduced, abi_pathol, mort_all, mort_cvd) and 24,925 measured gene transcripts across 975 samples were tested with the previously described procedure. We included as covariates testing group-specific clinical variable differences the first 10 PCs, sex, genotype array type and medication info for blood pressure, cholesterol lowering and insulin. In addition to these covariates, we also included in the cluster-specific measured gene expression analysis RNA integrity number, amplification batch (96 well plates), sample storage time, white blood cell count, hematocrit, red blood cell count, platelet count as well as neutrophils, lymphocytes, monocytes, and basophiles percentages. To compare the differences in actual gene expression with the imputed one, we considered only group-wise significant genes from UKBB at FDR 0.01 in whole blood. Measured transcripts were restricted to the set of group-specific significant genes from UKBB matched by not null ENTREZ gene ID. P-values for adjusted beta in this subset of transcripts were corrected via Benjamini-Hochberg procedure. In addition, we built pathway-scores in SHIP-Trend cohort from the measured gene expression (called measured pathway-scores) and tested group-specific differences via GLM. These measured pathway-scores are obtained in a similar manner to the predicted gene expression but using all measured genes in the whole blood microarray dataset based on the quantile normalized, z-scored residuals after correction for covariates. Groups with less than 15 measurements were excluded from group-wise comparison.

For the PsyCourse Study, we tested the following phenotypes using the same GLM based procedure evaluating the following variables: v1_nrpsy_tmt_A_rt, v1_dur_illness, v1_age_1st_inpat_trm, v1_age_1st_out_trm, v1_nrpsy_dg_sym, v1_panss_sum_pos, v1_tms_daypat_outpat_trm, v1_bmi, v1_nrpsy_tmt_B_rt, v1_cat_daypat_outpat_trm, v1_cgi_s, v1_nrpsy_mtv, v1_outpat_psy_trm, v1_gaf, v1_nrpsy_mwtb, v1_panss_sum_neg, v1_nrpsy_dgt_sp_bck, v1_fam_hist, v1_nrpsy_dgt_sp_frw, including Age, Sex, center of patient recruitment and the first two PCs from the genotype analysis as covariates.

### Group-specific treatment response analysis in CAD

Taking advantage of the treatment annotation in UKBB data, we investigated whether cases from different genetically detect groups exhibited a different treatment response. For this purpose, we regarded as response phenotypes the categories of arterial stiffness, blood biochemistry, blood count, blood pressure, body size measure, hand grip strength and impedance measures; and we considered as treatments the 17 medications previously described for endophenotype differences analysis (pain relief, vitamin supplements, mineral and dietary supplements, blood pressure medication, cholesterol lowering medication and insulin). Consider group $g$ composed of ${n}_{g}$ cases and consider phenotype $j$ values in corresponding of group $g$ (${phen}{o}_{j}(g{r}_{g})$). Phenotypes with less the 300 available values were excluded, and continuous ones were normalized. The response for medication $i$ (e.g. cholesterol lowering medication) in group $g$ measured based on phenotype $j$ is tested via GLM

16 $${phen}{o}_{j}\left(g{r}_{g}\right) \sim {me}{d}_{i}\left(g{r}_{g}\right)+{co}{v}_{1}\left(g{r}_{g}\right)+\cdots+{co}{v}_{l}\left(g{r}_{g}\right)$$

and we denote as ${\hat{\beta }}_{i,j,g}$ regression coefficient representing treatment $i$ effect on phenotype $j$ in group $g$. We used as covariates first 10 PCs, age, sex as well as all the other treatment binary categories. In order to test differences among treatment-phenotype effects across groups, for each pair of groups ($g,h$) we evaluated regression coefficient differences using Z-test^78^:

17 $${Z}_{i,j}\left(g,h\right)=\frac{{\widehat{\beta }}_{i,j,g}-{\widehat{\beta }}_{i,j,h}}{\sqrt{{\left({SE}{\widehat{\beta }}_{i,j,g}\right)}^{2}+{\left({SE}{\widehat{\beta }}_{i,j,h}\right)}^{2}}}$$

where ${SE}$ is the standard error for regression coefficient computed from GLM. P-values were computed under the assumption of normal distribution and corrected for multiple testing across all the phenotypes but separately for each group-pair ($g,h$) and treatment $j$ taken into consideration.

### Risk scores computation and differences detection in cases stratification

In order to test for endophenotypic differences in datasets without any endophenotypic information such as PGC cohorts, we developed a strategy to annotate patient with endophenotypes from genetic information using tissue-specific endophenotype-risk scores (endo-RS). For each tissue, gene-phenotype association was estimated (TWAS) as previously described in UKBB for phenotype $j$, obtaining for each gene $n$ association Z-statistic${Z}_{n}^{j}=\frac{{\beta }_{n}^{\, j}}{{SE}{\beta }_{n}^{\, j}}$. Secondly, we filtered redundant genes due to LD structure clumping genes at 0.1 squared Pearson correlation cut-off and giving priority to those with higher genotype ${R}^{2}$ imputation. The correlation among genes was estimated via a subset of UKBB samples without CAD HARD diagnosis. Finally, for an external cohort composed of $L$ individuals, endo-RS is defined as the $L$-vector of weighted sum for gene t-scores previously corrected for PCs (${{{{{{\boldsymbol{T}}}}}}}_{{{{{{\boldsymbol{n}}}}}}}L$-vector, for $n=1,\cdots,N$) multiplied by gene-phenotype Z-statistic ${Z}_{n}^{j}$:

18 $${{{{{\boldsymbol{R}}}}}}{{{{{{\boldsymbol{S}}}}}}}^{{{{{{\boldsymbol{j}}}}}}}=\mathop{\sum }_{n=1,\ldots,N}{{{{{{\boldsymbol{T}}}}}}}_{{{{{{\boldsymbol{n}}}}}}}{Z}_{n}^{j}$$

Hence, we obtained a continuous risk score that mimics the actual phenotype not available for PGC cohorts, which was then tested for group-specific differences. Namely, PGC cohorts are combined, and each gene is corrected for PCs as described in the clustering procedure. Endo-RS are then computed with phenotype effect estimated from UKBB and standardized. Finally, cluster differences are tested via GLM with gaussian link function including PCs as covariates and considering the partition of SCZ cases previously computed on PGC cohorts. In SCZ analysis, we leveraged TWAS results for 1,000 phenotypes from UKBB among the categories of alcohol use, anxiety, blood biochemistry, blood count, blood count ratio, blood pressure, body size measure, cannabis use, depression, dMRI skeleton, happiness and well-being, mental distress and health, sleep, smoking, social support, susceptibility-weighted brain MRI, T1 structural brain MRI, task functional brain MRI, traumatic events. In hypothesis-driven analysis, we specifically investigated cognitive function and used TWAS Z-statistic from numeric memory, pairs matching, prospective memory, reaction time, fluid intelligence, symbol digit substitution, trail making.

The reliability of the endo-rs to estimate the actual endophenotype differences depends on i) the number of samples in the gene-endophenotype association analysis together with the genetic heritability of the phenotype and ii) the effect size of the cluster specific difference. The former was measured in UKBB via F-test statistic: endo-rs ability to model actual phenotype was estimated via nested linear models of phenotype predicted via endo-rs plus covariates or only covariates. The latter was estimated via the absolute value of the regression coefficient from GLM cluster differences for endo-rs (${{{{{\rm{|}}}}}}{\beta }_{g}{{{{{\rm{|}}}}}}$ for ${gin}1,\cdots,G$ groups). Hence, we defined a cluster-reliable non-negative measure (CRM) for each endophenotype $i$ and group $g$ as the product of F-statistic and cluster-specific coefficient: ${CRM}(j,g)={Fsta}{t}_{j}\bullet {{{{{\rm{|}}}}}}{\beta }_{g}{{{{{\rm{|}}}}}}$ (see “Validation of gene risk scores to mimic actual phenotype in cluster-specific differences” section for validation).

### Clustering based on genotype derived principal components

To study the ancestry contribution to tissue-specific clustering, we separately cluster cases (CAD or SCZ) solely based on the PCs derived from genotype data. For CAD, we considered the first 40 PCs available in UKBB data set. For SCZ instead we considered the first 20 PCs available and computed jointly in the PGC cohorts. In both diseases, we separately standardized each PCs to mean 0 and standard deviation 1 and performed Louvain clustering on shared nearest neighbor network built from the available PCs. We then compared the obtained clustering structure to those obtained from the actual tissues via NMI and compared it to the 10,000 random partitions of cases of the same size (Supplementary Fig. 8c, Supplementary Fig. 18c). To investigate the overlap at the single group level, we additionally computed the odds ratio from Fisher’s Exact test comparing each pair of groups from PCs and imputed gene expression, namely individuals in gr_i_ (PC) and outside gr_i_ (PC) with individuals in gr_j_ (imputed expression) and outside gr_j_ (imputed expression) (Supplementary Fig. 8d, Supplementary Fig. 18d). Finally, endophenotype differences in PC clustering was performed via previously described GLM approach but only correcting for age and sex covariates. To compare endophenotype differences, we considered for each endophenotype tested the group reaching highest significance (lowest p-value) and compared -log_10_p-value between clustering based on PCs and based on imputed gene expression (Supplementary Fig. 8f, Supplementary Fig. 18e).

### Comparison of CASTom-iGEx clustering with PRS-based partition

We computed the PRS for CAD affected individuals based on their genotype (see “Polygenic risk score computation in CAD cases” section) and partitioned the samples based on the quartiles cut-offs, obtaining four equally sized groups (gr1 = 0%-25%, gr2 = 25%-50%, gr3 = 50%-75%, gr4 = 75%-100%). To compute the variance explained by PRS partitions for CAD endophenotypes, we considered only CAD affected individuals and used a nested linear models approach. In particular, we compared the full model M1: pheno ~ groups + cov with the covariates only model M2: pheno ~ cov. The group information is converted into one-hot encoding format with n features equals to the number of groups and 1 indicating the cluster membership. The covariates used are the same as those for the cluster-specific endophenotype analysis. The variance explained by the PRS partition R^2^ is obtained as the difference between R^2^ from the full model M1 and R^2^ from the covariates only model M2. Note that the values are not scaled via the liability scale and their range is small. Nevertheless, the point aim of this analysis is the comparison between two clustering structures (CASTom-iGEx method and PRS partition) which would share the same liability scale of the phenotype.

### CASTom-iGEx framework with EpiXcan gene expression models

EpiXcan model for liver (trained on GTEx data) was downloaded on 03.07.23 from https://bendlj01.dmz.hpc.mssm.edu/epixcan/about.php (however, since then the models were moved to https://www.synapse.org/#!Synapse:syn52745629). The original implementation of PrediXcan (https://github.com/hakyimlab/PrediXcan) was used to predict expression on the UKBB genotype data with the EpiXcan model. Notably, due to the fact that the UKBB data was previously harmonized with CAD GWAS data, only 60,067 out of 147,349 (40.77%) SNPs used by the EpiXcan model were present in the harmonized genotype data. After prediction, the imputed gene expression data was filtered to remove genes for which the q-value of prediction performance of the EpiXcan model (contained in the metadata of the model) was higher than 0.01 and genes which were predicted to have no expression in all of the samples. Thereafter, the standard PriLer and CASTom-iGEx workflow starting from the calculation of gene T-scores was performed.

CAD patients based on both PriLer gene expression models and EpiXcan models were clustered in liver (results in Supplementary Fig. 22).

### Reporting summary

Further information on research design is available in the Nature Portfolio Reporting Summary linked to this article.

### Supplementary information

Supplementary Information

Peer Review File

Description of Additional Supplementary Files

Supplementary Data 1

Supplementary Data 2

Supplementary Data 3

Supplementary Data 4

Supplementary Data 5

Supplementary Data 6

Supplementary Data 7

Supplementary Data 8

Supplementary Data 9

Supplementary Data 10

Supplementary Data 11

Supplementary Data 12

Supplementary Data 13

Reporting Summary

## Data Availability

All summary level statistics are reported in the supplementary tables or supplementary data sets. See legends for these files for detail. The UKBB data are privacy protected and access can be requested through the UKBB data portal https://www.ukbiobank.ac.uk/. The GTEx data are available through dbGAP accession number phs000424 (originally v7.p2 downloaded on 11/28/2018): https://www.ncbi.nlm.nih.gov/projects/gap/cgi-bin/study.cgi?study_id=phs000424.v9.p2 .The PGC data are privacy protected and can be accessed through a secondary analysis proposal sponsored by a PGC-SCZ working group PI member that needs to be approved by the working group. Data access instructions can be found at: https://pgc.unc.edu/for-researchers/data-access-committee/data-access-information/. The German cohorts of CARDIoGRAM consortium is privacy protected and can be accessed through collaboration with PIs of the consortium, e.g. through HS, see http://www.cardiogramplusc4d.org/data-downloads/. The PsyCourse Study data are privacy protected but can be accessed by submitting a research proposal (see http://www.psycourse.de/openscience-en.html). The genotype and gene expression data from the CommonMind consortium is privacy protected and can be accessed via the CommonMind knowledge portal: 10.7303/syn2759792. The SHIP-Trend study genotype data is privacy protected and can be accessed through the study PIs: https://www.maelstrom-research.org/study/ship. The trained tissue specific PriLer models on GTEx v6p and CMC release 1 reference panels are available at 10.6084/m9.figshare.22347574.v2. TWAS and PALAS summary statistics for CAD and SCZ can be found at 10.6084/m9.figshare.22495561.v1.

## Code availability

The software pipeline is based on R and is available at https://github.com/zillerlab/CASTom-iGEx. The code related to the paper analyzes is available at https://github.com/zillerlab/CASTom-iGEx_Paper.
