# Supplementary material for: Distinct genetic liability profiles define clinically relevant patient strata across common diseases
Source: Nat Commun. 2024 Jul 1;15:5534. doi: 10.1038/s41467-024-49338-2 (PMC11217418; doi:10.1038/s41467-024-49338-2)
Supplement: Supplementary file 1 — Supplementary Information [file 41467_2024_49338_MOESM1_ESM.pdf]

Supplementary Information for

Distinct genetic liability profiles define clinically relevant patient strata across common diseases

Lucia Trastulla[1,2,3,4], Georgii Dolgalev[4,], Sylvain Moser[1,2,5], Laura T. Jiménez-Barrón[1,5], Till F.M. Andlauer[1,6], Moritz von Scheidt[7,8], Schizophrenia Working Group of the Psychiatric Genomics Consortium, Monika Budde[9], Urs Heilbronner[9], Sergi Papiol[1,9], Alexander Teumer[10,11,12], Georg Homuth[13], Henry Völzke[10,11], Marcus Dörr[10,14], Peter Falkai[1,15], Thomas G. Schulze[9], Julien Gagneur[16,17,18], Francesco Iorio[3], Bertram Müller-Myhsok[1,21], Heribert Schunkert[7,8] & Michael J. Ziller[1,4,22]*

*Correspondence to: ziller@uni-muenster.de

This PDF file includes:

26 Supplementary Figures
1 Supplementary Table
Supplementary Note 1
Supplementary Note 2

Supplementary Figures

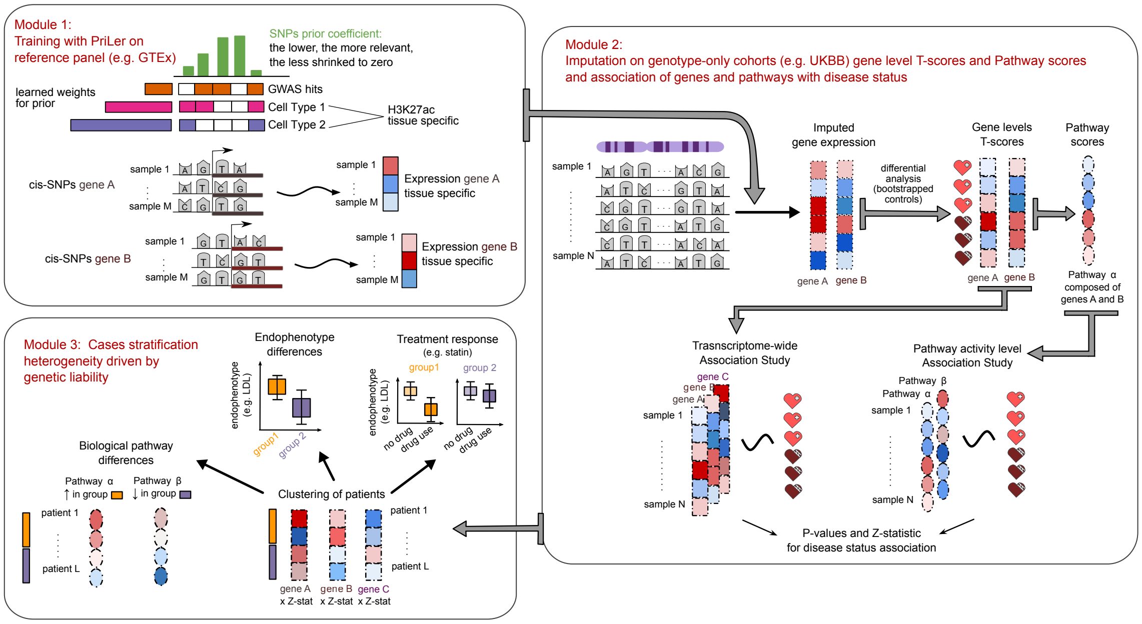

Supplementary Fig. 1. Workflow CASTom-iGEx pipeline.
CASTom-iGEx is composed of 3 modules: 1) Prior Learned elastic-net regression (PriLer) to train gene expression prediction models on reference panels integrating multi-dimensional biological annotation of common variants, 2) application to genotype-only datasets, conversion to gene levels T-score/pathway levels scores and identification of differentially active genes and pathways between affected and unaffected individuals, 3) patient stratification based on gene levels T-score to characterize biological pathways and endophenotypic differences.

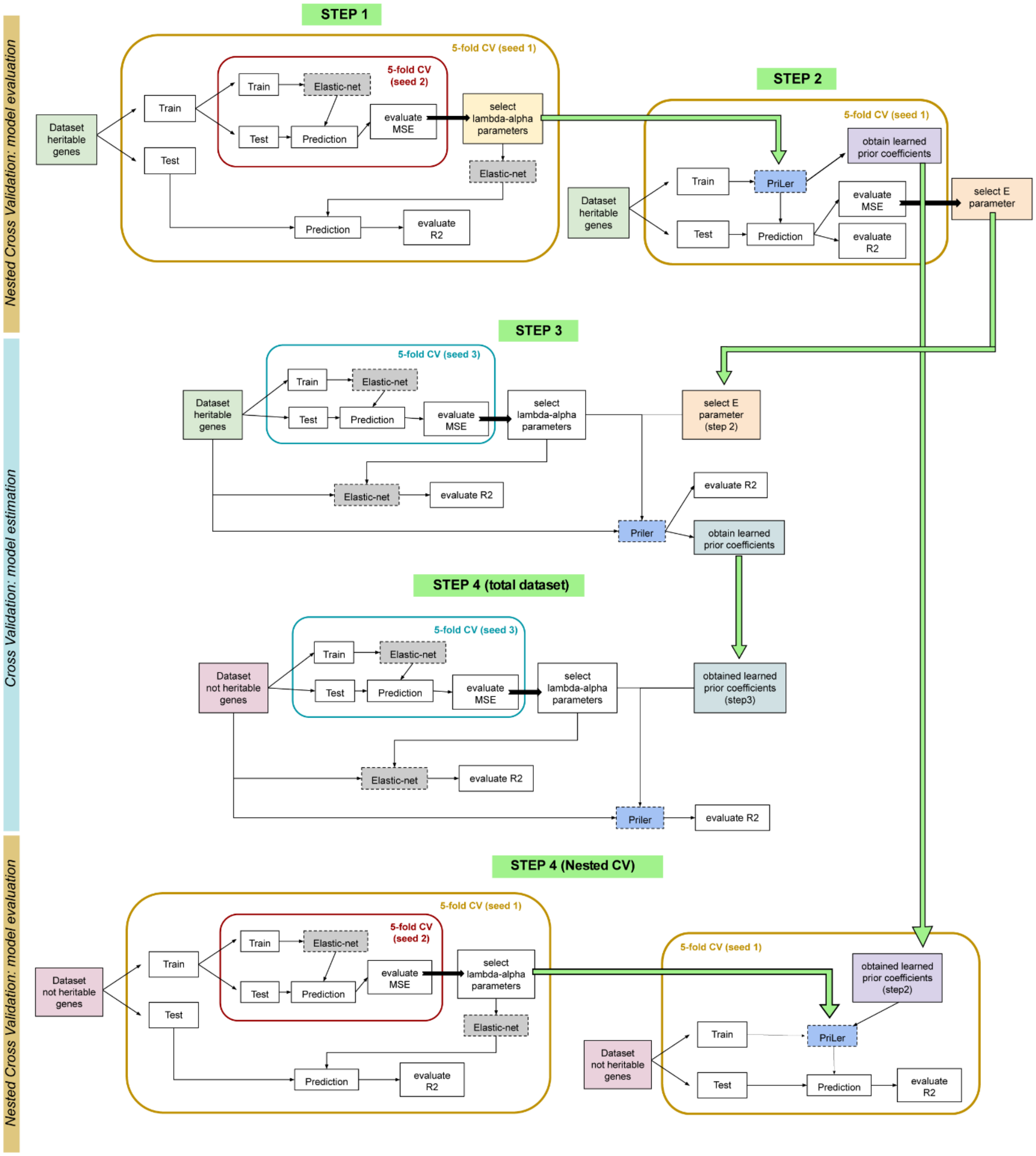

Supplementary Fig. 2. PriLer steps to model gene expression integrating prior variant knowledge.

The first step considers only heritable genes and builds an elastic-net regression model for each gene in a nested cross validation setting without prior information. The second step uses the optimal gene specific α-λ parameters combination found in step 1 to build a PriLer model in the

same nested cross-validation frame in order to find the optimal E parameter that controls the magnitude of the prior weights. Step 1 and 2 are also used to evaluate the prediction models based on R^2_{cv}. The third step finds the optimal α-λ parameter on the entire set (single cross validation) from elastic-net regression and uses these α-λ pairs together with optimal E parameter from step 2 to build PriLer models on the entire dataset. The fourth step considers only genes not heritable, repeats step 3 but instead of deriving prior coefficients, it uses the ones computed from heritable genes. Finally, in order to evaluate the models based on R^2_{cv} for genes not heritable, step 1 and 2 are also repeated with CV-specific prior coefficients derived in step 2.

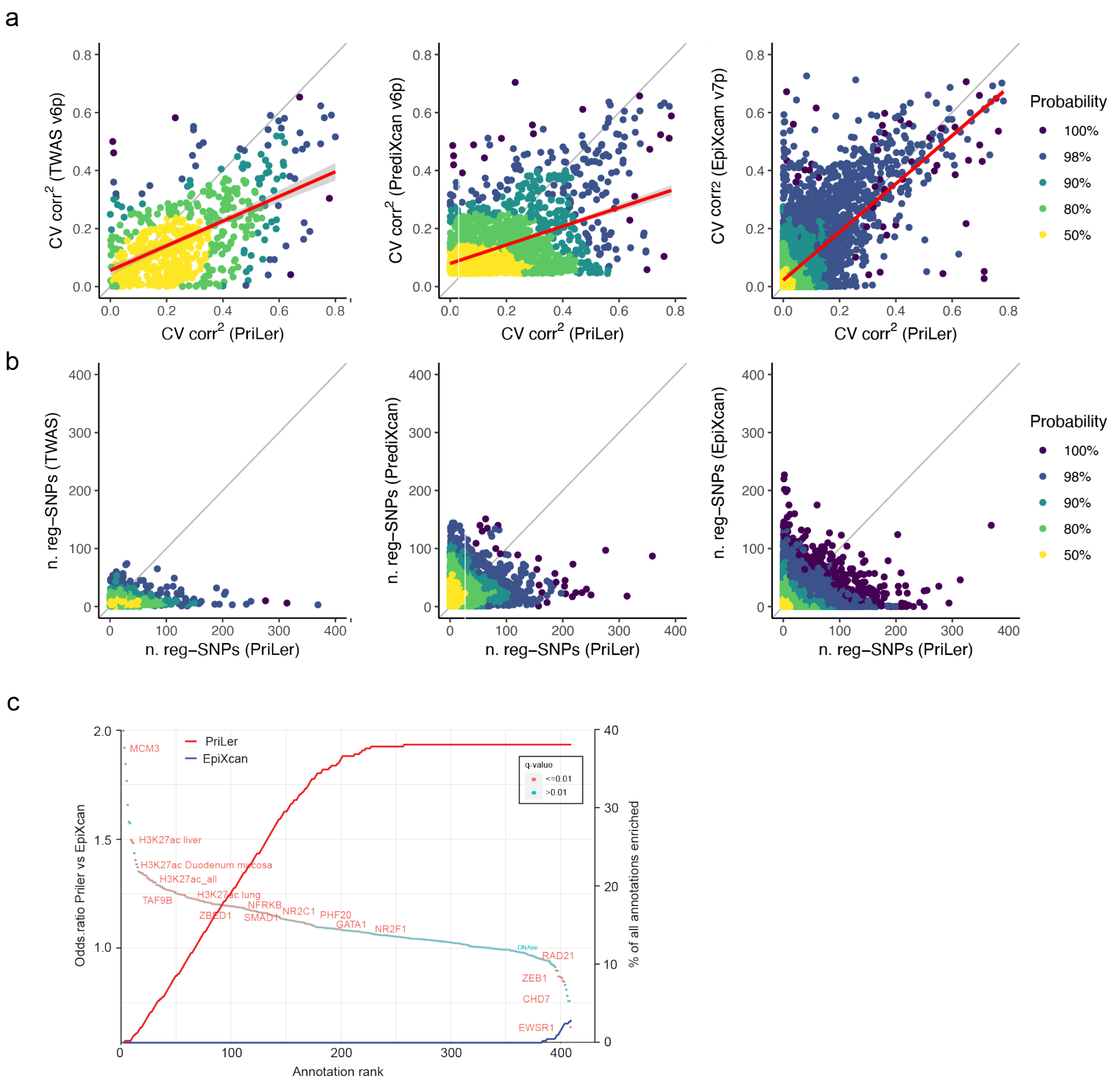

Supplementary Fig. 3. Comparison PriLer with FUSION, PrediXcan and EpiXcan

a. Comparison of prediction performance of imputed gene expression levels measured as squared correlation between adjusted gene expression and predicted expression across all cross-validation test folds (Online Methods) for PriLer (x-axis) to FUSION and PrediXcan build on GTEx v6p and EpiXcan on GTEx v7p (y-axis) for all common genes based on the liver gene expression model. Color indicates the density of observations and red line indicates linear regression line.

b. Comparison of number of regulatory SNPs ($\beta_{enet}\neq 0$) for common genes used by PriLer (x-axis), FUSION, PrediXcan and EpiXcan in liver tissue. Legend has the same meaning as in (a).

c. Comparison of biological feature enrichment of regulatory SNPs selected by PriLer or EpiXcan. Dots (red/blue) indicate enrichment (odds ratio PriLer vs. EpiXcan, y-axis left) of 410 biological annotation classes (H3K27ac and transcription factor binding sites in different tissues, Online Methods). Significance of enrichment was assessed by Fisher's exact test with p-value correction

using the BH-method. Biological annotations are ordered by enrichment rank (x-axis). Red line (PriLer) and blue line (EpiXcan) indicate percentage of significantly enriched biological annotations between PriLer vs EpiXcan (y-axis right) as a function of enrichment rank.

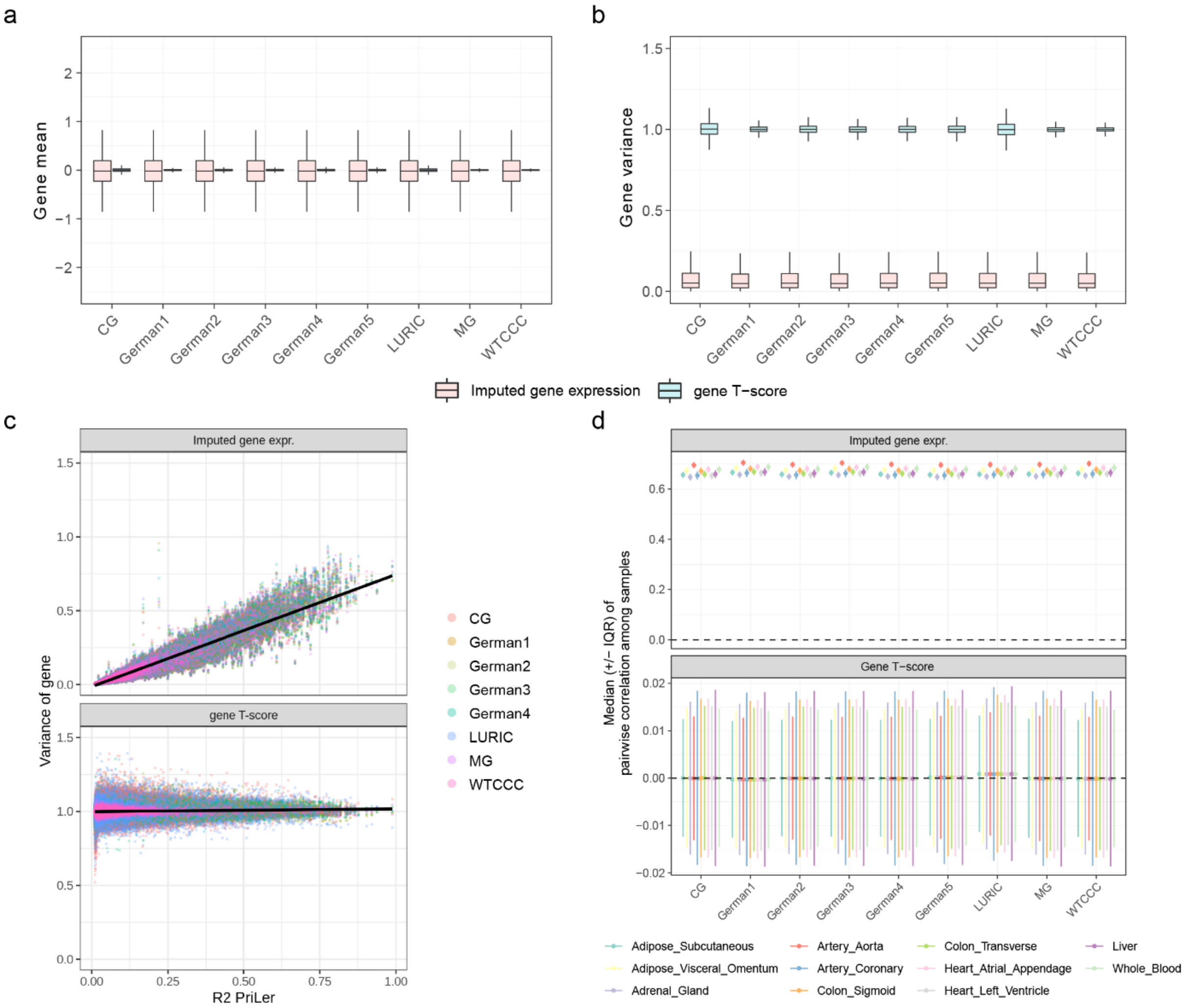

Supplementary Fig. 4: Gene T-score transformation reduces sample correlation and ensures similar expression distribution for each gene.

- **a.** For each CARDIoGRAM cohort (x-axis) average gene value (imputed expression or gene T-scores) across all samples and all tissues.
- **b.** Same as a. but y-axis indicates gene variances.
- **c.** Relationship between overall R^2 from PriLer gene models and gene variances across samples from imputed gene expression (top) or gene T-scores (bottom). Each dot refers to a gene in a tissue in a cohort (dot color). Black line is the regression line.

d. For each tissue and each CARDIoGRAM cohort, y-axis shows the median of samples pairwise correlations (error bars indicates 1st and 3rd quartiles), computed using imputed gene expression (top) or gene T-scores (bottom).

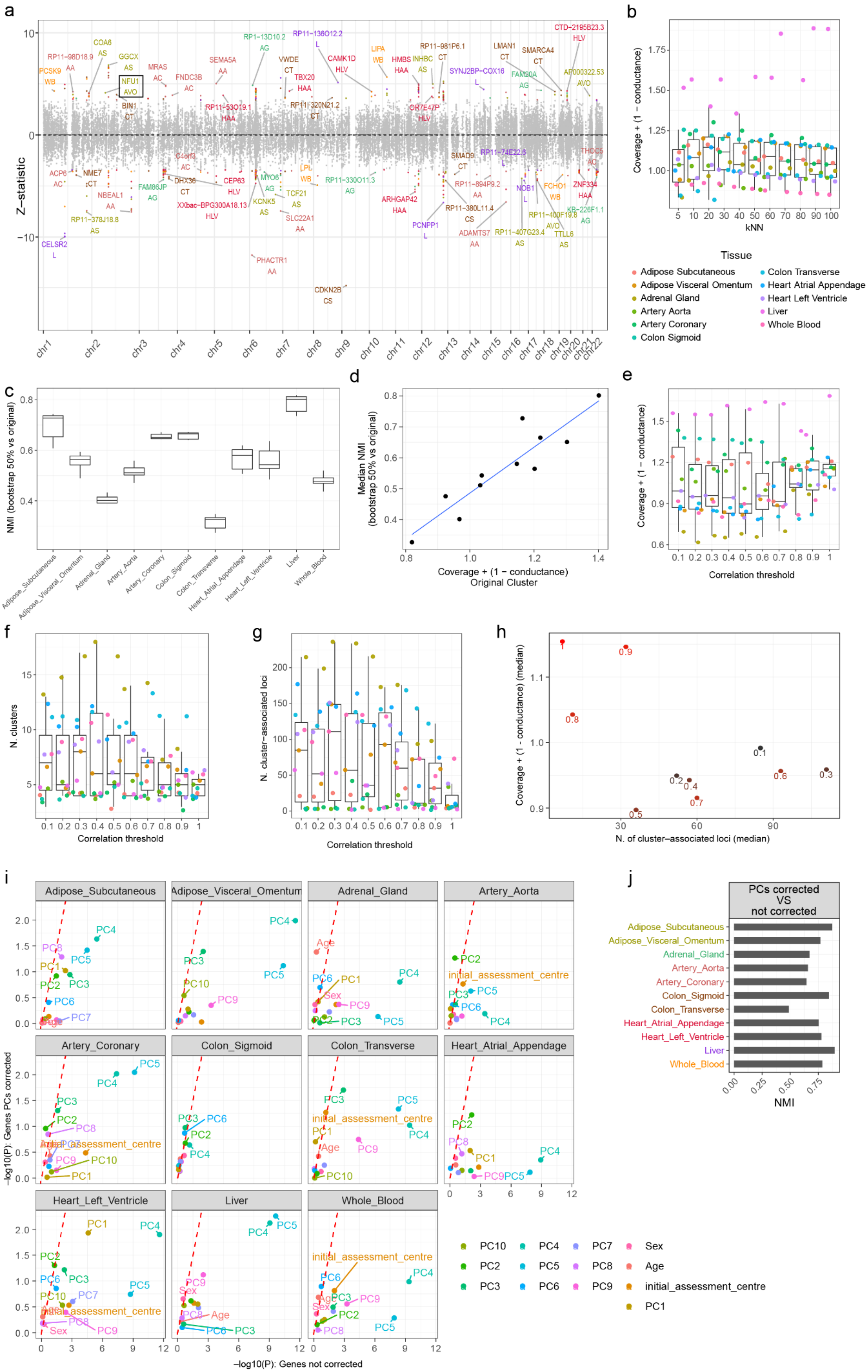
a
Z−statistic
b
Coverage + (1 − conductance)
kNN
Tissue
Adipose Subcutaneous
Adipose Visceral Omentum
Adrenal Gland
Artery Aorta
Artery Coronary
Colon Sigmoid
Colon Transverse
Heart Atrial Appendage
Heart Left Ventricle
Liver
Whole Blood
c
NMI (bootstrap 50% vs original)
d
Median NMI (bootstrap 50% vs original)
Coverage + (1 − conductance) Original Cluster
e
Coverage + (1 − conductance)
Correlation threshold
f
N. clusters
Correlation threshold
g
N. cluster−associated loci
Correlation threshold
h
Coverage + (1 − conductance) (median)
N. of cluster−associated loci (median)
i
−log10(P): Genes PCs corrected
−log10(P): Genes not corrected
PC10
PC2
PC3
PC4
PC5
PC6
PC7
PC8
PC9
Sex
Age
initial_assessment_centre
PC1
j
PCs corrected VS not corrected
NMI

Supplementary Fig. 5: Selection of kNN and correlation threshold for clustering.

a. Manhattan plot showing Z-statistic across 11 tissues, colored dots refer to genes with tissue specific associations (FDR ≤ 0.05). Acronyms in parenthesis indicate the initials of the tissue considered (AS = Adipose Subcutaneous, AVO = Adipose Visceral Omentum, AG = Adrenal Gland, AA = Artery Aorta, AC = Artery Coronary, CS = Colon Sigmoid, CT = Colon Transverse, HAA = Heart Atrial Appendage, HLV = Heart Left Ventricle, L = Liver, WB = Whole Blood).

b. Optimization of kNN parameter (x-axis) in CAD UKBiobank clustering, based on cluster coverage +1-conducantce (y-axis) as benchmarking parameter. Each dot refers to a result in one tissue.

c. For kNN = 20, NMI between clustering of bootstrapped 50% of the CAD samples and the original clustering (considering only samples in common), each dot refers to one of the 10 repetitions.

d. For kNN = 20, x-axis shows the cluster quality (all samples), y-axis shows the median of the NMI between clustering on 50% bootstrapped samples and the actual clustering on all samples. Each dot refers to a tissue and the blue line indicates the fitted linear relationship.

e. Evaluation of cluster quality as a function of correlation threshold (corr. thr.) for clumping (x-axis) based on coverage +1-conducance (y-axis)

f. Based on number of detected groups (y-axis) or

g. Based on the number of cluster-associated loci (in the tissue considered for clustering, y-axis).

h. Median of cluster associated loci (across tissues, x-axis) and median of cluster quality (across tissues, y-axis). Each dot refers to a possible corr. thr. Value also indicated in the color code.

i. $\log_{10}$(p-value) testing association of clustering results with PCs from 1 to 10 and Age (Kruskal-Wallis test) and with Sex and Assessment Centre (χ^2 test) before (x-axis) and after (y-axis) correction of gene T-scores for PCs. Red dashed line represents the intercept.

j. NMI between clustering results using genes corrected for PCs or uncorrected.

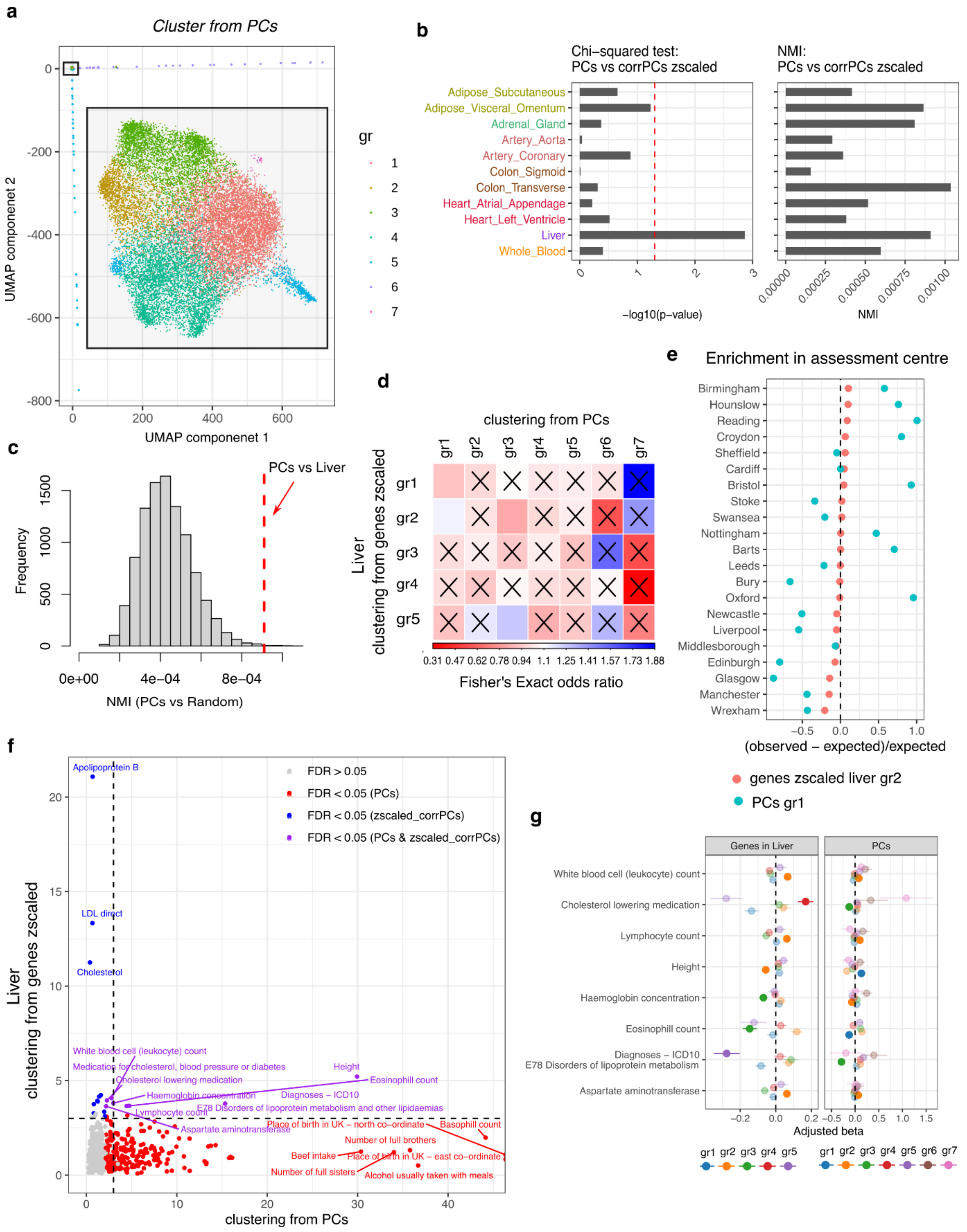

Supplementary Fig. 5. Comparison of CAD clustering structure based on T-scores in liver and based on Principal Components from genotype data in UKBB.

a. CAD cases are clustered using the first 40 PCs from UKBB (standardized). UMAP based on PCs, where the color refers to the assigned PC clustering.

b. The comparison between PC clustering and gene T-score grouping is shown in terms of -log10 p-value of a chi-squared test (left panel) and NMI (right panel). The dashed line refers to the nominal p-value of 0.05, and the comparison is shown for each tissue.

c. The histogram of NMI between the cluster from PCs and 10,000 randomly assigned groups with the same size as liver clustering is shown. The dashed line refers to the NMI comparing PCs and the actual liver clustering.

d. The results of pairwise Fisher's Exact tests between a group detected in the PC clustering (columns) and a group detected in liver clustering (rows) are presented in a heatmap that indicates the computed odds ratio. Non-significant results at the nominal level of 0.01 are highlighted with an "x".

e. Investigation of enrichment in the assessment center for groups 2 in liver and 1 in PC clustering. The x-axis indicates the fraction of (observed - expected)/expected counts as computed from the chi-squared statistic across the centers versus a group assignment (gr_i or not gr_i), while the y-axis indicates the center assignment.

f. The results of testing each endophenotype are presented in dots that indicate the -log10 p-value of the most significant group-specific difference in PCs (x-axis) and liver (y-axis) clustering. The dashed lines refer to p-value = 0.001, and the color reflects the FDR significance threshold.

g. Forest plot of the group-specific differences for the eight endophenotypes that are significant in both PCs and liver clustering. The x-axis shows the regression coefficient from a GLM test of gr_i vs all remaining samples with 95% CI. The dots that are not shaded indicate the groups with the most significant association in terms of p-value.

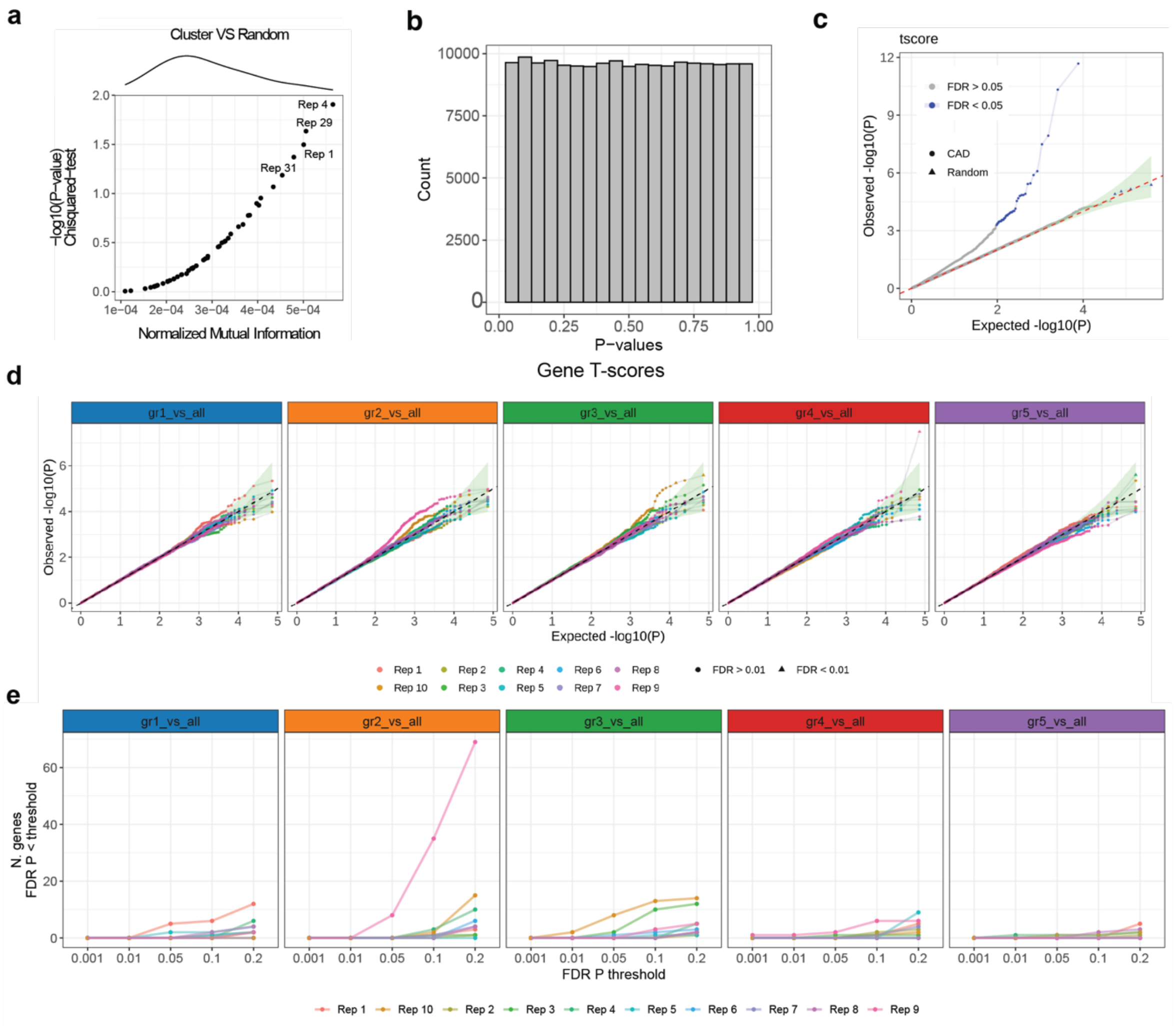

Supplementary Fig. 6 Genes/pathways and endophenotype associations with random clustering structure

a. Random clustering of CAD patients maintaining the same group size as liver grouping. X-axis NMI (x-axis) and -log10 p-values (y-axis) from χ2 statistics between liver clustering and random groupings.

b. Count of p-values in specific intervals for all gene associations with random clusters.

c. Expected and observed p-value distributions for CAD (represented by dots) and random phenotypes (represented by triangles) for gene scores. The diagonal line represents the

expected distribution and the green shaded area shows the 95% confidence interval from a beta distribution. Blue points indicate genes that are significant at a 0.05 false discovery rate level, corrected separately for CAD and each simulation.

d. Quantile-quantile representation of the association between gene T-scores and group-wise specific (gri vs all remaining) testing, using the WMW method across all tissues. The expected p-values follow a uniform distribution, and the plot shows a dashed line along the diagonal, with a shaded green area representing a 95% confidence interval from the beta distribution. Each line in the plot corresponds to one of the 10 simulations.

e. Number of significant associations that passed the FDR threshold for each of the 10 random clustering experiments as a function of varying FDR levels on the x-axis.

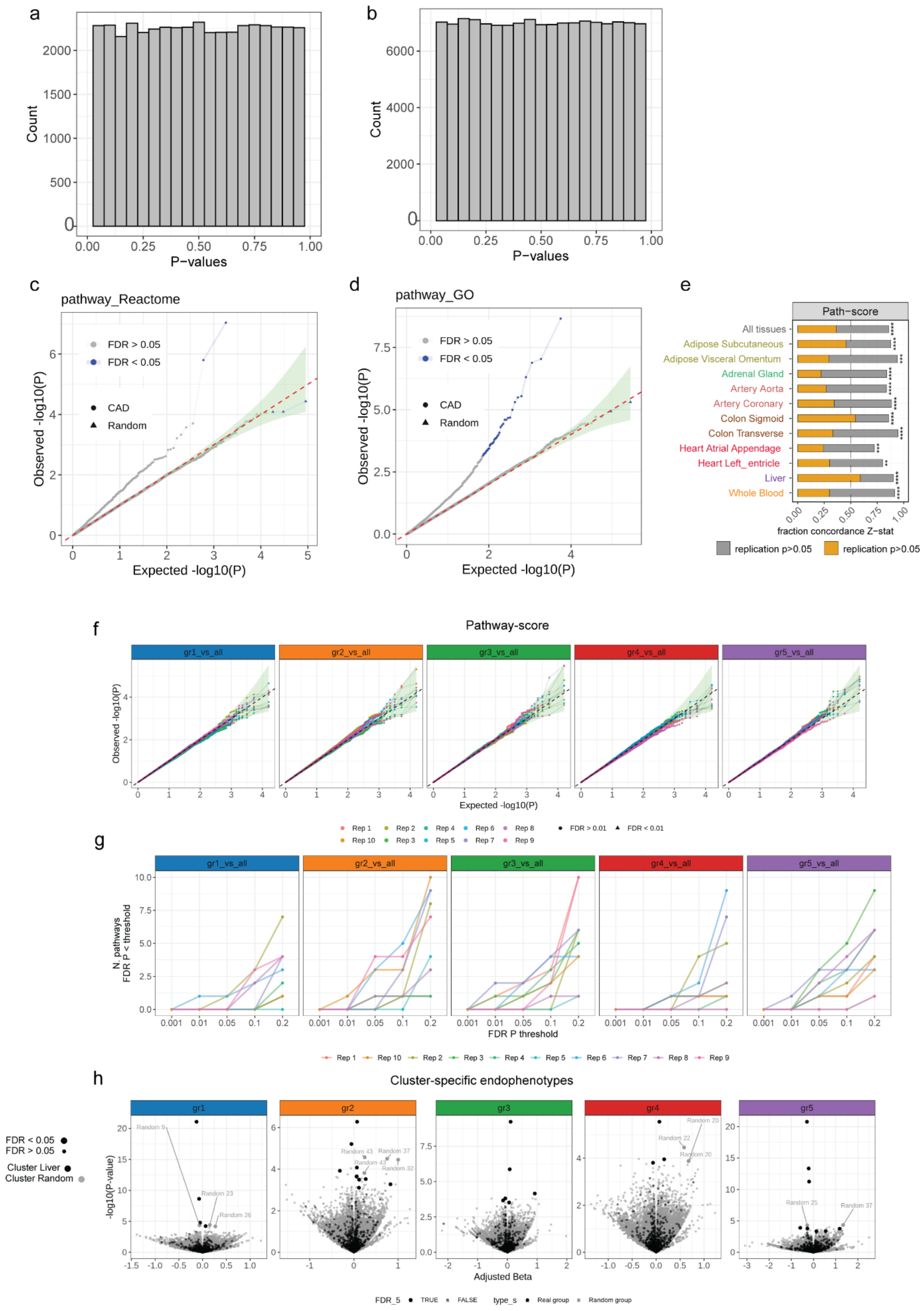
a
b
Count
P−values
c
pathway_Reactome
FDR > 0.05
FDR < 0.05
CAD
Random
Observed -log10(P)
Expected -log10(P)
d
pathway_GO
e
Path−score
All tissues
Adipose Subcutaneous
Adipose Visceral Omentum
Adrenal Gland
Artery Aorta
Artery Coronary
Colon Sigmoid
Colon Transverse
Heart Atrial Appendage
Heart Left_entricle
Liver
Whole Blood
fraction concordance Z−stat
replication p>0.05
replication p>0.05
f
Pathway-score
gr1_vs_all
gr2_vs_all
gr3_vs_all
gr4_vs_all
gr5_vs_all
FDR > 0.01
FDR < 0.01
g
N. pathways FDR P < threshold
FDR P threshold
h
Cluster-specific endophenotypes
FDR < 0.05
FDR > 0.05
Cluster Liver
Cluster Random
-log10(P-value)
Adjusted Beta
FDR_5
TRUE
FALSE
type_s
Real group
Random group

Supplementary Fig. 7: P-value calibration for TWAS and PALAS in whole blood

a. Distribution of p-values for PALAS on random phenotypes in whole blood across 50 simulations, using size matched cases/control and the age/sex distributions. Count of p-values in specific intervals for Reactome pathway-scores,

b. Same as a. for GO pathway-scores

c. Expected and observed p-value distributions for CAD (represented by dots) and random phenotypes (represented by triangles) for Reactome pathway-scores. The diagonal line represents the expected distribution and the green shaded area shows the 95% confidence interval from a beta distribution. Blue points indicate genes that are significant at a 0.05 false discovery rate level, corrected separately for CAD and each simulation.

d. Same as c for GO pathway-scores.

e. Reproducibility of UKBB based pathway associations in external cohorts via meta-analysis of CARDIoGRAM cohorts. X-axis shows the fraction of significant pathways in UKBB that have the same effect sign (Z-statistic) in CARDIoGRAM meta-analysis, p-values are computed from one-sided sign test ($* = P \leq 0.05$, $** = P \leq 0.01$, $*** = P \leq 0.001$, $**** = P \leq 0.0001$). The fraction of pathways concordant and nominal at a p-value threshold of 0.05 is shown in the yellow bar.

f. Quantile-quantile representation of the association between pathway-scores and group-wise specific (gri vs all remaining) testing, using the WMW method across all tissues differences for selected gene-sets (Jaccard similarity ≤ 0.2). The expected p-values follow a uniform distribution, and the plot shows a dashed line along the diagonal, with a shaded green area representing a 95% confidence interval from the beta distribution. Each line in the plot corresponds to one of the 10 simulations.

g. Number of significant pathway associations associations that passed the FDR threshold for each of the 10 random clustering experiments as a function of varying FDR levels on the x-axis.

h. Volcano plot of cluster-specific endophenotype differences. The x-axis represents the β regression coefficient from the GLM, which refers to the features of gr_i versus the remaining cases, while the y-axis shows the corresponding -log10 p-value. Each grey dot represents an endophenotype among the 637 UK Biobank phenotypes that was tested for a random clustering configuration out of 50 repetitions. In contrast, each black dot refers to the endophenotype testing on the actual liver clustering. In both cases, the size of the dots corresponds to the significance of the test after correction (FDR ≤ 0.05).

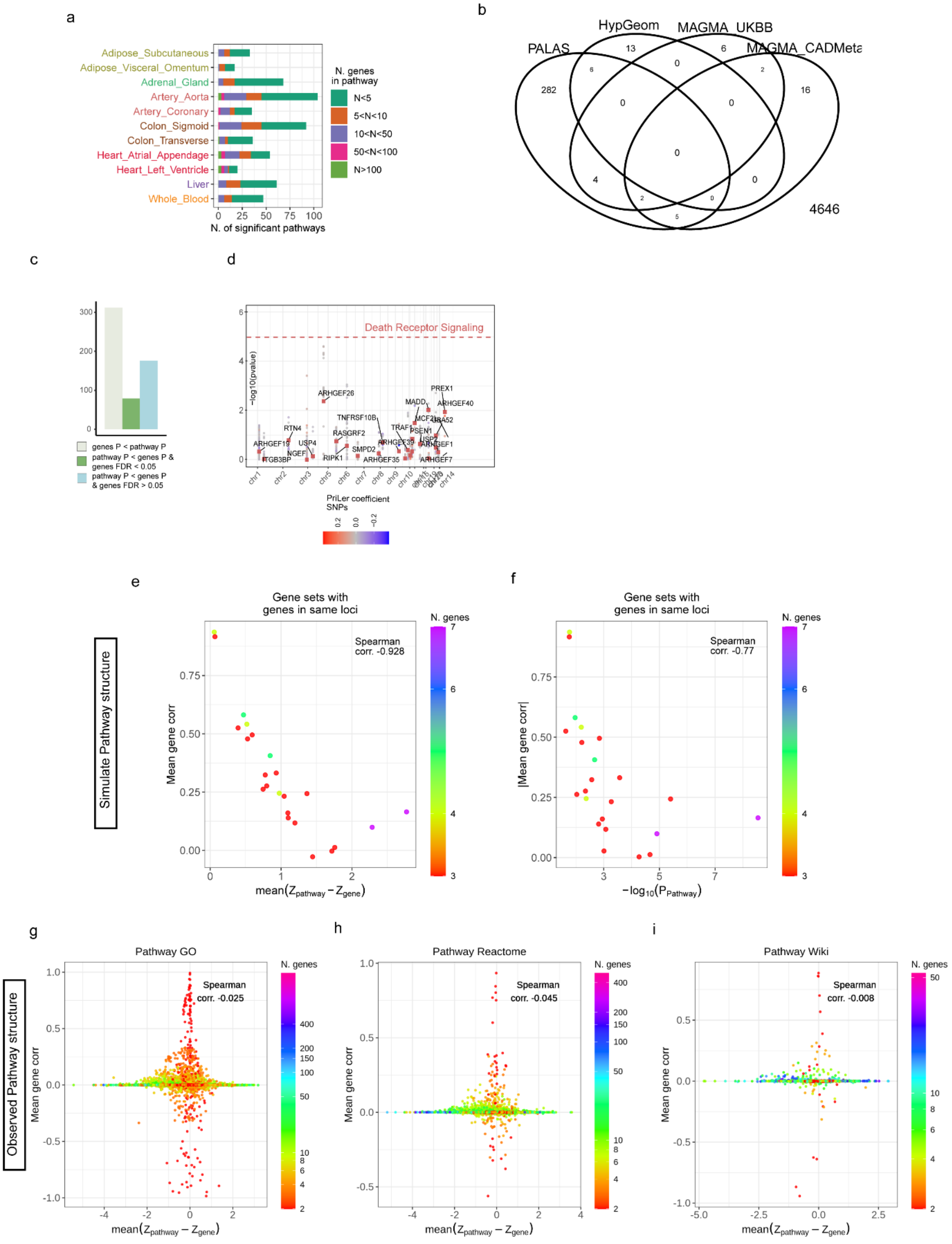

Supplementary Fig. 8. PALAS summary results for CAD and relevance of gene correlation

a. Significant pathways linked to CAD in each tissue with tissue-specific FDR of less than or equal to 0.05. The bars represent the number of pathways and their color represents the number of genes that are reliably predicted in that tissue (based on T-score genes).

b. Venn-diagram of PALAS significant pathways (FDR 0.05), pathways significant from hyper-geometric test (FDR 0.05) using TWAS significant genes and pathways significant from MAGMA (FDR 0.05) applied to GWAS performed on matched UK Biobank data (MAGMA_UKBB) or GWAS summary statistics from CAD GWAS in [20].

c. Number of significant pathways (PALAS FDR $\leq$ 0.05) with at least one gene reaching better significance than the pathway (ivory), with all genes in the pathway less significant but with at least one gene having TWAS FDR $\leq$ 0.05 (green), and all genes less significant and not passing TWAS FDR 0.05 threshold (light blue).

d. Reactome Death Receptor Signaling in artery aorta. The pathway significance is indicated by the dashed horizontal line, the coloured squares show genes included in that pathway and the corresponding TWAS p-value (y-axis) and the dots indicate the matched GWAS p-value of SNPs regulating those genes with colour reflecting PriLer regulatory coefficients.

e. This figure shows the simulation of pathway structure using gene expression data in whole blood from the same locus with a concordant effect size sign and TWAS nominal p-value of less than 0.1. The simulation covers 46 pathways in total. Each simulated pathway is a point with a color indicating the number of genes in the pathway. The X-axis represents the average difference in Z-statistic between the pathway and the included genes, while the Y-axis shows the absolute value of the mean correlation among the genes in the pathway.

f. Similar to e., but with the X-axis showing -log 10 p-value from PALAS.

g. Improvement in pathway significance related to the individual gene level p-values based on TWAS that are included in it as a function of the correlation between the genes (y-axis). Each point in the figure represents a pathway from GO

h. Reactome or

i. WikiPathways with the color code shows the number of genes that were used to calculate the pathway-score. The X-axis displays the average difference in Z-statistics between a pathway and its corresponding genes, and the Y-axis shows the mean correlation between those genes.

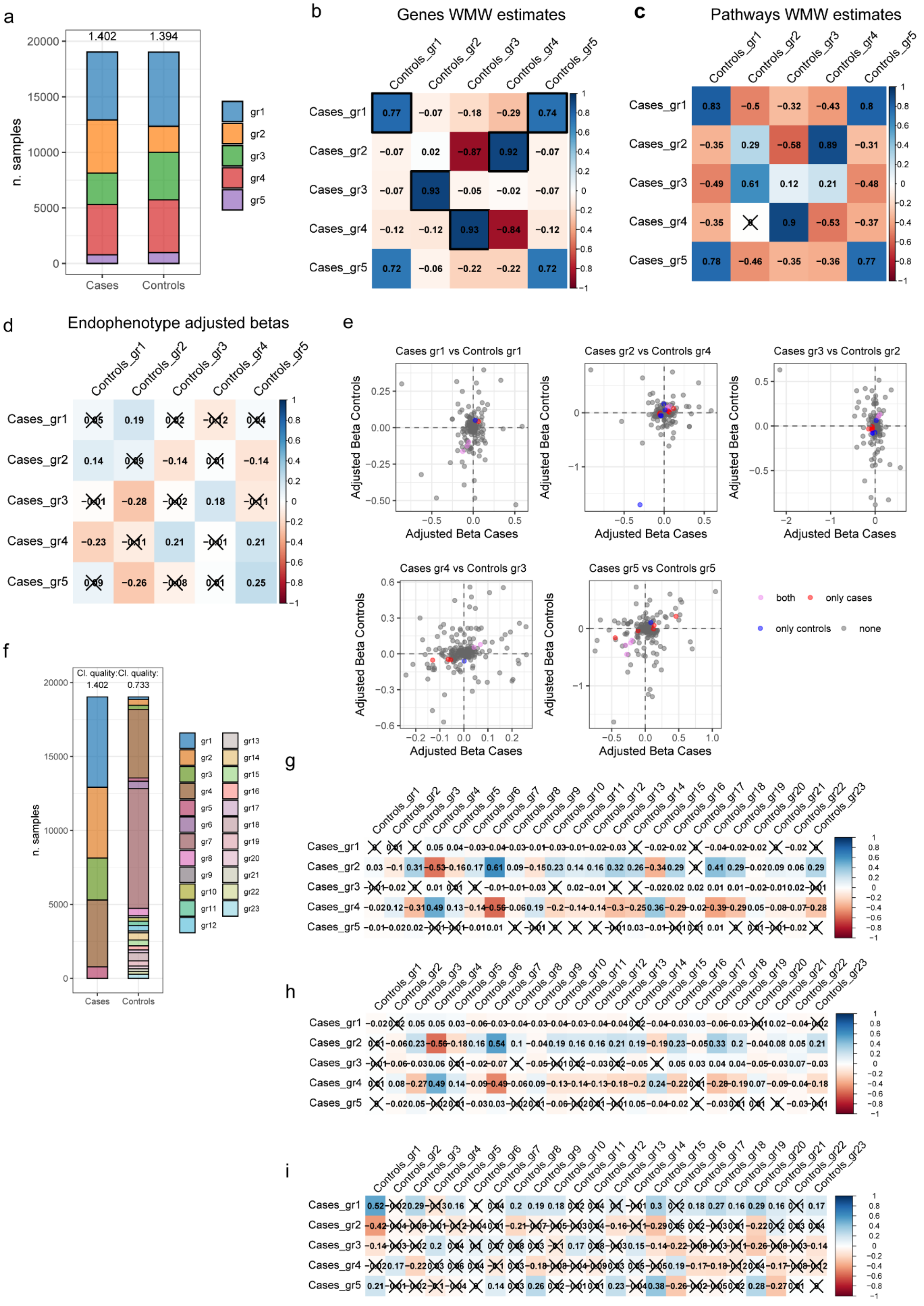
a
1.402
1.394
n. samples
Cases
Controls
gr1
gr2
gr3
gr4
gr5
b
Genes WMW estimates
Controls_gr1
Controls_gr2
Controls_gr3
Controls_gr4
Controls_gr5
Cases_gr1 0.77 −0.07 −0.18 −0.29 0.74
Cases_gr2 −0.07 0.02 −0.87 0.92 −0.07
Cases_gr3 −0.07 0.93 −0.05 −0.02 −0.07
Cases_gr4 −0.12 −0.12 0.93 −0.84 −0.12
Cases_gr5 0.72 −0.06 −0.22 −0.22 0.72
c
Pathways WMW estimates
Cases_gr1 0.83 −0.5 −0.32 −0.43 0.8
Cases_gr2 −0.35 0.29 −0.58 0.89 −0.31
Cases_gr3 −0.49 0.61 0.12 0.21 −0.48
Cases_gr4 −0.35 0.9 −0.53 −0.37
Cases_gr5 0.78 −0.46 −0.35 −0.36 0.77
d
Endophenotype adjusted betas
Cases_gr1 0.19
Cases_gr2 0.14 −0.14 −0.14
Cases_gr3 −0.28 0.18
Cases_gr4 −0.23 0.21 0.21
Cases_gr5 −0.26 0.25
e
Cases gr1 vs Controls gr1
Cases gr2 vs Controls gr4
Cases gr3 vs Controls gr2
Cases gr4 vs Controls gr3
Cases gr5 vs Controls gr5
Adjusted Beta Controls
Adjusted Beta Cases
both
only cases
only controls
none
f
Cl. quality: 1.402
Cl. quality: 0.733
n. samples
Cases
Controls
g
h
i

Supplementary Fig. 9: Clustering of non-affected individuals.

Clustering of non-affected individuals from UKBB in liver, matched to CAD cases by age and sex.

a. N. of samples per cluster across cases and controls partition. The number on top of indicates the cluster quality (coverage + 1 – conductance).

b. Pearson correlation of WMW estimates from cluster-specific genes for groups of cases (rows) and groups of controls (column). Squared with black edges indicate the most likely control – cases group matching based on the highest correlation.

c. Same as b. but considering WMW estimates from cluster-specific pathways. Squares with X indicate that the correlation is not significant at nominal level 0.05.

d. Venn-diagram of unique cluster-specific genes (top) and pathways (bottom) between cases and controls partitions.

e. Y-axis indicates the absolute value of WMW estimates for cluster-specific genes separated per genes unique to the cases/controls partition or in common between the two, in cases clustering (left) or controls clustering (right).

f. same as e. but considering cluster-specific pathways.

g. Venn diagram of unique cluster-specific endophenotypes associations in cases (red) and controls (blue).

h. Pearson correlation of β_{GLM} from cluster-specific endophenotypes analysis for groups of cases (rows) and groups of controls (column). Squares with X indicate that the correlation is not significant at the nominal level 0.05.

i. For the matching cases – controls group (indicated as a title and based on genes correlation), scatterplot of β_{GLM} from cluster-specific endophenotypes analysis. The dot color indicates

whether the endophenotype is significant (FDR <= 0.1) in both, only in cases group or only in controls group.

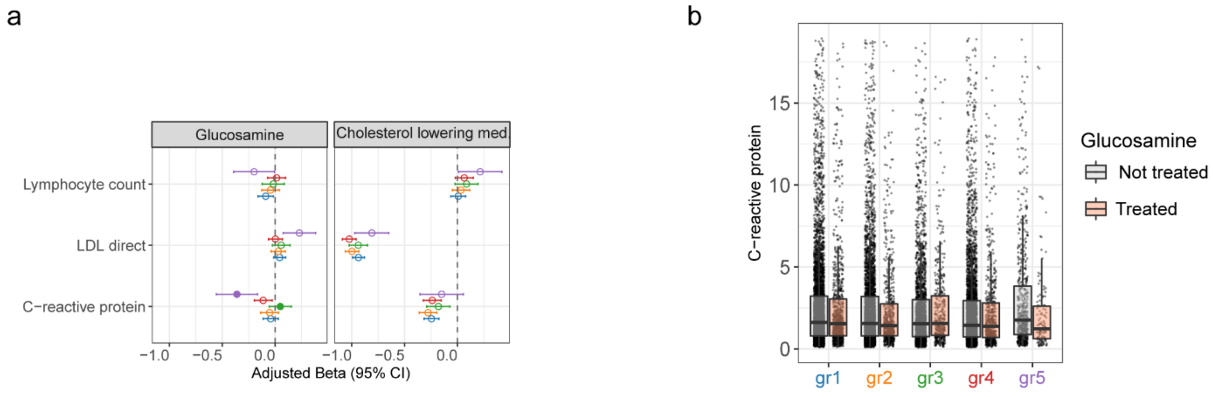

Supplementary Fig. 10 Endophenotype and treatment response analyses on CAD clustering

a. Treatment response showing the effect of glucosamine and cholesterol-lowering medications in each group for selected phenotypes. X-axis shows regression coefficient with 95% CI from GLM in each group, full dots indicate groups that are significantly different in a pairwise comparison after BH correction (pairwise comparison-specific and treatment-specific), tested using Z-test for comparing regression coefficients.

b. Distribution of original CRP values in each group when taking or not glucosamine supplements, y-axis is cropped at CRP=20 mg/L excluding 330 outliers. Boxplot elements include median as central line, 1st and 3rd quartiles as box limits, 1.5 interquartile ranges from 1st and 3rd quartiles as corresponding whiskers.

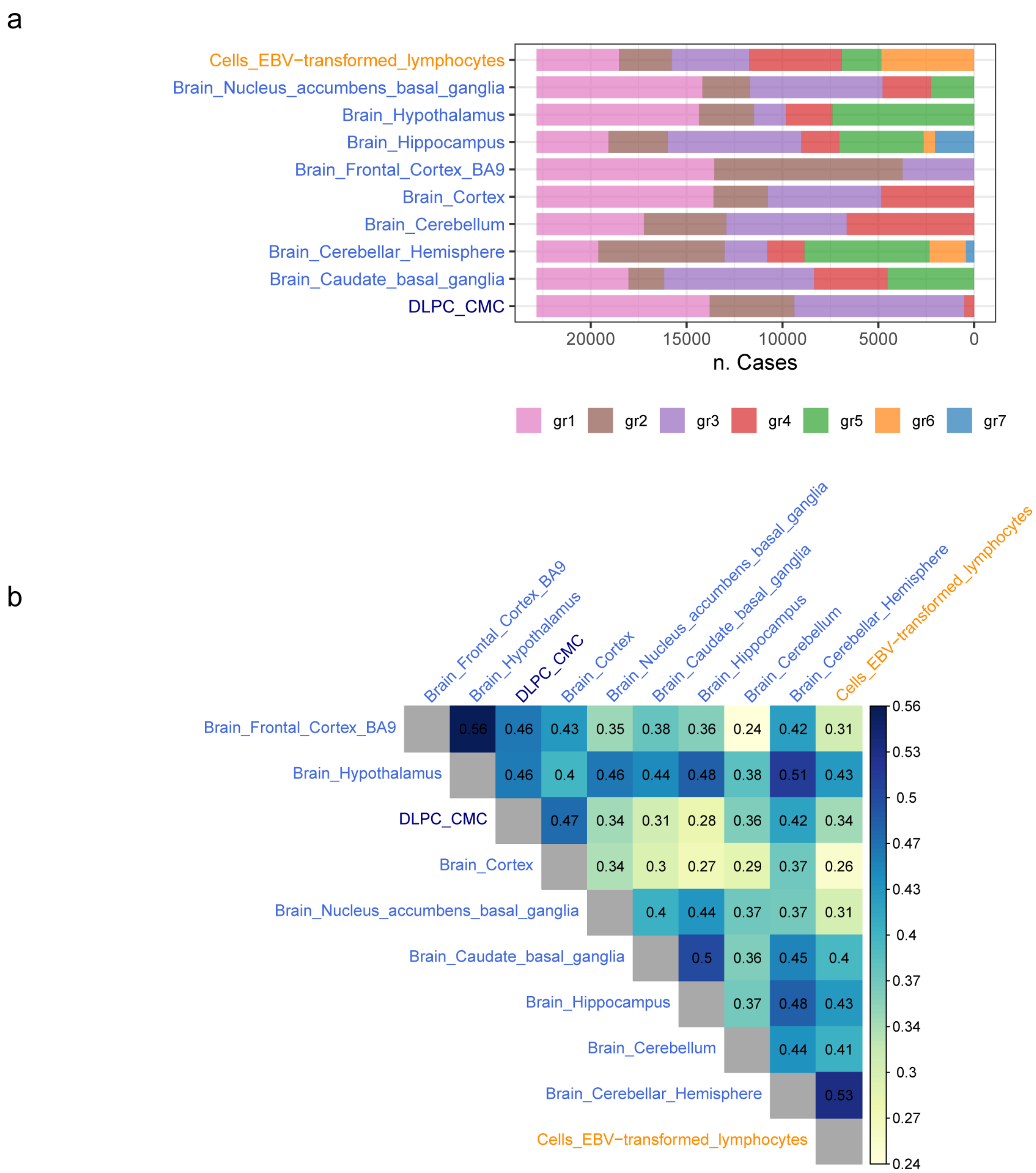

Supplementary Fig. 11. Comparison of clustering structure for SCZ patients across tissues.

a. Proportion of individuals in each tissue-specific cluster among 22,827 patients.

b. Normalized mutual information (NMI) for each pair of tissue-specific clustering structure.

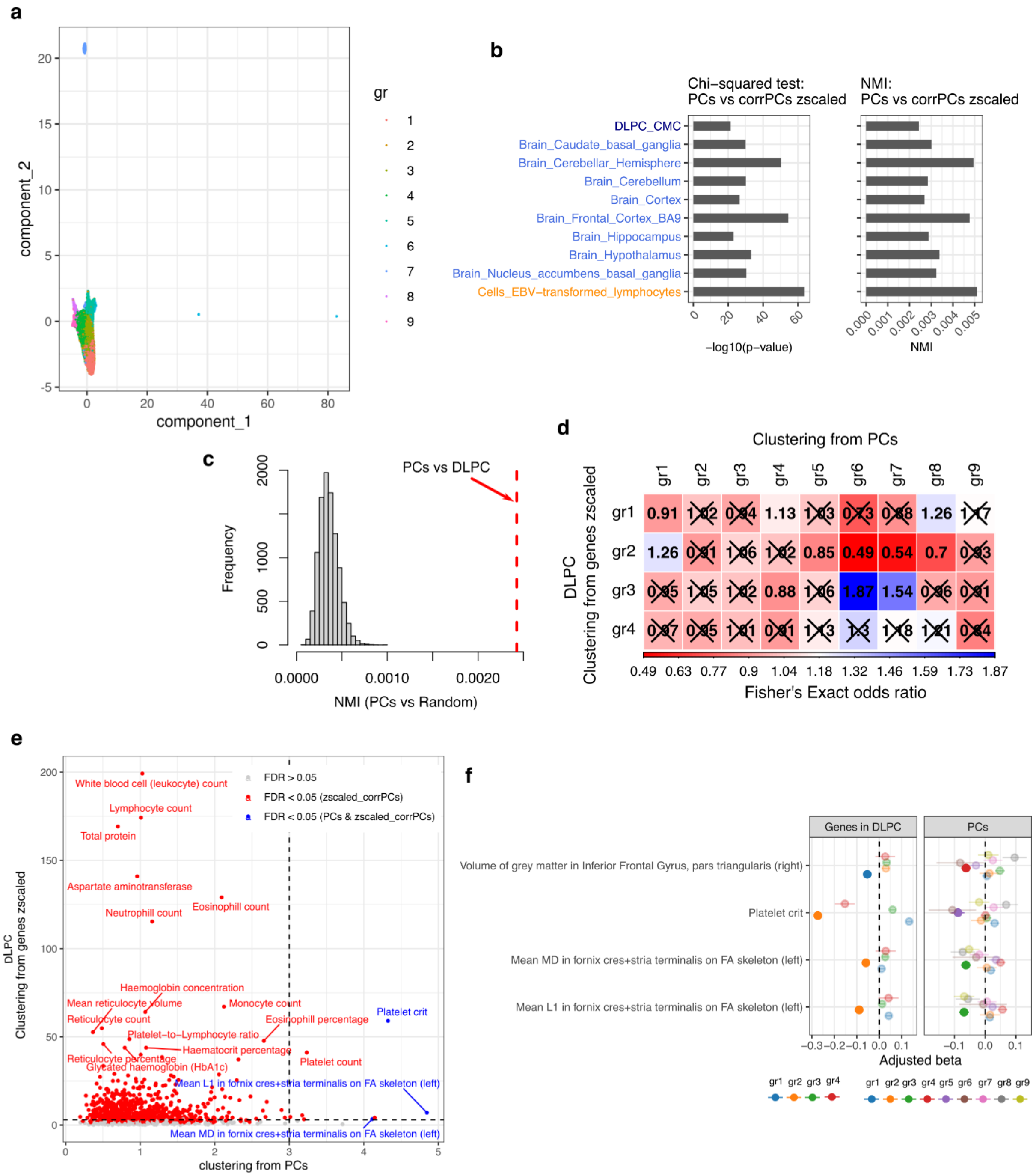

Supplementary Fig. 12: Comparison SCZ clustering structure based on DLPC and based on PCs.

SCZ cases clustered using the first 20 PGC PCs (standardized).

a. UMAP based on PCs, where the color refers to the assigned PC clustering.

b. For each tissue, comparison between PC clustering and gene T-score grouping is shown in terms of -log10 p-value of a chi-squared test (left panel) and NMI (right panel).

c. The histogram of NMI between the cluster from PCs and 10,000 randomly assigned groups with the same size as liver clustering is shown. The dashed line refers to the NMI comparing PCs and the actual DLPC clustering.

d. The results of pairwise Fisher's Exact tests between a group detected in PC clustering (columns) and a group detected in DLPC clustering (rows) are presented in a heatmap that indicates the computed odds ratio. Non-significant results at the nominal level of 0.01 are highlighted with an "x".

e. The results of testing each endophenotype are presented in dots that indicate the -log10 p-value of the most significant group-specific difference in PCs (x-axis) and DLPC (y-axis) clustering. The dashed lines refer to p-value = 0.001, and the color reflects the FDR significance threshold.

f. Forest plot of the group-specific differences for the endophenotypes that are significant in PCs cluster at FDR 0.1 threshold. The x-axis shows the regression coefficient from a GLM test of gr_i vs all remaining samples with 95% CI. The dots that are not shaded indicate the groups with the most significant association in terms of p-value.

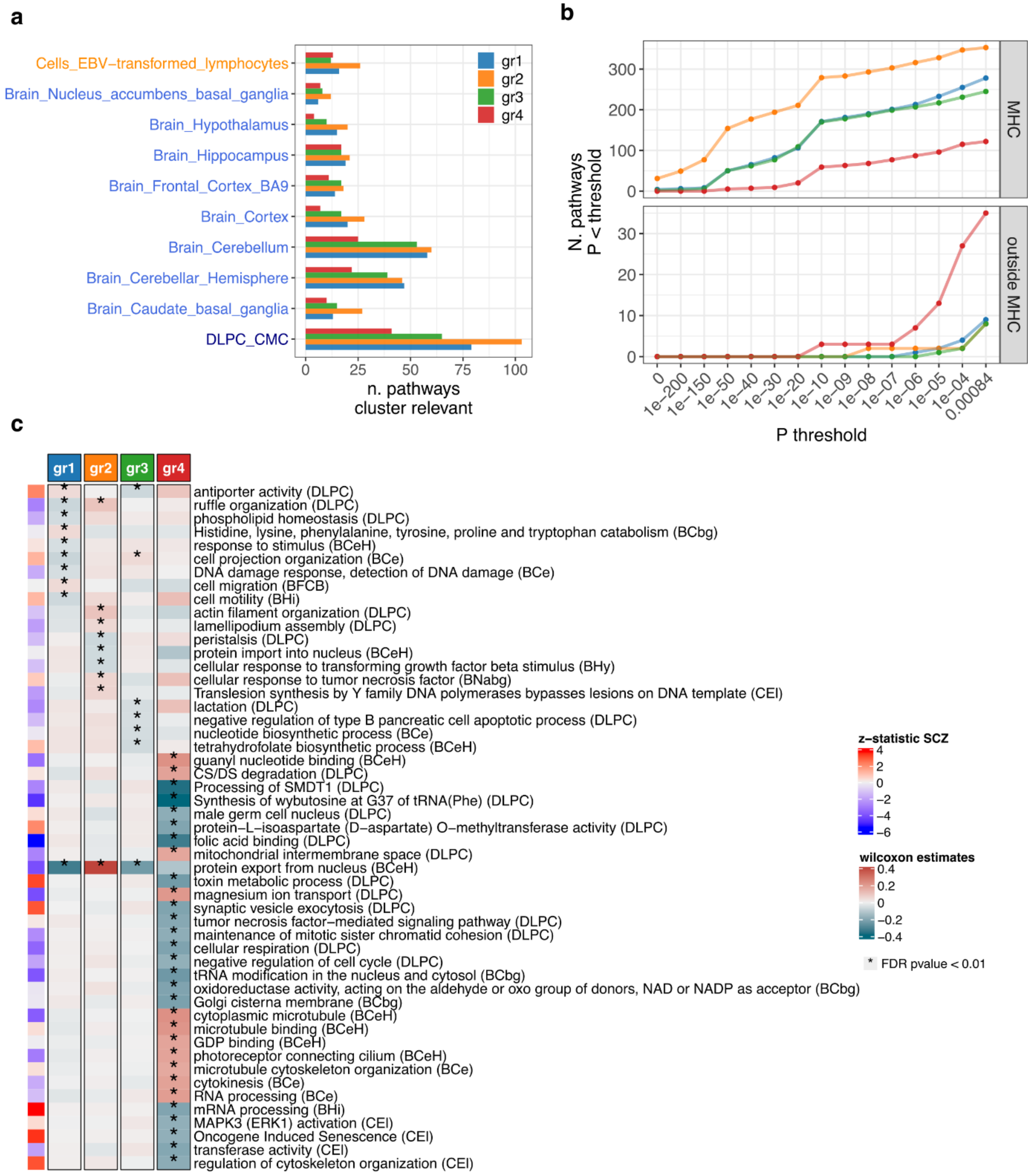

Supplementary Fig. 13: Pathways associated with SCZ clustering structure in DLPC.

a. The number of significant pathways (tissue specific FDR ≤ 0.01) associated with each group from the Wilcoxon-Mann-Whitney (WMW) test of group i versus the remaining patients. The pathways included in the analysis are from Reactome and GO and are filtered such that Jaccard

similarity ≤ 0.2, retaining only the pathways with the highest coverage and removing significant pathways having discordant WMW estimates across tissues.

b. For each group, the number of significant pathways passing the WMW p-value threshold is plotted on the y-axis against the p-value threshold on the x-axis, split into pathways that include at least one gene in the major histocompatibility complex (MHC) (top panel) and those that do not (bottom panel).

c. The WMW estimates for the significant pathways not including any genes in MHC (rows) are shown, testing each group against the rest (columns) and considering only the most significant tissue per pathway when repeated. The tissue tested is indicated by an acronym for the initial of the tissue name in parentheses. The row annotation on the left refers to the corresponding schizophrenia Z-statistics from the Psychiatric Genomics Consortium schizophrenia (SCZ) PGC2-PALAS dataset.

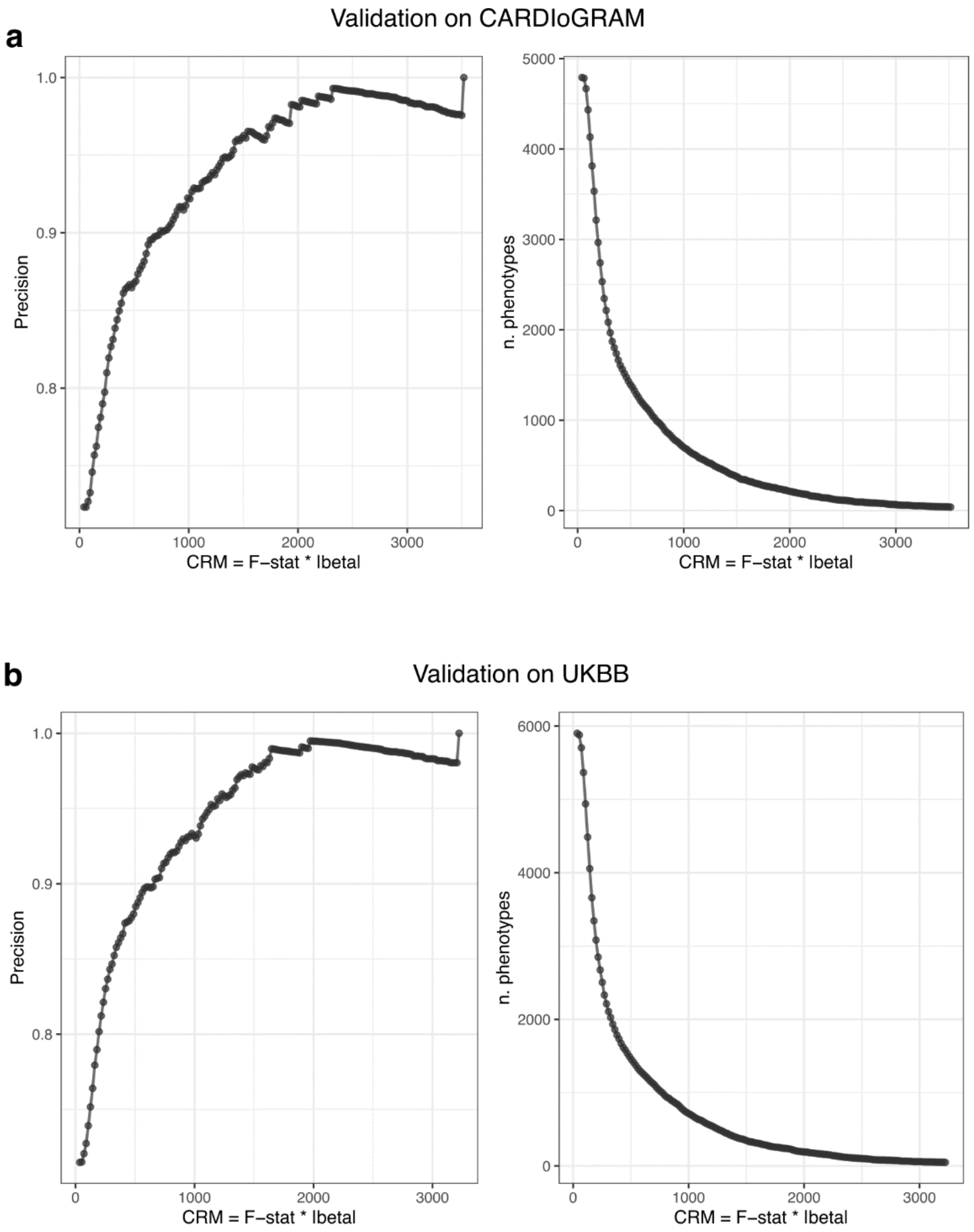

Supplementary Fig. 14. Evaluation of gene-RS performance in resembling actual endophenotypes

Validation of cluster-reliable measure (CRM) in CAD.

a. Meta-analysis of gene-RS differences built on 9 CARDIoGRAM cohorts or

b. UKBB. For the validation on UKBB, we included the same individuals used to build the original TWAS coefficients and to estimate R2. In both (a-b), CRM (x-axis) is compared to the actual

CAD endophenotypic differences detected in each tissue-specific clustering, combined all together. On the left panel, the y-axis indicates the precision computed as the fraction of group-specific gene-RS differences having the same sign of GLM regression coefficient in the actual CAD endophenotype analysis, among all the endophenotypes passing CRM threshold. On the right panel, the y-axis indicates the number of phenotypes passing that CRM threshold.

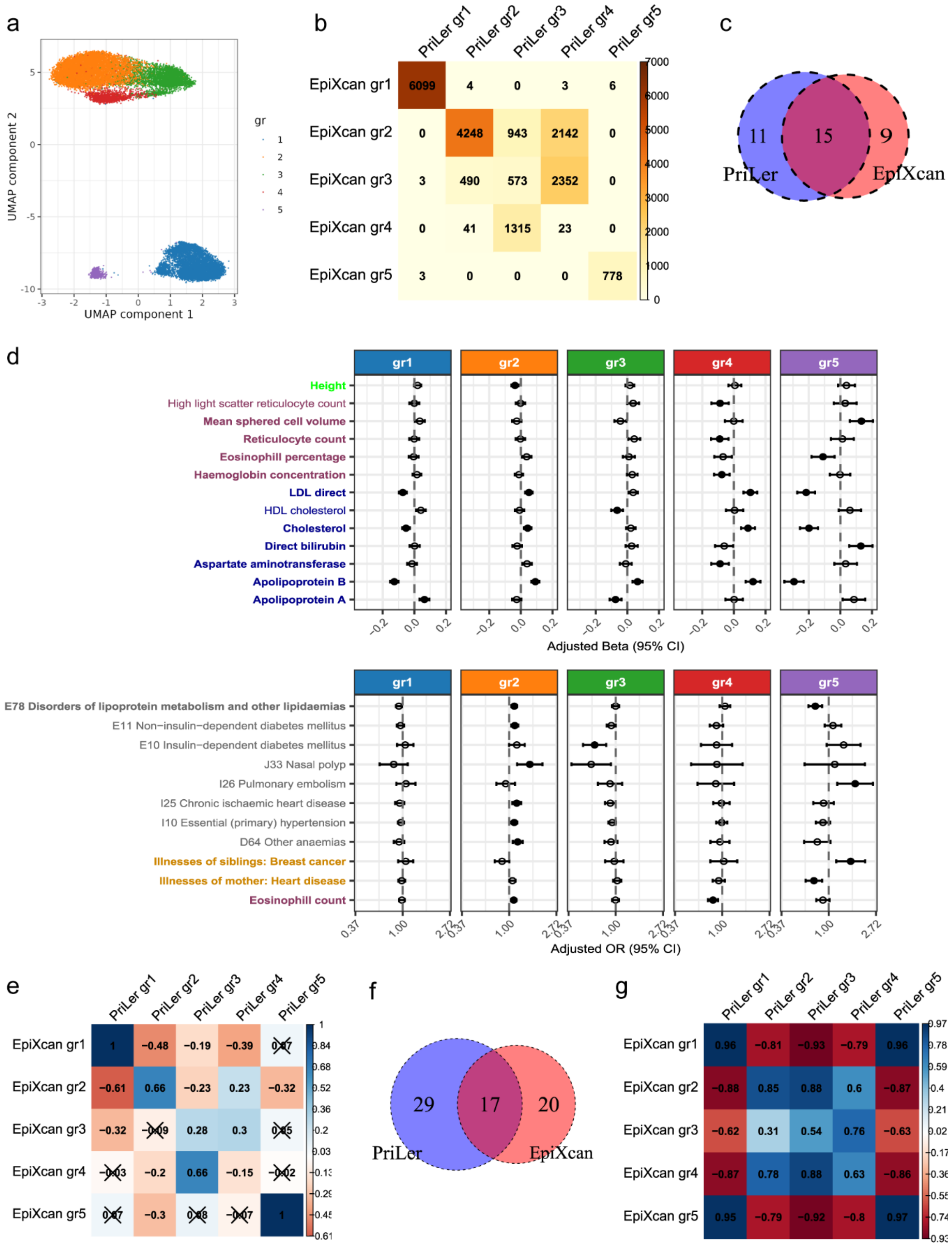

a
b
c
d
e
f
g
UMAP component 1
UMAP component 2
gr
PriLer
EpiXcan
11
15
9
29
17
20
Adjusted Beta (95% CI)
Adjusted OR (95% CI)
Height
High light scatter reticulocyte count
Mean sphered cell volume
Reticulocyte count
Eosinophill percentage
Haemoglobin concentration
LDL direct
HDL cholesterol
Cholesterol
Direct bilirubin
Aspartate aminotransferase
Apolipoprotein B
Apolipoprotein A
E78 Disorders of lipoprotein metabolism and other lipidaemias
E11 Non−insulin−dependent diabetes mellitus
E10 Insulin−dependent diabetes mellitus
J33 Nasal polyp
I26 Pulmonary embolism
I25 Chronic ischaemic heart disease
I10 Essential (primary) hypertension
D64 Other anaemias
Illnesses of siblings: Breast cancer
Illnesses of mother: Heart disease
Eosinophill count

Supplementary Fig. 15: Comparison of patients clustering cased on EpiXcan or PriLer gene expression models in liver.

a. Uniform manifold approximation and projection (UMAP) first 2 components of gene T-scores from EpiXcan models standardized across CAD patients, corrected for PCs, and multiplied by Z-statistic CAD associations. Each dot represents a patient in the transformed UMAP space colored by the cluster membership.

b. Contingency table for CAD patients of clusters based on EpiXcan imputation (rows) and PriLer imputations (columns).

c. Venn-diagram of unique cluster-specific endophenotypes ($FDR \leq 0.1$) from EpiXcan based and PriLer based clustering.

d. Among the endophenotypes measured in UKBB with at least one CAD associated and group-specific pathway, forest plot shows significantly different ones ($FDR \leq 0.1$) in at least one EpiXcan based group (gr_i versus remaining samples) using Generalized Linear Model (GLM), indicating regression coefficient (β_{GLM}) with 95% Confidence Interval (CI). Full dot indicates that β_{GLM} is significant after BH correction. Endophenotypes that are also significant in the PriLer based clustering are shown in bold (y-axis).

e. Pearson correlation of β_{GLM} from cluster-specific endophenotypes analysis for EpiXcan based groups (rows) and PriLer based groups (column). Squares with X indicate that the correlation is not significant at the nominal level 0.05.

f. Considering only pathway-scores in liver, venn-diagram of unique cluster-specific pathways ($FDR \leq 0.01$) from EpiXcan based and PriLer based clustering.

g. Pearson correlation of WMW estimates from cluster-specific pathways for EpiXcan based groups (rows) and PriLer based groups (column).

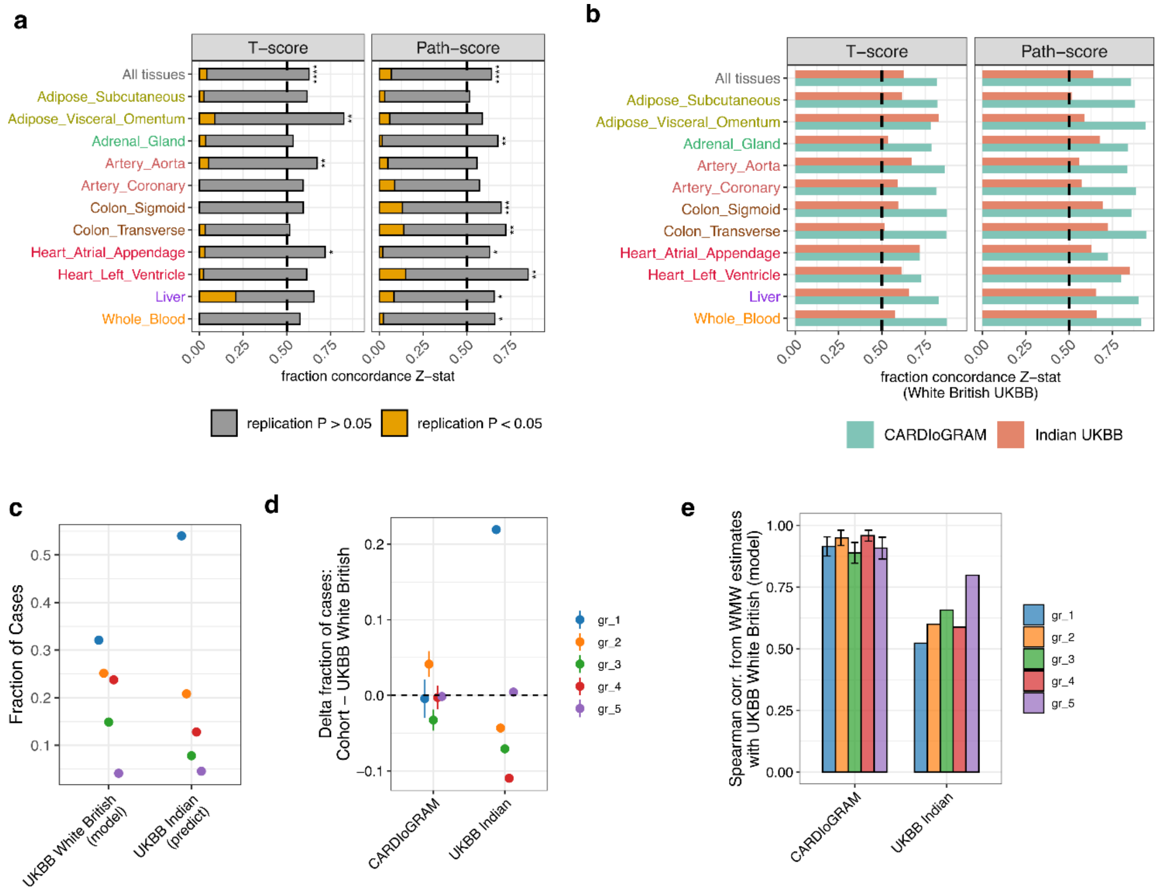

Supplementary Fig. 16: Trans-ancestry application of CASTom-iGEx.

CASTom-iGEx pipeline is applied in the context of CAD across Indian individuals in UKBB data set.

a. Reproducibility of TWAS (left) and PALAS (right) significant results for UKBB white British (UKBB WB) cohort in the UKBB Indian population. X-axis shows the fraction of significant genes in UKBB WB that have the same effect sign (Z-statistic) in UKBB Indian. P-values are computed from one-sided sign test ($* = P \leq 0.05$, $** = P \leq 0.01$, $*** = P \leq 0.001$, $**** = P \leq 0.0001$). The fraction of genes concordant and nominal at a p-value threshold of 0.05 is shown in the yellow bar.

b. Fraction of reproduced significant results based on Z-statistic sign for UKBB WB in Indian UKBB cohort (red) and CARDIoGRAM meta-analysis (green).

c. Projection of CAD cases clustering in liver from UKBB WB on UKBB Indian. Y-axis shows the fraction of cases assigned to each cluster in UKBB WB dataset and UKBB Indian.

d. Comparison with CARDIoGRAM clustering projection. Y-axis shows the difference in fraction of cases between the external cohort into which the clustering was projected (CARDIoGRAM or UKBB Indian) and the model clustering cohort (UKBB WB). For CARDIoGRAM, average across 9 cohorts +/- standard deviation (error bars) is displayed. **e.** For each group, Spearman correlation of WMW estimates in UKBB WB and the external cohorts (CARDIoGRAM and UKBB Indian) only from genes that are significantly associated with that group across all tissues. For CARDIoGRAM, average across 9 cohorts +/- standard deviation (error bars) is displayed.

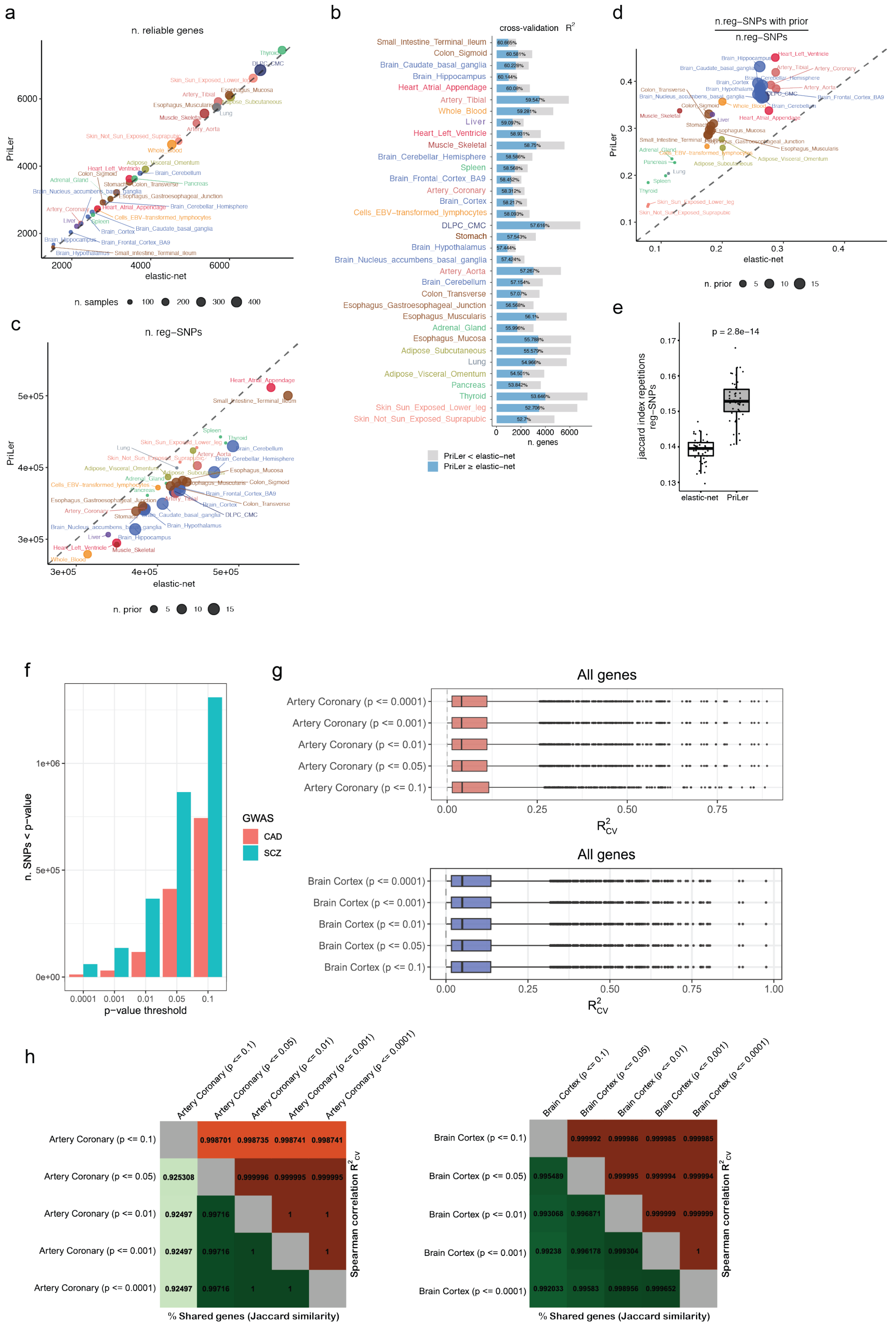

a
n. reliable genes
PriLer
elastic-net
n. samples
b
cross-validation R²
n. genes
PriLer < elastic-net
PriLer ≥ elastic-net
c
n. reg-SNPs
n. prior
d
n.reg-SNPs with prior
n.reg-SNPs
e
p = 2.8e-14
jaccard index repetitions reg-SNPs
f
n. SNPs < p-value
p-value threshold
GWAS
CAD
SCZ
g
All genes
h
Spearman correlation R^2_{CV}
% Shared genes (Jaccard similarity)

Supplementary Fig. 24. Comparison PriLer and elastic-net regression imputation models and benchmarking of GWAS threshold parameter in PriLer prior

For each tissue in GTEx/CMC panels comparison of PriLer and elastic-net regression without prior regarding

a. number of reliable genes i.e. $R^2_{cv} > 0$ and $R^2 > 0.01$,

b. number and percentage of genes among the reliable ones in PriLer models having better prediction performance (R^2_{cv}) in PriLer compared to elastic-net,

c. number of regulatory variants i.e. variant that regulates at least 1 gene,

d. fraction of regulatory variants that intersect with at least 1 prior feature used in PriLer tissue models,

e. Regulatory variants robustness in whole blood measured via bootstrapping 100 individuals 10 times and computing Jaccard index in each pair of repetition for PriLer and elastic-net models (p-value from Wilcoxon-Mann-Whitney test). Boxplot elements include median as central line, 1st and 3rd quartiles as box limits, 1.5 interquartile ranges from 1st and 3rd quartiles as corresponding whiskers.

f. Number of GWAS hits for CAD and SCZ across different p-value thresholds in the SNP variant set used in PriLer. The number of variants passing a certain threshold is comparable in CAD for p-value = 0.05 and in SCZ for p-value = 0.01.

g. In artery coronary (top) and brain cortex (bottom), distribution of PriLer model estimates in terms of average R^2 on test folds (R^2_{CV}) considering all reliable genes. Y-axis indicates the p-value threshold for GWAS to define the binary GWAS prior information used to train PriLer, respectively from CAD for artery coronary and from SCZ for brain cortex.

h. Heatmap with lower triangular part (green) indicating the percentage of shared reliable genes computed via Jaccard similarity and the upper triangular part (orange) indicating the Spearman correlation of R^2_{CV} for the genes in common. These pairwise measures are computed across GWAS thresholds for PriLer models in artery coronary (left) and brain cortex (right).

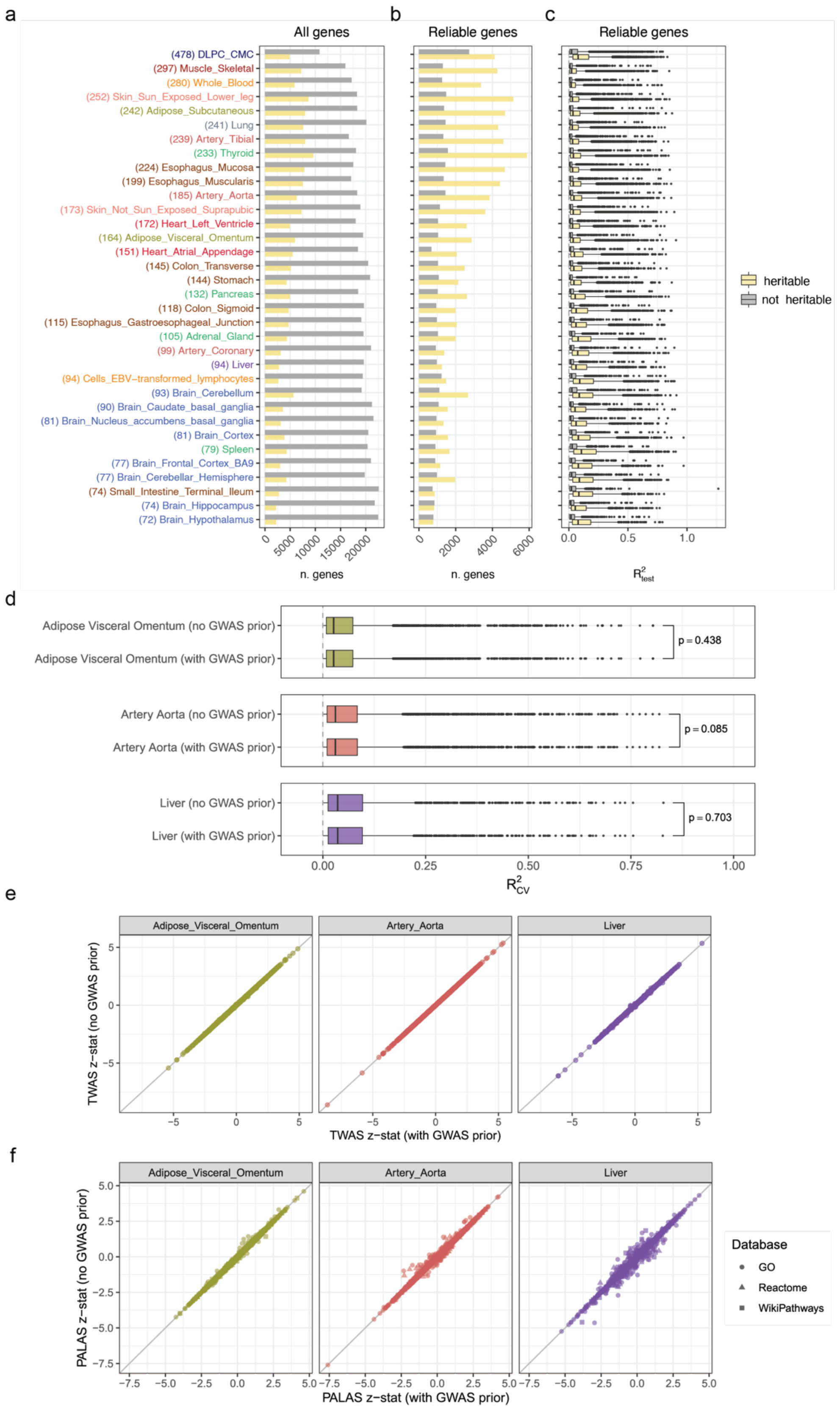
a
b
c
All genes
Reliable genes
Reliable genes
(478) DLPC_CMC
(297) Muscle_Skeletal
(280) Whole_Blood
(252) Skin_Sun_Exposed_Lower_leg
(242) Adipose_Subcutaneous
(241) Lung
(239) Artery_Tibial
(233) Thyroid
(224) Esophagus_Mucosa
(199) Esophagus_Muscularis
(185) Artery_Aorta
(173) Skin_Not_Sun_Exposed_Suprapubic
(172) Heart_Left_Ventricle
(164) Adipose_Visceral_Omentum
(151) Heart_Atrial_Appendage
(145) Colon_Transverse
(144) Stomach
(132) Pancreas
(118) Colon_Sigmoid
(115) Esophagus_Gastroesophageal_Junction
(105) Adrenal_Gland
(99) Artery_Coronary
(94) Liver
(94) Cells_EBV−transformed_lymphocytes
(93) Brain_Cerebellum
(90) Brain_Caudate_basal_ganglia
(81) Brain_Nucleus_accumbens_basal_ganglia
(81) Brain_Cortex
(79) Spleen
(77) Brain_Frontal_Cortex_BA9
(77) Brain_Cerebellar_Hemisphere
(74) Small_Intestine_Terminal_Ileum
(74) Brain_Hippocampus
(72) Brain_Hypothalamus
n. genes
n. genes
R^2_{test}
heritable
not heritable
d
Adipose Visceral Omentum (no GWAS prior)
Adipose Visceral Omentum (with GWAS prior)
p = 0.438
Artery Aorta (no GWAS prior)
Artery Aorta (with GWAS prior)
p = 0.085
Liver (no GWAS prior)
Liver (with GWAS prior)
p = 0.703
R^2_{CV}
e
Adipose_Visceral_Omentum
Artery_Aorta
Liver
TWAS z−stat (no GWAS prior)
TWAS z−stat (with GWAS prior)
f
Adipose_Visceral_Omentum
Artery_Aorta
Liver
PALAS z−stat (no GWAS prior)
PALAS z−stat (with GWAS prior)
Database
GO
Reactome
WikiPathways

Supplementary Fig. 25. Gene expression variability for heritable and not heritable genes explained by PriLer and comparison of TWAS and PALAS from PriLer models trained without GWAS prior.

a. Number of total genes across the heritable and not heritable categories.

b. Number of reliable genes across the heritable and not heritable categories.

c. Average R^2 on test folds for reliable genes across all tissues, divided by heritable (yellow) and not heritable (grey) genes. The tissues are ordered on the y-axis according to their sample size. Boxplot elements include median as central line, 1st and 3rd quartiles as box limits, 1.5 interquartile ranges from 1st and 3rd quartiles as corresponding whiskers.

d. In three CAD related tissues (adipose visceral omentum, artery aorta, liver), distribution of PriLer model estimates in terms of average R^2 on test folds (R^2_{CV}) considering all reliable genes, with PriLer trained with CAD GWAS prior or without. P-value on top is from paired t-test.

e. TWAS on CARDIoGRAM cohorts based on PriLer gene expression models with CAD GWAS prior (x-axis) or without (y-axis).

f. PALAS on CARDIoGRAM cohorts based on PriLer gene expression models with CAD GWAS prior (x-axis) or without (y-axis), dot shape refers to the pathway database.

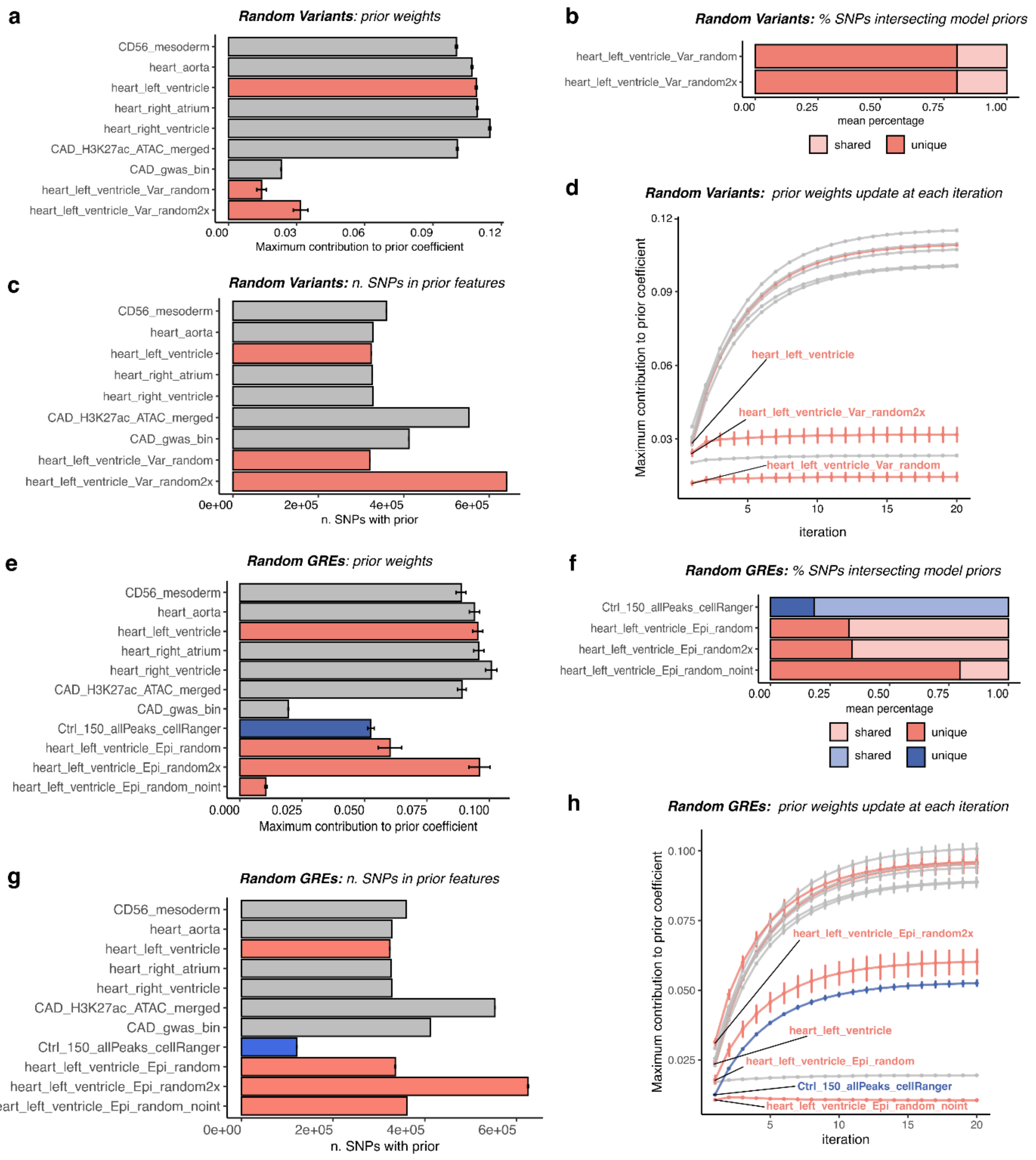

Supplementary Fig. 26. Simulation of prior features to test weights their relevance in Artery Coronary tissue.

In the first scenario (*Random Variants*), 2 prior features were randomly generated 50 times *heart_left_ventricle_Var_random* and *heart_left_ventricle_Var_random2x* using the same size/

twice the number of prior features as in *heart_left_ventricle* (pink). In the second scenario (*Random GREs*), open chromatin regions were randomly selected (i.e. gene regulatory elements GREs) from ChIP-Seq H3k27ac data 50 times using the same or twice feature set size as *heart_left_ventricle* and intersected the latter with variants location to create *heart_left_ventricle_Epi_random* and *heart_left_ventricle_Epi_random2x priors* (pink). This included also a prior feature related to brain tissue *Ctrl_150_allPeaks_cellRanger* (blue) as well as random prior *heart_left_ventricle_Epi_random_noint* sampling among GREs excluding the ones in the baseline prior features and in the same size as GREs for *heart_left_ventricle*. In both scenarios, we created PriLer models including other 6 fixed prior features for Artery Coronary.

a, **e.** mean ± SD prior weights for each prior feature,

b,. **f.** mean percentage of variants in the random priors features that are in common with fixed features used in the model,

c., **g.** mean ± SD number of variants associated to each prior features,

d., **h.** mean ± SD update of prior weights at each iterative step.

Tissue	N of samples	N of genes	N of prior features	N of reliable genes	N of reg-SNPs	% reg-SNPs with Prior
Dorsolateral Prefrontal Cortex	478	15578	15	6854	366706	36.65
Adipose Subcutaneous	242	25971	3	6058	423635	25.85
Adipose Visceral Omentum	164	25139	3	3910	386744	27.68
Adrenal Gland	105	23624	1	3027	377947	23.5
Artery Aorta	185	24274	7	5277	402688	38.45
Artery Coronary	99	23880	7	2298	343528	39.24
Artery Tibial	239	24335	7	5918	364109	41.93
Brain Caudate basal ganglia	90	24512	15	2635	349538	39.59
Brain Cerebellar Hemisphere	77	23762	15	2941	393519	39.13
Brain Cerebellum	93	24570	15	3788	429782	36.47
Brain Cortex	81	24110	15	2501	368497	38.62
Brain Frontal Cortex BA9	77	23765	15	2041	372502	38.05
Brain Hippocampus	74	23723	15	1671	313967	43.17
Brain Hypothalamus	72	24426	15	1565	342292	37.26
Brain Nucleus accumbens basal ganglia	81	24386	15	2290	341053	36.89
Cells EBV-transformed lymphocytes	94	21779	2	2706	372078	26.15
Colon Sigmoid	118	24051	8	2925	379960	33.05
Colon Transverse	145	25354	8	3522	374357	30.48
Esophagus Gastroesophageal Junction	115	23575	8	3030	346204	28.65
Esophagus Mucosa	224	25038	8	6107	381943	31
Esophagus Muscularis	199	24360	8	5754	379200	29.5
Heart Atrial Appendage	151	23666	7	2733	511496	33.77
Heart Left Ventricle	172	22681	7	3628	294768	45.07
Liver	94	22158	2	2215	306512	32.95
Lung	241	27372	1	5749	399559	20.4
Muscle Skeletal	297	22942	2	5566	292706	33.73
Pancreas	132	23153	1	3631	361244	22.7
Skin Not Sun Exposed Suprapubic	173	25922	1	4740	407576	13.36
Skin Sun Exposed Lower leg	252	26582	1	6614	427755	13.79
Small Intestine Terminal Ileum	74	25010	8	1594	500251	28.62
Spleen	79	24354	1	2556	442687	19.9
Stomach	144	24861	8	3215	339228	29.71
Thyroid	233	27305	1	7447	434307	18.44

Whole Blood	280	22805	6	4644	279175	35.69

Supplementary Table 1. Summary of tissue-specific gene expression models via PriLer. Summary of tissue specific gene-expression models in terms of number of samples, genes, prior feature applied, reliable genes, regulatory variants (reg-SNPs) and percentage of reg-SNPs intersecting a prior used in the model.

Supplementary Note 1

Validation and comparison of Priler against elastic-net regression

Since PriLer is an extension of elastic-net regression (enet) that incorporates prior knowledge on individual variants, we initially benchmarked PriLer against enet across 34 tissue-specific models (33 GTEx and 1 CMC). First, we compare PriLer and enet in terms of reliable genes i.e. genes predicted from genetic data having $R^2 \geq 0.01$ and $R^2_{cv} > 0$. The number of reliable genes is very similar (**Supplementary Fig. 24a**) but always higher for PriLer for a total of 2,922 additional genes (mean ± sd: 85.94 ± 47.39). In addition, for reliable genes in PriLer, we observed an increase in number of genes having higher R^2_{cv} in PriLer compared to enet (**Supplementary Fig. 24b**), showing an overall better prediction performance. The number of genes with improved prediction performance is partly correlated with number of priors used in the model across tissues (Pearson corr. 0.48) and negatively with the number of training samples (corr. -0.28). PriLer not only increases the number of genes that can be accurately predicted, but also decreases the number of reg-SNPs (**Supplementary Fig. 24c**), with a total decrease across all genes of 1,462,466 variants (mean ± s.d. 43,014 ±14,530). The difference in number of reg-SNPs significantly depends on the number of prior features (corr. -0.68). Moreover, we observe an increase in fraction of reg-SNPs that contain any prior information used in PriLer model (**Supplementary Fig. 24d**). The mean increase is 11% (sd 3.32%) with the difference in fraction of reg-SNPs with prior being partly

dependent on the number of prior included (corr. 0.26). In addition, we compared reg-SNPs robustness in whole blood tissue, downsampling to 100 individuals 10 times and comparing reg-SNPs selection in each pair of repetition using Jaccard index (**Supplementary Fig. 24e**), PriLer shows a significant increase in terms of concordance of selection with respect to enet (Wilcoxon-Mann-Whitney P=2.8e-14). In summary, PriLer generates better performing models of genotype-based expression imputation, using a reduced number of variants but more biologically meaningful and robust compared to elastic-net regression without prior information.

Finally, we observe the differences in terms of predictive performances for heritable and not heritable genes defined a priori via GCTA software in PriLer. The majority of expressed genes are not heritable across all tissues (**Supplementary Fig. 25a**). Thus, prior weights are calibrated on a smaller set of genes whose size varies with the training sample size. On the other hand, as expected heritable genes constitute most of the reliable genes defined by PriLer **(Supplementary Fig. 25b**), and the variance explained for heritable genes is always significantly higher compared to not heritable ones in the same tissue (**Supplementary Fig. 25c**, Wilcoxon-Mann-Whitney p-value < 2.21^{-49}). Overall, median prediction accuracy of heritable-vs-non heritable genes differs by 0.0398 on average, inversely dependent on overall training sample size (Spear. correlation = -0.8128) and ranging from 0.0125 for whole blood to 0.077 for spleen. Similarly, the proportion of heritable-vs-non-heritable genes depends on sample size (Spear. correlation = 0.8105), with proportions ranging from 49% for hippocampus to 78% for thyroid.

<u>GWAS threshold to define PriLer prior</u>

To define GWAS prior when training CAD related tissues and SCZ related tissues we choose p-value threshold of 0.05 for CAD and 0.01 for SCZ. This selection led to a similar number of

variants having that prior information (**Supplementary Fig. 24f**) so that the effect was comparable.

Regardless, the choice of the threshold did not affect PriLer model performances. We tested this in artery coronary with CAD GWAS threshold and in brain cortex with SCZ GWAS threshold and varied the p-value cut-off as 0.0001, 0.001, 0.01, 0.05, 0.1.

The distributions of average R^2 on test folds (R^2_{CV}) for reliable genes across threshold were similar (**Supplementary Fig. 24g).** The percentage of shared reliable genes was high: artery coronary > 92% and brain cortex > 99%, and for those in common the model performances were highly correlated (spearman corr. > 0.99 for both tissues) (**Supplementary Fig. 24h).** We concluded that model performances were invariant by the choice of threshold to define GWAS prior information.

<u>GWAS prior does not overfit CAD associations on CARDIoGRAM</u>

PriLer models were trained on CAD harmonized variant set with and without CAD GWAS as prior (binarized with threshold 0.05) across 3 tissues, adipose visceral, artery aorta and liver. Among the common reliable genes, the distribution of average R2 on test folds (R^2_{CV}) were similar (**Supplementary Fig. 25d**, paired t-test > 0.05). Indeed, we then compared the TWAS from imputed gene expression and PALAS from pathway-scores derived from PriLer models with and without CAD GWAS prior (**Supplementary Fig. 25e-f**). Z-statistics associations were almost identical for TWAS results and very similar for PALAS, with some subtle differences for weaker associations.

We observed no trend of increased associations when using prior GWAS, although it was also derived using CARDIoGRAM cohort. We concluded that the use of CAD GWAS prior led to no overfit in the observed associations.

Evaluation of prior weights selection in PriLer through random prior simulation

To examine whether the learned weights for prior features were meaningful for the model tissue considered, we simulated random prior features using as example artery coronary tissue and focusing on *heart_left_ventricle* prior features that indicate whether a variant is located in an open chromatin position based on H3K27ac for heart left ventricle cell type. We define as baseline prior 7 priors that are normally adopted in artery coronary model tissue (**Supplementary Data 13**).

First, we define two new prior features called heart_left_ventricle_Var_random and heart_left_ventricle_Var_random2x randomly selecting variants in the same size or twice respectively of the original prior feature heart_left_ventricle (**Supplementary Fig. 26c**). The aim is to emulate a prior that is not biologically meaningful but contain the same amount of information or twice of an existing one. The estimates for prior weights across 50 repetitions are close to zero although different from it (mean ± sd = 0.0145 ± 2.04e-03 and 0.0317 ± 3.22e-03) (**Supplementary Fig. 26a**) with Var_random2x increased compared to Var_random but still lower than the original prior heart_left_ventricle (mean ± sd = 0.109 ± 4.01e-04). Indeed, when a prior feature intersects SNPs that are used even to a small extent in a gene regression model, the initial estimate cannot be exactly zero and the bigger the prior size (number of variants it intersects), the more likely is that prior to be relevant just because of randomly intersecting reg-SNPs. In addition, just by chance, the variants randomly selected still intersects baseline prior features that are used in the model (mean sharing 20%, **Supplementary Fig. 26b**). However, in the iterative procedure, the weights for the randomly created priors remain fixed instead of increasing until convergence as it happens for the original prior (**Supplementary Fig. 26d**). This means that the use of variants

intersecting heart_left_ventricle in the gene regression models increases the performance, which does not happen for the randomly generated priors.

Second, we generated random prior features that resemble ChIP-Seq H3k27ac data used to build prior information. To this end, we randomly select open chromatin regions i.e. gene regulatory elements (GREs) from the original data in the same size or twice as *heart_left_ventricle* and intersected with variants location to create *heart_left_ventricle_Epi_random* and *heart_left_ventricle_Epi_random2x* priors. In addition, we included *Ctrl_150_allPeaks* which is a prior feature related to brain tissue. Differently from the first scenario that just extrapolates variants by chance, sampling GREs allows taking into consideration genomic positions and LD structure. The randomly selected GREs across 50 repetitions partly overlap with baseline GREs used in artery coronary tissue (**Supplementary Fig. 26f**) resulting in a sharing of 67% and 65% of variants in *Epi_random* and *Epi_random2x* respectively as well as 81% shared variants with *Ctrl_150_allPeaks*. Thus, to generate a random prior that would not show a high sharing with the baseline model, we randomly selected GREs excluding the ones used in the baseline prior features and in the same size as GREs for *heart_left_ventricle*. The newly created prior feature (*Epi_random_noint*) only shares 20% of the variants detected among the baseline priors due to GREs possible overlapping. The number of variants from randomly generated priors are similar to the original *heart_left_ventricle* for *Epi_random* and *Epi_random_noint* while twice the amount for *Epi_random_2x* (**Supplementary Fig. 26g**). Differently from the first scenario, the estimate for prior weights *Epi_random Epi_random_2x* and *Ctrl_150_allPeaks* are very different from zero (mean ± sd = 0.06 ± 0.005, 0.096 ± 0.004, 0.052 ± 0.001). The only random prior reaching the similar weight as *heart_left_ventricle* (0.095 ± 0.002) is *Epi_random_2x*, which includes twice the amount of the information than the original (**Supplementary Fig. 26e**), while *Epi_random_noint*

estimates are very close to zero (0.01 ± 0.0004). Although the new prior included in the model are not related to artery coronary, the relevance can be explained by the high sharing in terms of variants with respect to the baseline model. Indeed, when the percentage is reduced as in the case of *Epi_random_noint,* the associated weight is close to zero. Regardless *Epi_random_2x* starting at higher relevance due to the increased size, it just reaches the same value of original *heart_left_ventricle* at convergence (**Supplementary Fig. 26h**).

We conclude that the weights reflect a tissue specific configuration of gene expression regulation that can be partially confounded by high sharing of variants with actual relevant prior features. However, not relevant prior weights are reduced to the minimum when their sharing with relevant priors is only marginal, even in case of existing GREs reflecting genome structure.

<u>Clustering simulation in CAD</u>

To generate an empirical null distribution of gene, pathway, and endophenotype associations with clustering structure, we randomly partitioned UKBB CAD patients 50 times into similar-sized groups compared to actual liver-based clustering. All random partitions but four were independent of the actual clustering (**Supplementary Fig. 10a**), with repetitions 1, 4, 29 and 31 only showing a mild association ($P > 0.01$) and not passing FDR 0.05 threshold ($FDR > 0.5$). Considering the first 10 repetitions due to computational time constraints, we then detected the group-specific genes and pathways across clusters. The WMW p-value distributions mostly did not deviate from the expected uniform distribution (**Supplementary Fig. 10d, Supplementary Fig. 11f**) with some exceptions for genes such as repetition 9 in group 4. Observing the number of associations passing FDR thresholds, across each group the 0.01 upper bound identifies at max two genes significant in 1 out of 10 repetitions (**Supplementary Fig. 10e**), at max two pathways significant in 1

repetition, and maximum 4 repetitions with at least one pathway significant (gr3) (**Supplementary Fig. 11g**). Thus, to reduce the number of false-positives, we used as FDR threshold for cluster-specific genes and pathways of 0.01 instead of the otherwise used 0.05. Finally, we computed the endophenotype differences in each cluster via GLM across the 50 random clustering testing 635 UKBB phenotypes. We compared the effect size β_{GLM} and the corresponding -log10 p-value from random clustering repetitions and liver cluster (**Supplementary Fig. 11h**). Extremely significant results were only achieved for liver clusters and maximum 2 endophenotypes were significant (FDR 0.05) in 9 different repetitions. In conclusion, FDR cut-off of 0.01 ensures a reduction of false positives for cluster-specific genes and pathways. Moreover, the empirical null-distribution of endophenotype associations leads to almost no significant results and is different compared to the clustering in liver. Endophenotype associations in random clusters were also tested for the 33 hypothesis-driven clinical phenotypes and used to compute the empirical p-value (see "Detection of endophenotype differences across patient strata" in Methods).

Calibration of type 1 error in TWAS and PALAS

In order to determine whether the approach to TWAS and PALAS proposed here provided well-calibrated p-values both at the gene and pathway-score levels, we considered whole blood as exemplar tissue that included 3,840 genes, 902 Reactome pathways and 2,803 GO pathways and simulated random phenotypes 50 times. In detail, we created binary vectors that resembled CAD phenotype keeping the same case/control size, i.e. 19,026 cases and 321,913 controls. To create random phenotypes that resembled as closely as possible the same confounders as the actual CAD classification, we selected the same number of female/males and the same age compared to the actual CAD phenotype among the case/control classes. We then performed TWAS and PALAS

and tested for associations between the randomly created phenotypes and gene T-scores and pathway-scores that were previously computed for CAD (i.e. considering as reference set a subset of individuals non-affected by CAD). Finally, multiple-testing correction is performed via BH procedure, correcting for each simulation separately. Combing all the simulations, we observed that p-value distribution approximates a uniform distribution in (0,1) range (**Supplementary Fig. 10b**, **Supplementary Fig. 11a-b)**, validated also via Kolmogorov-Smirnoff test that compared a random uniform distribution with the simulated one form gene associations (p-value=0.17), pathway associations in Reactome (p-value=0.87) and pathway associations in GO (p-value=0.5). The same conclusions can be drawn from quantile-quantile plots in **Supplementary Fig 10c-e, Supplementary Fig. 11c-h,** with the expected distribution of p-value extracted from a uniform one. The association signal with the actual CAD phenotype greatly diverged from the simulated ones, with very few genes/pathways passing the FDR 0.05 threshold in the simulated phenotypes (blue points). However, all simulation results remain in the 95% confidence intervals of the standard uniform order statistics that follows a beta distribution. We can then conclude that CASTom-iGEx strategy for TWAS and PALAS returns well-calibrated p-values.

Relevance of genes correlation in pathway significance

Next, we investigated whether gene correlation and LD structure were connected to the increase observed in pathway-score significance. To this aim, we performed two analyses:

1. Simulation of pathway structure from actual gene T-scores in whole blood, creating gene-sets composed of 3 or more genes located in the same loci, for a total of 46 simulated pathways. In this case, the goal was to understand how the loci structure can influence the pathway significance.

2. Estimation of relationship between pathway significance increase and average gene correlation across all detected pathways with $n \geq 2$, to observe the actual relevance and extent of genes correlation in pathway significance.

For 1., we only considered actual genes in whole blood that were showing a certain level of significance i.e. nominal TWAS p-value 0.01 and created simulated gene-sets from those genes that were also in the same loci and had the same effect size sign in CAD associations (all Z-stat genes >0 or < 0), to avoid a compensatory effect for gene relevance. This procedure led to a total of 46 simulated pathways with the number of genes included varying from 3 to 7. Although all the genes were in the same loci, the increase in pathway significance was dependent and inversely proportional to the estimated average genes correlation (**Supplementary Fig. 12e**), with almost no increase for pathways that included highly correlated genes. This resulted in a general lower significance of pathways composed of correlated genes (**Supplementary Fig. 12f**). Based on these findings we concluded that the gene correlation due to the regulation from the same variants (or in LD with them) rather than the vicinity of genomic coordinates is relevant in the observed pathway significance and that genes in the same loci not correlated do still lead to an improvement in the information captured by the pathway scores.

For 2., we finally considered the actual pathway-scores and increase or decrease in pathway association level compared to the average genes correlation included in the pathways. Across all the pathways databases, there was no rank correlation between average differences of significance in pathways versus genes and genes correlation (**Supplementary Fig. 12g-i**, absolute Spearman corr. < 0.045). Indeed, we observed that pathways with highly correlated genes (> |0.5|), usually including less than 4, showed only marginal improvement in pathway significance. In contrast, pathways with a striking effect of increased significance were those formed by more than 10 genes

and having and average correlation around zero. Hence, we conclude that the increase in pathway relevance with respect to single genes became maximal when the correlation among genes was minimal. Overall, genes correlation due to LD structure did not increase pathway significance nor pathway improvement compared to single genes. Finally, observing actual pathway structures, the gene-sets with best improvement were formed by not correlated genes.

Gene T-scores reduce samples correlation and leads to the same distribution for each gene

We computed gene T-scores instead of using imputed gene expression to obtain a similar distribution for all the genes. To show this, we considered the 9 CARDIoGRAM cohorts across the 11 CAD tissues. First, we computed the pairwise correlation among samples in each tissue and cohort. The gene T-score that we proposed did not introduce any spurious correlation among samples but drastically reduced it. Indeed, the median of the pairwise correlation among samples and in each tissue is > 0.6 for the imputed gene expression and drastically drops at around zero after computing gene T-scores (**Supplementary Fig. 4a**). In addition, in each cohort and across all tissues, we computed the mean and variance of genes across samples, before and after T-score computation. The mean of each gene now is around zero (0.01, and 0.99 quantiles are -1.30 and 1.06 versus -0.06 and 0.06, Fig. **Supplementary Fig. 4c**). The variance got shifted from a distribution mostly between 0-1 (mean = 0.08, var = 0.01) to a distribution around 1 (mean = 1, var = 0.001, **Supplementary Fig. 4d**). Importantly, the original gene variance is highly correlated with PriLer predictive performances (as expected). The overall correlation is 0.95 and after T-score computation it reduced to almost no corr. at 0.08 (**Supplementary Fig. 4b**). We can conclude that this transformation does not induce correlation but removes the ones present in the data (from genetic info). In addition, it rescales genes such that they have mean 0 and variance 1 and they are

not dependent on the PriLer predictive performance anymore. In this way, the variability that goes into the pathway-score computation is always the same and not dependent on the predictive performance of the genes.

Selection of K- Nearest Neighbor parameter and correlation threshold for clumping in clustering

To select the optimal kNN parameter and gene correlation threshold for clustering, we investigated the variability of the resulting structure in CAD UKBB. In the first case, for each tissue, we kept the clumping threshold correlation fixed at 0.9 and clustered samples with different kNN parameters (5, 10, 20, …, 100). For each parameter, we computed the coverage and conductance of the cluster via clustAnalytics R package (https://CRAN.R-project.org/package=clustAnalytics). A high coverage ensures intra-cluster connections, whereas a low conductance indicates that there are few edges connecting different groups, thus we considered *coverage+1–conductance* the metric to be maximized. Across the kNN values, the results were stable with the only exception of kNN = 5 and the median best result was obtained for kNN = 20 (**Supplementary Fig. 5b**). Fixing kNN to 20, we then bootstrapped 50% of the samples 10 times and perform again the clustering step. The NMI between the original clustering and the repetitions of bootstrapped samples varies depending on the tissue, with liver and adipose subcutaneous showing the most stable results (**Supplementary Fig. 5c**). Interestingly, the robustness is dependent on the clustering quality of the tissue assessed via *coverage+1–conductance* (**Supplementary Fig. 5d**), with liver showing the best performances in terms of both NMI and cluster quality. We concluded that kNN = 20 was a suitable choice that ensured the best clustering structure across tissues and robustness, especially for well-separated tissues (adipose subcutaneous and liver NMI > 0.6).

We then studied the differences in clustering results varying the correlation threshold (corr. thr) parameter to filter genes via clumping and kept kNN = 20. Across (0.1, 0.2, …, 1) possible values, the clustering quality given by *coverage+1–conductance* across tissues was higher for corr. thr≥ 0.8 (**Supplementary Fig. 5e**). For the same values, the final number of clusters were less variable among tissues and with a median = 5 (**Supplementary Fig. 5f)** Among values 0.8, 0.9 and 1, corr. thr = 0.9 showed the highest number of cluster-associated loci in that tissue (BH corrected p-value ≤ 0.05 from Kruskal-Wallis test) (**Supplementary Fig. 5g**). This indicates that 0.9 threshold leads to more heterogeneous results in terms of genes involved in defining the clusters while creating the best-quality clusters (**Supplementary Fig. 5h**).

Benchmark of genes TWAS-scaling in clustering

The third step in the genes pre-processing for community detection is the multiplication of each gene by Z-statistic of the phenotype of interest in the considered tissue from TWAS. We compared this strategy (called z-scaled) with the complementary of no TWAS-scaling (called original) in CAD UKBB. The number of detected groups was always higher in the original configuration and more than 15 in each tissue (**Supplementary Fig. 6a,** left). More importantly, the cluster quality measured via coverage (intra-cluster connections) + 1 – conductance (inter-cluster connection) was always higher in the z-scaled version (**Supplementary Fig. 6a,** right), revealing a better-defined structure after z-scaling. In addition, the concordance of the cluster results between the two strategies measure via NMI was minimal and variable across tissues (**Supplementary Fig. 6b**). In liver, we additionally identified cluster-specific genes and pathways for both strategies and compared the WMW estimated of the significant ones (FDR ≤ 0.01) with the CAD TWAS Z-statistic (**Supplementary Fig. 6c-d**). Only in the z-scaled version, we observed a relationship

between the two (Pear. Corr. = 0.53), that in turn was almost zero in the original version (|Pear. Corr.| < 0.05). This indicates that the z-scaling strategy allows 1) to achieve more well-separated and connected clusters, 2) for genes/pathways that are more relevant for CAD to have a higher impact and consequentially be more associated with the final clustering configuration.

Investigation of ancestry contribution to clustering structure

To reduce possible biases in the clustering structure given by ancestry, we correct imputed gene expression for 10 genotype-based PCs as pre-processing step prior clustering. In the context of CAD, we observed that clustering of the data on the residuals compared to no correction showed no/minimal remaining associations with PCs (**Supplementary Fig. 5i**). This step only minimally changed the clustering structure compared to no correction, indicating that the overall impact of population structure as captured by PCs was already small (NMI > 0.47, **Supplementary Fig. 5j**). In the context of CAD clustering in liver and SCZ clustering in DLPC, PCs association reduced after correction and did not pass FDR 0.05 for CAD (**Supplementary Fig. 7c**) but was still significantly associated with the detected clusters in SCZ (**Supplementary Fig. 17b)**. Nevertheless, in both CAD and SCZ we observed that the actual PC distribution across clusters (**Supplementary Fig. 7c, Supplementary Fig. 13b**) was not driving the partitioning compared to the imputed gene expression based on effect size estimates (**Supplementary Fig. 7d, Supplementary Fig. 17d**) hence not separating the patient space. In fact, the coefficients of variations (effect-sizes divided by confidence interval range) specific to each group for the strongest associated PCs (PC4-PC5 in CAD and PC1-PC5 in SCZ) were below 0.79 and 2.48 in absolute value compared to the top 5 genes per group (P < 1e-46) that showed a coefficient of variation > 4 and 8 respectively **(Supplementary Fig. 7d, Supplementary Fig. 17d**). For SCZ the

minimum coefficient of variation is 8 when excluding gr4, which due to the small sample size has a low coefficient of variation. Nevertheless, there are some top gr4 genes that surpass the top coeff. of variation from PCs such as HLA-DRB1 reaching 4.15. In general, coeff. of variation reaches peaks such as 306 for SORT1 and 85 for C4A, respectively for CAD and SCZ.

To better characterize the PCs contribution, we performed clustering of individuals based solely on PCs and repeated the endophenotype analysis (see "Clustering based on genotype derived principal components" in Methods, **Supplementary Fig. 8, Supplementary Fig. 18**). Both for CAD and SCZ, we found marginal overlap between tissue based clusters and PCs based ones (NMI < 0.005, **Supplementary Fig. 8b, Supplementary Fig. 18b),** although significant based on chi-squared test for liver in CAD and in all tissues for SCZ. Specifically in liver for CAD and DLPC for SCZ, the minimal overlap was not null and greater than what is reached by a randomly assigned clustering structure (**Supplementary Fig. 8c, Supplementary Fig. 18c**). To understand which groups from tissue-based and PCs based shared a higher by chance number of individuals, we compared pairwise Fisher's Exact test odds ratio (**Supplementary Fig. 8d, Supplementary Fig. 18d**). For CAD, an enrichment was detected between gr1 in PCs and gr2 in liver, with a consequential depletion between gr1 in PCs and gr1 in liver. This could be related to a higher fraction of samples in gr1 PCs and gr2 liver originally from Reading and Birmingham surroundings (**Supplementary Fig. 8e**). For SCZ instead, 10 pairs showed either a significant enrichment or depletion (p-value < 0.01), with strongest enrichment among gr3 in DLPC and gr6 in PCs (**Supplementary Fig. 18d**).

Most importantly, we observed that the minimal overlap found between tissue-derived and ancestry-derived clustering did not influence the group-specific endophenotype differences (**Supplementary Fig. 8f-g**, **Supplementary Fig. 18e-f**). For CAD, we observed different

endophenotype significance among the two clustering structures (**Supplementary Fig. 8f**), with place of birth in UK being the strongest signal in PCs clustering not significant for liver clustering, as expected (**Supplementary Fig. 8f**). For the eight endophenotypes significant in both clustering configurations, we additionally examined whether this was related to the mild overlap between gr2 in liver and gr1 in PCs. Looking at cluster-specific effect sizes (**Supplementary Fig. 8g**), hyperlipidaemia diagnosis was lower in both gr5 liver and gr3 PCs and it is the only result in concomitance of enrichment between the strongest groups. Indeed, every other endophenotype is not in correspondence of group depletion or enrichment apart from height (enrichment gr1 liver and gr2 PCs), but the effect is opposite and hence this enrichment did not influence the endophenotype differences. Similarly, for SCZ the trend among best p-value endophenotype association was different between DLPC and PCs cluster (**Supplementary Fig. 18e)** and with a great variability in magnitude. However, 3 endophenotypes passing FDR 0.05 threshold were identified in both partitions. Considering results with FDR 0.1 threshold, we then investigated the group-specific differences for the strongest association in each endophenotype (**Supplementary Fig. 18f**). Platelet crit is lowest in two depleted groups (DLPC gr2 and PCs gr5), hence the direction is discordant with the cluster overlap. Differences in Mean MD in fornix cres+stria terminalis on FA skeleton (left) and Mean L1 in fornix cres+stria terminalis on FA skeleton (left) are observed in two groups that are not enriched (DLPC gr2 and PCs gr3). The only endophenotype with concordant result based on group overlap was Volume of grey matter in Inferior Frontal Gyrus, for which we decreases in gr4 PCs and in gr1 DLPC which were indeed enriched for shared individuals. Jointly, these results show that patient groups and detected endophenotype differences in our analysis were not driven by PCs as would be expected if population structure had a major impact on overall clustering.

Clustering of non-affected individuals

We tested the CASTom-iGEx clustering on a set of non-affected individuals and compared the results to CAD clustering in Liver. In particular, we extracted random individuals as those not having CAD SOFT annotation, nor family history (mother, father or siblings) of heart disease and stroke. Among these UKBB samples, we matched the age / sex distribution that we have in CAD cases and obtained a total of 19,023 non-affected individuals with 14,690 males and 4,332 females. We performed this analysis with 2 different pre-processing: a) weighting their re-scaled gene T-scores by the CAD TWAS effect of each gene (**Supplementary Fig. 13a-e**), b) not using any CAD related weights (**Supplementary Fig. 13f-i**). In the first setting, the number of groups is the same, with a similar fraction of cases distribution and cluster quality (coverage + 1 – conductance: 1.402 cases and 1.394 controls) (**Supplementary Fig. 13a**). Indeed, when looking at the association of genes with clusters, there are some groups in cases that are more correlated with others in controls (**Supplementary Fig. 13b**). This trend is also true for pathways but to a smaller extent (**Supplementary Fig. 13c**). Based on the strongest correlations, we can assume that gr1 cases corresponds to gr1 controls, gr2 cases corresponds to gr4 controls, gr3 cases corresponds to gr2 controls, gr4 cases corresponds to gr3 controls, and gr5 cases corresponds to gr5 controls (corr. > 0.7). Nevertheless, when considering endophenotypes associations, the overall trend pairing of cases and control groups is not observed anymore (**Supplementary Fig. 13d**) and the identified control groups show a very weak cluster specific disease relevant endophenotype association when compared to patient derived clusters (**Supplementary Fig. 13e)**. On the other hand, clustering random individuals without re-weighting by the CAD TWAS effects yields clusters largely

unrelated to the patient clusters in structure (**Supplementary Fig. 13f**) with very limited overlapping group specific genes, pathways or endophenotypes (**Supplementary Fig. 13g-i**).

We conclude that the clustering is similar in terms of drivers when applying the TWAS-scaling strategy, but the consequences at the level of endophenotypes are different. Hence the clustering phenotypic arise only in the context of the disease. On the other hand, without any CAD information, both endophenotypic and molecular characterization of control group greatly diverge from the cases grouping.

Validation of gene risk scores to mimic actual phenotype in cluster-specific differences

To evaluate the reliability of cluster-specific differences for endo-RS in term of actual differences in corresponding endophenotype, we defined a cluster-reliable measure (CRM). We calibrated a reasonable threshold for CRM based on CAD analysis and UKBB phenotyping. This threshold is subsequently applied to SCZ cluster-specific endo-RS differences to highlight significant results that are likely to be observed also in the actual phenotype.

First, we build endo-RS weights (Z-statistics) for 369 CAD related phenotypes in UKBB in 10 GTEx tissues. Then, we compute endo-RS separately on each CARDIoGRAM cohort and tissue, correcting each gene for the cohort-specific first 10 PCs and considered the projected clustering structure based on UKBB CAD tissue-specific results. Via meta-analysis similar to TWAS and PALAS, endo-RS group-specific differences across all cohorts are summarized and CRM for each group-endophenotype combination is computed as described in Methods ("Risk scores computation and differences detection in cases stratification") for those passing FDR 0.05 cluster-specific significance. Success rate of endo-RS reliability, i.e. actual endophenotype differences detected for the same clustering structure in UKBB, is measure in term of precision

(**Supplementary Fig. 20**). This is computed based on the fraction of group-specific endo-RS differences having same sign of β_{GLM} in endo-RS and actual endophenotype analysis in UKBB among all the endophenotypes passing a certain CRM threshold:

$$Precision = \frac{\#(\beta_{GLM}^{gene-RS} \cdot \beta_{GLM}^{pheno} > 0 \quad \wedge \quad CRM_{gene-RS} > thr_{CRM})}{\#(CRM_{gene-RS} > thr_{CRM})}$$

Combining all tissues together, CRM cut-offs on CARDIoGRAM of 744 lead to precision > 0.9 (**Supplementary Fig. 20a)** for CAD. A similar trend was observed when comparing cluster-specific results from endo-RS and endophenotype on UKBB (**Supplementary Fig. 20b)**, with increased precision performances having estimated F-statistic on the same samples where actual endophenotypes where measured. Thus, we adopted those thresholds to define strongly reliable and reliable cluster-specific results in SCZ.

<u>Application of CASTom-iGEx to non-european individuals in UKBB</u>

To test trans-ancestry performances of CASTom-iGEx trained on European samples, we applied CASTom-iGEx pipeline to individuals from UKBB of Indian origins. In particular, we filtered samples using ethnic background (data-field 21000) coded as "Indian" (code 3100), being the largest non white British population. In addition, we removed individuals that withdraw consent, non-imputed ones, having a discordant genetically inferred and reported gender as well as relatives up to 3rd degree. The final cohort included 5,236 individuals among which 461 were satisfying the CAD HARD definition (see Methods). We considered only variants used for CAD analysis in UKBB white British cohort harmonized with GTEx v6p reference panel and CARDIoGRAM cohorts, matched by SNP IDs. On this genotype-only dataset, we imputed gene expression across the 10 CAD related tissues trained on GTEx v6p European samples and performed TWAS and PALAS testing for CAD phenotype. Fraction of concordance based on Z-statistic sign for UKBB

white British (UKBB WB) significant results indicated an overall mild replication (< 0.65) combining all tissues, that however was not significant in some of the tissues (**Supplementary Fig. 23a**) and lower than the replication reached in the European based CARDIoGRAM meta-analysis (**Supplementary Fig. 23b**). In addition, we projected Indian UKBB cohort into the clustering structure computed on UKBB WB in liver. The fraction of cases assigned to each group differed in UKBB Indian from clustering model more than what was observed across CARDIoGRAM cohorts (**Supplementary Fig. 23c-d).** Similarly, the Spear. correlation of cluster-specific genes was different from null but strongly reduced in UKBB Indian compared to CARDIoGRAM (**Supplementary Fig. 23e**). In conclusion, the performances and replications were overall poor when using CASTom-iGEx European trained models on different ancestry population.

<u>Supplementary Note 2</u>

We explicit R^2 as 1 minus the ratio between the variance explained by the model and the original one:

$$1 - \frac{\| \boldsymbol{Y} - \widehat{\boldsymbol{Y}} \|_2^2}{\| \boldsymbol{Y} - \bar{Y} \|_2^2} = \frac{\| \widehat{\boldsymbol{Y}} - \bar{Y} \|_2^2 + 2 \langle \boldsymbol{Y} - \widehat{\boldsymbol{Y}},\ \widehat{\boldsymbol{Y}} - \bar{Y} \rangle}{\sigma_{\boldsymbol{Y}}^2}$$

with $\widehat{\boldsymbol{Y}} \coloneqq X\widehat{\boldsymbol{\beta}} + Z\widehat{\boldsymbol{\mu}}$ the predicted gene expression, $\bar{Y}$ the mean original gene expression, X the cis-variant dosage matrix for the gene in consideration and Z the covariate matrix also including all-one vector to account for intercept term.

Let $\widehat{\boldsymbol{W}} \coloneqq X\widehat{\boldsymbol{\beta}}$ be the predicted genotype effect, $\boldsymbol{W} \coloneqq \boldsymbol{Y} - Z\widehat{\boldsymbol{\mu}}$ the gene expression vector corrected for the confounder effect and $\overline{W}$ the corresponding mean, $\widehat{\boldsymbol{V}} \coloneqq Z\widehat{\boldsymbol{\mu}}$ the predicted

confounder contribution and $\bar{\hat{V}}$ the corresponding mean. Thus by definition, $\boldsymbol{Y} = \boldsymbol{W} + \hat{\boldsymbol{V}}$ and $\bar{Y} = \bar{W} + \bar{\hat{V}}$, hence the first term of R^2 nominator can be written as $\| \hat{\boldsymbol{Y}} - \bar{Y} \|_2^2 = \| \widehat{\boldsymbol{W}} + \hat{\boldsymbol{V}} - \bar{W} - \bar{\hat{V}} \|_2^2 = \| \widehat{\boldsymbol{W}} - \bar{W} \|_2^2 + \| \hat{\boldsymbol{V}} - \bar{\hat{V}} \|_2^2 + 2\langle \widehat{\boldsymbol{W}} - \bar{W}, \hat{\boldsymbol{V}} - \bar{\hat{V}} \rangle$. Since by definition $\boldsymbol{Y} - \hat{\boldsymbol{Y}} = \boldsymbol{W} - \widehat{\boldsymbol{W}}$, the second term of R^2 nominator becomes

$$\langle \boldsymbol{Y} - \hat{\boldsymbol{Y}}, \hat{\boldsymbol{Y}} - \bar{Y} \rangle = \left\langle \boldsymbol{W} - \widehat{\boldsymbol{W}}, \widehat{\boldsymbol{W}} + \hat{\boldsymbol{V}} - \bar{W} - \bar{\hat{V}} \right\rangle = \langle \boldsymbol{W} - \widehat{\boldsymbol{W}}, \widehat{\boldsymbol{W}} - \bar{W} \rangle + \left\langle \boldsymbol{W} - \widehat{\boldsymbol{W}}, \hat{\boldsymbol{V}} - \bar{\hat{V}} \right\rangle$$

Hence, R^2 can be expressed as

$$\frac{\| \widehat{\boldsymbol{W}} - \bar{W} \|_2^2 + 2\langle \boldsymbol{W} - \widehat{\boldsymbol{W}}, \widehat{\boldsymbol{W}} - \bar{W} \rangle + \| \hat{\boldsymbol{V}} - \bar{\hat{V}} \|_2^2 + 2\left\langle \boldsymbol{W} - \bar{W}, \hat{\boldsymbol{V}} - \bar{\hat{V}} \right\rangle}{\sigma_{\boldsymbol{Y}}^2}$$

which we grouped in 3 components R_g^2, R_c^2 and $R_{g,c}^2$.
