# Supplementary material for: Distinct genetic liability profiles define clinically relevant patient strata across common diseases
Source: Nat Commun. 2024 Jul 1;15:5534. doi: 10.1038/s41467-024-49338-2 (PMC11217418; doi:10.1038/s41467-024-49338-2)
Supplement: Supplementary file 3 — Description of Additional Supplementary Files [file 41467_2024_49338_MOESM3_ESM.pdf]

Description of Additional Supplementary Files

File Name: Supplementary Data 1

Description: Significant genes associated with Coronary Artery Disease from TWAS.

Significant genes after tissue-specific multiple testing correction via Benjamini-Hochberg (FDR ≤ 0.05) associated with CAD across 11 tissues. Gene expression models are built from PriLer in GTEx reference panel and imputed in UKBB and CARDIoGRAM cohorts. UKBB is used as discovery dataset composed of 19,026 cases and 321,916 controls, replication results are derived from meta-analysis of 9 CARDIoGRAM cohorts (13,279 cases and 13,402 controls). Each gene is tested via logistic regression correcting for additional covariates. Z-statistic is defined as the gene estimator divided by its standard error $\frac{\beta}{SE\,\beta}$. Each gene is annotated with GWAS from [20] and from matched GWAS in UKBB (Methods) considering the best result around the TSS gene (200kb window). In both cases, GWASs results are considered significant based on FDR correction. Hence, a gene is considered in a new loci if GWAS best hit in that loci is not significant at 0.05 FDR level, divided per published results[20] and matched GWAS respectively.

File Name: Supplementary Data 2

Description: Group-specific Genes for CAD cases clustering in Liver.

Using the clustering structure of CAD cases from UKBB found in liver, the table reports genes that are significant (FDR 0.01) in at least one group across all tissues, tested via two-sided Wilcoxon-Mann-Whitney (WMW) for single a group against remaining cases after normalization. Significance is assessed after tissue-specific and group-specific multiple tested correction via Benjamini-Hochberg procedure. For each tissue, genes are combined into loci based on their genomic position (distance 1Mb of enlarged TSS window ± 200kb). For each

group of genes in a tissue-specific locus, the table shows Z-statistic of the most significant gene for CAD, the groups with significantly different distribution in at least one gene, the common gene across groups with strongest association with a group distribution and the corresponding WMW estimate and p-value.

File Name: Supplementary Data 3

Description: Differences in endophenotypes for groups of CAD cases in Liver.

Group-specific endophenotype analysis using clustering of CAD cases in liver from UKBB with nominal p-value < 0.01. The tested endophenotypes are CAD related classes from UKBB. Differences are tested via Generalized Linear Model (GLM) with phenotype the dependent variable and group-specific clustering structure (gr_i versus remaining cases) and covariates the independent variables. The regression coefficient $\boldsymbol{\beta}$ estimate, standard error and p-value refer to the grouping variable, estimates are corrected for multiple testing in a group-specific manner using Benjamini-Hochberg procedure ("P-value BH corrected" column). The family for GLM applied depends on the phenotype nature (continuous, binary or categorical ordinal). For non-continuous ones it is shown odds ratio and the corresponding 95% confidence interval, for continuous ones instead confidence intervals refer to $\boldsymbol{\beta}$ estimates.

File Name: Supplementary Data 4

Description: Significant pathways associated with Coronary Artery Disease from PALAS.

Significant pathways after tissue-specific and database-specific multiple testing correction via Benjamini-Hochberg (FDR ≤ 0.05) associated with CAD across 11 tissues. The number of genes detected depends on the intersection between genes in a pathway and reliable genes predicted with PriLer in a certain tissue. Each pathway is tested via logistic regression

correcting for additional covariates. Z-statistic is defined as the gene estimator divided by its standard error $\frac{\beta}{SE\,\beta}$. In addition, each pathway is assigned to one of the possible 3 classes: genes P < pathway P i.e. there is at least one gene in that gene-set more significant than the overall pathway; pathway P < genes P & genes FDR > 0.05 i.e. the pathway is more significant than any gene in that gene-set and no genes is significant after multiple testing correction; pathway P < genes P & genes FDR < 0.05 i.e. the pathway is more significant than any gene in that gene-set and at least one gene pass FDR 0.05 threshold.

File Name: Supplementary Data 5

Description: Group-specific Pathways for CAD cases clustering in Liver.

Using the clustering structure of CAD cases from UKBB found in liver, the table reports pathways that are significant (FDR 0.01) in at least one group across all tissues, tested via Wilcoxon-Mann-Whitney (WMW) for single a group against remaining cases after normalization. The pathways are initially clumped based on gene set definition at jaccard similarity threshold 0.2. Significance is assessed after tissue-specific and group-specific multiple tested correction via Benjamini-Hochberg procedure. The table shows Z-statistic from PALAS for CAD, the genes composing the pathway, the groups with significantly different distribution, and the corresponding WMW estimate and p-value. "Improvement wrt genes" indicates for each group if the p-value of the pathway is lower than the p-value reached by the corresponding genes.

File Name: Supplementary Data 6

Description: Nominally significant pathways associated with CAD cluster-specific endophenotypes from PALAS.

Significant pathways (at nominal p-value threshold of 0.01) associated with 24 endophenotypes in UKBB that are cluster-specific in CAD liver clustering, across 10 tissues. The number of genes detected depends on the intersection between genes in a pathway and reliable genes predicted with PriLer in a certain tissue. Each pathway is tested via generalized linear model (gaussian, logistic or ordinal logistic regression, depending on the phenotype) correcting for additional covariates. Z-statistic is defined as the gene estimator divided by its standard error $\frac{\boldsymbol{\beta}}{\boldsymbol{SE\ \beta}}$. Gene-sets databases included are Reactome, Gene Ontology.

File Name: Supplementary Data 7

Description: Hypothesis-driven endophenotype differences for CAD cases clustering in Liver.

Group-specific clinical variables analysis using clustering of CAD cases in liver with nominal p-value < 0.1. For UKBB dataset, clustering of CAD cases in UKBB is considered and 33 clinical variables in UKBB are used without PHESANT preprocessing. For GerMIFSV dataset, clustering structure is obtained via projection from UKBB model clustering and one severeness index annotated for that dataset is tested. Differences are tested via Generalized Linear Model (GLM) with phenotype the dependent variable and group-specific clustering structure (gr_i versus remaining cases) and covariates the independent variables. The regression coefficient $\boldsymbol{\beta}$ estimate, standard error and p-value refer to the grouping variable. The family for GLM applied depends on the phenotype nature (continuous, binary or categorical ordinal). For non-continuous ones it is shown odds ratio and the corresponding 95% confidence interval, for continuous ones instead confidence intervals refer to $\boldsymbol{\beta}$ estimates. Empirical p-value are computed for UKBB variables comparing to endophenotype associations of random clustering repetitions (see Online Methods).

File Name: Supplementary Data 8

Description: Significant genes associated with Schizophrenia from TWAS.

Significant genes after tissue-specific multiple testing correction via Benjamini-Hochberg (FDR $\leq$ 0.05) associated with SCZ across 10 tissues. Gene expression models are built from PriLer in GTEx and CMC reference panels and imputed in 36 European PGC cohorts and CMC dataset itself. Results from meta-analysis in PGC multiple cohorts are used as discovery for a total of 24,764 cases and 30,655 controls, replication results are derived only for DLPC tissue in CMC from CMC dataset itself (212 controls and 266 cases). Each gene is tested via logistic regression correcting for additional covariates. Z-statistic is defined as the gene estimator divided by its standard error $\frac{\boldsymbol{\beta}}{\boldsymbol{SE\ \beta}}$. Each gene is annotated with GWAS best result around its TSS (200kb window) and GWAS results are considered significant based on FDR correction. Hence, a gene is considered in a new locus if GWAS best hit in that loci is not significant at 0.05 FDR level.

File Name: Supplementary Data 9

Description: Significant pathways associated with Schizophrenia from PALAS.

Significant pathways after tissue-specific and database-specific multiple testing correction via Benjamini-Hochberg (FDR $\leq$ 0.05) associated with SCZ across 11 tissues. The number of genes detected depends on the intersection between genes in a pathway and reliable genes predicted with PriLer in a certain tissue. Each pathway is tested via logistic regression correcting for additional covariates. Z-statistic is defined as the gene estimator divided by its standard error $\frac{\boldsymbol{\beta}}{\boldsymbol{SE\ \beta}}$. Gene-sets databases included are Reactome, Gene Ontology, WikiHuman Pathway and a gene-set for DLPC in CMC panel specifically customized in a previous study[7].

File Name: Supplementary Data 10

Description: Group-specific Genes for SCZ cases clustering in Dorsolateral prefrontal cortex.

Using the clustering structure of SCZ cases found in DLPC, the table reports genes that are significant in at least one group across all tissues (FDR 0.01), tested via two-sided Wilcoxon-Mann-Whitney (WMW) for single a group against remaining cases after concatenation and normalization of 35 SCZ cohorts. Significance is assessed after tissue-specific and group-specific multiple tested correction via Benjamini-Hochberg procedure. For each tissue, genes are combined into loci based on their genomic position (distance 1Mb of enlarged TSS window ± 200kb). For each group of genes in a tissue-specific locus, the table shows Z-statistic of the most significant gene for SCZ, the groups with significantly different distribution in at least one gene, the common gene across groups with strongest association with a group distribution and the corresponding WMW estimate and p-value.

File Name: Supplementary Data 11

Description: Group-specific Pathways for SCZ cases clustering in Dorsolateral prefrontal cortex.

Using the clustering structure of SCZ cases from 35 cohorts of PGC2 found in DLPC, the table reports pathways that are significant (FDR 0.01) in at least one group across all tissues, tested via Wilcoxon-Mann-Whitney (WMW) for single a group against remaining cases after normalization. The pathways in Reactome and GO databases are initially clumped based on gene set definition at jaccard similarity threshold 0.2. The pathways from WikiPathway and CMC Gene Set [7] are filtered considering only pathways composed of at least 2 genes. Significance is assessed after tissue-specific and group-specific multiple tested correction via Benjamini-Hochberg procedure, separately for Reactome and GO selection, WikiPathway and

CMC Gene Set. The table shows Z-statistic from PALAS for SCZ, the genes composing the pathway, the groups with significantly different distribution, and the corresponding WMW estimate and p-value. "Improvement wrt genes" indicates for each group if the p-value of the pathway is lower than the p-value reached by the corresponding genes.

File Name: Supplementary Data 12

Description: Differences in gene-risk scores approximating endophenotypes for groups of SCZ cases in Dorsolateral prefrontal cortex.

Group-specific gene-risk scores (gene-RS) analysis using clustering of SCZ cases in DLPC from 35 PGC cohorts. Gene-RS are imputed in each cohort using UKBB estimates for SCZ related phenotypes and genes in DLPC tissue. Differences in gene-RS approximating a phenotype are tested via Generalized Linear Model (GLM) with gene-RS the dependent variable and group-specific clustering structure (gr_i versus remaining cases) and covariates the independent variables. Each cohort is tested separately and results are summarized via meta-analysis. The regression coefficient $\boldsymbol{\beta}$ estimate, standard error, p-value and 95% confidence interval refer to the grouping variable, estimates are corrected for multiple testing in a group-specific manner using Benjamini-Hochberg procedure ("P-value BH corrected" column). The family for GLM applied is always Gaussian due to the continuous nature of gene-RS. Each phenotype-group test is annotated with the corresponding cluster-reliability metric (CRM). The table shows significant and reliable results (FDR < 0.05 and CRM > 744) for all phenotype classes but cognitive ones and only significant (FDR < 0.05) for cognitive classes.

File Name: Supplementary Data 13

Description: Prior features construction in tissue-specific models.

H3K27ac ChIP-Seq and ATAC-Seq prior features as well as GWAS derived for CAD and SCZ. The table contains prior features with corresponding GEO access number and data type used to build tissue specific PriLer gene expression models as well as the tissue-model in which they are included. When multiple features share the same PriLer name, the final prior was built as union of gene regulatory regions.
