# Supplementary material for: Distinct genetic liability profiles define clinically relevant patient strata across common diseases
Source: Nat Commun. 2024 Jul 1;15:5534. doi: 10.1038/s41467-024-49338-2 (PMC11217418; doi:10.1038/s41467-024-49338-2)
Supplement: Supplementary file 17 — Reporting Summary [file 41467_2024_49338_MOESM17_ESM.pdf]

nature portfolio

Corresponding author(s): Michael J. Ziller

Last updated by author(s): Mar 13, 2024

Reporting Summary

Nature Portfolio wishes to improve the reproducibility of the work that we publish. This form provides structure for consistency and transparency in reporting. For further information on Nature Portfolio policies, see our Editorial Policies and the Editorial Policy Checklist.

Statistics

For all statistical analyses, confirm that the following items are present in the figure legend, table legend, main text, or Methods section.

n/a	Confirmed	
☐	☒	The exact sample size (*n*) for each experimental group/condition, given as a discrete number and unit of measurement
☐	☒	A statement on whether measurements were taken from distinct samples or whether the same sample was measured repeatedly
☐	☒	The statistical test(s) used AND whether they are one- or two-sided<br>*Only common tests should be described solely by name; describe more complex techniques in the Methods section.*
☐	☒	A description of all covariates tested
☐	☒	A description of any assumptions or corrections, such as tests of normality and adjustment for multiple comparisons
☐	☒	A full description of the statistical parameters including central tendency (e.g. means) or other basic estimates (e.g. regression coefficient) AND variation (e.g. standard deviation) or associated estimates of uncertainty (e.g. confidence intervals)
☐	☒	For null hypothesis testing, the test statistic (e.g. *F*, *t*, *r*) with confidence intervals, effect sizes, degrees of freedom and *P* value noted<br>*Give P values as exact values whenever suitable.*
☒	☐	For Bayesian analysis, information on the choice of priors and Markov chain Monte Carlo settings
☒	☐	For hierarchical and complex designs, identification of the appropriate level for tests and full reporting of outcomes
☐	☒	Estimates of effect sizes (e.g. Cohen's *d*, Pearson's *r*), indicating how they were calculated

Our web collection on statistics for biologists contains articles on many of the points above.

Software and code

Policy information about availability of computer code

Data collection: No software was used for data collection

Data analysis: Custom code is available at https://gitlab.mpcdf.mpg.de/luciat/castom-igex

Analysis was performed with R (v3.5.3 and v4.0.3)
R packages:

GO.db
MASS
Matrix
PGSEA
RColorBrewer
RNOmni
SparseM
argparse
bigmemory
biomaRt
circlize
coin
cowplot
data.table

doParallel
gep2pep
ggExtra
ggplot2
ggpubr
ggrepel
ggsci
ggsignif
glmnet
gridExtra
igraph
lattice
limma
lme4
lmtest
matrixStats
nloptr
nnet
pROC
pheatmap
pryr
qvalue
rlist
rstatix
stringr
sva
tidyverse
umap

Additional Softwares used: PLINK v2.00a2LM 64-bit Intel, PLINK v1.90b6.10 64-bit, qctool v2.1-dev, PHESANT: https://github.com/astheeggeggs/PHESANT.git, MAGMA v1.10

For manuscripts utilizing custom algorithms or software that are central to the research but not yet described in published literature, software must be made available to editors and reviewers. We strongly encourage code deposition in a community repository (e.g. GitHub). See the Nature Portfolio guidelines for submitting code & software for further information.

Data

Policy information about availability of data

All manuscripts must include a data availability statement. This statement should provide the following information, where applicable:

- Accession codes, unique identifiers, or web links for publicly available datasets
- A description of any restrictions on data availability
- For clinical datasets or third party data, please ensure that the statement adheres to our policy

All the analysis were based on hg19 reference genome.
GEO access number for H3K27ac ChIP-seq data obtained from the Epigenome Roadmap Project and ENCODE is available in Data S1. Additional prior features are obtained from GSE72696 and GSE83345.

Research involving human participants, their data, or biological material

Policy information about studies with human participants or human data. See also policy information about sex, gender (identity/presentation), and sexual orientation and race, ethnicity and racism.

Reporting on sex and gender	Sex was determined by genotype. Individuals showing a discrepancy between reported and genotype sex was excluded (UKBB). Both females and males were included in all analyses.
Reporting on race, ethnicity, or other socially relevant groupings	NA
Population characteristics	All analyses (except Supplementary Fig. 16) were conducted using Caucasian individuals as defined by genotype using pre-defined categories by the data providers mentioned above. In addition, genotype based principal components were used in most analyses as described in detail in the method section.
Recruitment	NA
Ethics oversight	NA

Note that full information on the approval of the study protocol must also be provided in the manuscript.

Field-specific reporting

Please select the one below that is the best fit for your research. If you are not sure, read the appropriate sections before making your selection.

☒ Life sciences ☐ Behavioural & social sciences ☐ Ecological, evolutionary & environmental sciences

For a reference copy of the document with all sections, see nature.com/documents/nr-reporting-summary-flat.pdf

Life sciences study design

All studies must disclose on these points even when the disclosure is negative.

Sample size	Study was based on pre-existing GWAS cohorts with fixed sample size.. Stratification analyses were exploratory with data driven discovery of number of cluster and patient partitioning and thus a priori not known sample size. Stratification analyses were replicated in independent cohorts of lower sample size.
Data exclusions	Data exclusion criteria are described in detail for each analysis in the methods based on various QC criteria for genotype data.
Replication	For the gene expression prediction model via PriLer, replication was assessed from a 5-fold nested cross-validation and R2 on the average outer folders is computed for each gene model. For TWAS and PALAS models, replication of results for CAD built on UK Biobank is assessed on 9 external cohorts from CARDIoGRAM. For SCZ, the replication of results built on PGC cohorts is evaluated on CommonMind Consortium dataset. For clustering results, replication of results on CAD built on UK Biobank is assessed on CARDIoGRAM held-out cohorts as well as SHIP-Trend, in which also differences in imputed gene expression and endophenotypes are evaluated. Replication of results on SCZ based on 35 cohort from PGC is evaluated on 1 PGC held-out cohort (scz_boco_eur) as well as PsyCourse in terms of endophenotype differences.
Randomization	Randomization is used to assess p-value calibration for TWAS and PALAS results in whole-blood. We created binary vectors that resembled CAD phenotype keeping the same case/control size. We selected the same number of females/males and the same age compared to the actual CAD phenotype among the case/control classes.<br>In addition, we randomly partitioned UKBB CAD patients to generate an empirical null-distribution of gene, pathway, and endophenotype associations with clustering structure. The random groups resemble liver-based CAD clustering in group sizes.
Blinding	NA

Reporting for specific materials, systems and methods

We require information from authors about some types of materials, experimental systems and methods used in many studies. Here, indicate whether each material, system or method listed is relevant to your study. If you are not sure if a list item applies to your research, read the appropriate section before selecting a response.

Materials & experimental systems

n/a	Involved in the study
☒	☐ Antibodies
☒	☐ Eukaryotic cell lines
☒	☐ Palaeontology and archaeology
☒	☐ Animals and other organisms
☒	☐ Clinical data
☒	☐ Dual use research of concern
☒	☐ Plants

Methods

n/a	Involved in the study
☒	☐ ChIP-seq
☒	☐ Flow cytometry
☒	☐ MRI-based neuroimaging
