# Supplementary material for: Distinct genetic liability profiles define clinically relevant patient strata across common diseases
Source: Nat Commun. 2024 Jul 1;15:5534. doi: 10.1038/s41467-024-49338-2 (PMC11217418; doi:10.1038/s41467-024-49338-2)
Supplement: Supplementary file 2 — Peer Review File [file 41467_2024_49338_MOESM2_ESM.pdf]

nature portfolio

Peer Review File

Distinct genetic liability profiles define clinically relevant patient strata across common diseases

Open Access This file is licensed under a Creative Commons Attribution 4.0 International License, which permits use, sharing, adaptation, distribution and reproduction in any medium or format, as long as you give appropriate credit to the original author(s) and the source, provide a link to the Creative Commons license, and indicate if changes were made. In the cases where the authors are anonymous, such as is the case for the reports of anonymous peer reviewers, author attribution should be to 'Anonymous Referee' followed by a clear attribution to the source work. The images or other third party material in this file are included in the article's Creative Commons license, unless indicated otherwise in a credit line to the material. If material is not included in the article's Creative Commons license and your intended use is not permitted by statutory regulation or exceeds the permitted use, you will need to obtain permission directly from the copyright holder. To view a copy of this license, visit http://creativecommons.org/licenses/by/4.0/.

Editorial Note: This manuscript has been previously reviewed at another journal that is not operating a transparent peer review scheme. This document only contains reviewer comments and rebuttal letters for versions considered at *Nature Communications.*

Reviewer #1 (Remarks to the Author):

My previous comments are addressed, but I suggest the authors to use the term "polygenic score (PGS)" in place of "polygenic risk score (PRS)", as there is no justification to imply risk or absence of risk for a polygenic score.

Reviewer #2 (Remarks to the Author):

I commend the authors for thoroughly answering all my previous comments. I also appreciate the discussion section noting the caveats and challenges of the analyses presented in this work. I find the revised manuscript improved in clarity and focus by shifting away from the TWAS methodology. As such, I am in full support of its publication.

Rebuttal:

Reviewer #1:

My previous comments are addressed, but I suggest the authors to use the term "polygenic score (PGS)" in place of "polygenic risk score (PRS)", as there is no justification to imply risk or absence of risk for a polygenic score.

We changed the wording of polygenic risk score to polygenic score where appropriate in the manuscript.
